# Piperazine-Thiourea Hybrids as Novel Antiplatelet Agents Targeting COX-1: Synthesis, *in Vitro*, and *in Silico* Evaluation

**DOI:** 10.1021/acsomega.5c12576

**Published:** 2026-04-01

**Authors:** Gabriel Rodrigues Coutinho Pereira, Gil Mendes Viana, Mariana Borges Huber, Pryscila Santiago Rodrigues, Anna Rita Santiago de Paula Gonçalves, Plínio Cunha Sathler, Carlos Rangel Rodrigues, Bárbara de Azevedo Abrahim-Vieira, Lucio Mendes Cabral

**Affiliations:** †Laboratory of Molecular Modeling and QSAR; ‡Laboratory of Industrial Pharmaceutical Technology; §Laboratory of Experimental Hemostasis; 28125 Federal University of Rio de Janeiro, Carlos Chagas Filho Avenue, 373, Rio de Janeiro, 21941-902, Brazil

## Abstract

Cardiovascular diseases (CVDs) remain the leading cause of mortality worldwide, reinforcing the need for safer and more effective antiplatelet therapies. In this work, we designed and synthesized a series of piperazine-derived thioureas under mild conditions and evaluated their antiplatelet potential through *in vitro* and *in silico* approaches. Four derivatives (**3a**, **3g**, **3j**, and **3p**) significantly inhibited arachidonic acid (AA)-induced platelet aggregation in human platelet-rich plasma, achieving levels comparable to aspirin at the same concentration. These compounds showed no anticoagulant effects or hemolytic toxicity, indicating selective action on primary hemostasis and favorable hemocompatibility. ADMET predictions supported their drug-like properties and low toxicity risk. Molecular docking and molecular dynamics simulations revealed stable interactions with key COX-1 residues, while MM-PBSA calculations confirmed favorable binding energies for the most potent derivatives. Our findings revealed that most active piperazine thioureas demonstrated promising potential as novel antiplatelet agents, offering opportunities to improve the quality of life for individuals affected by or at risk of CVDs.

## Graphical abstract

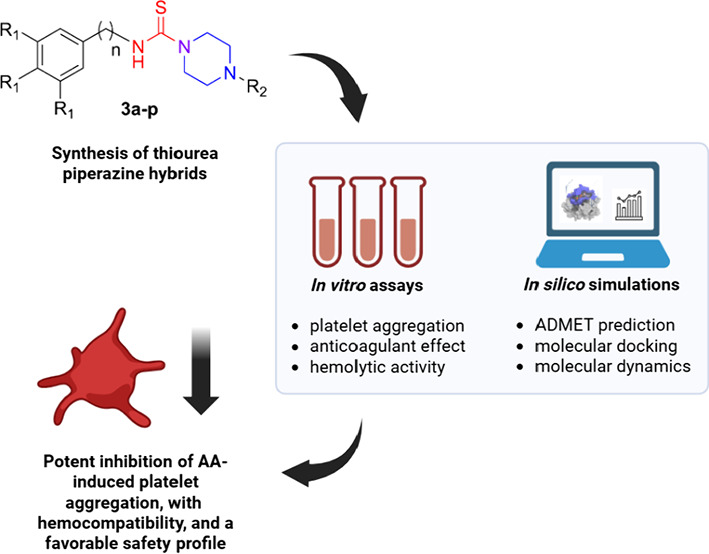

## 1 Introduction

Cardiovascular diseases (CVDs) are multifactorial disorders of congenital or acquired origin that affect the circulatory system.^1^ They encompass four main entities: coronary artery disease (CAD), also known as coronary heart disease (CHD), cerebrovascular disease, peripheral artery disease (PAD), and aortic atherosclerosis.^2^ Although CVD can arise from various etiologies, common risk factors underlying their pathophysiology include smoking, dyslipidemia, hypertension, diabetes, abdominal obesity, psychosocial stress, poor diet, alcohol consumption, and physical inactivity.^3^ In recent decades, these factors have become predominant in society, primarily due to the industrialization of the economy, which has shifted work from physically demanding jobs to more sedentary occupations. Coupled with a consumer-driven, technology-centric culture that promotes longer work hours, extended commutes, and reduced leisure time, these changes have contributed significantly to the dramatic rise in CVD rates.^4^

As a result, CVDs have become the leading cause of mortality worldwide, responsible for an estimated 19.8 million deaths in 2022, equivalent to 1 in 4 deaths globally, with approximately 34% of these fatalities occurring before the age of 70. This burden corresponds to roughly 396 million years of life lost and an additional 44.9 million years lived with disability (YLD).^5^ Furthermore, the economic burden of CVD is significant, making it the costliest of the chronic disorders, surpassing even Alzheimer’s and diabetes. Medical costs and productivity losses associated with CVD are projected to rise from 555 billion USD in 2015 to 1.1 trillion USD by 2035, solely in the United States. This estimate does not include indirect costs, which are currently valued at 237 billion USD annually and are expected to increase, potentially reaching 368 billion USD by 2035.^6^

Atherosclerosis is a central pathological mechanism driving the onset and progression of CVDs. It is characterized by the accumulation of lipids, inflammatory cells, and fibrous tissue within the arterial wall, leading to the formation of plaques that narrow and harden the arteries, impairing blood flow. The rupture or erosion of these plaques trigger thrombosis, i.e., the formation of blood clots, which can either directly obstruct blood vessels or dislodge and embolize to distant sites. This cascade of events exacerbates ischemia and tissue damage, precipitating acute cardiovascular events such as myocardial infarction, ischemic stroke, and peripheral artery disease.^7,8^

As platelets play a central role in thromboembolic events, antiplatelet therapy has long been a cornerstone in the prevention and treatment of most CVDs.^9^ These agents interfere with platelet activation and aggregation by selectively blocking central enzymes involved in the synthesis of platelet agonists, or membrane receptors mediating activation signals, through the following strategies: (i) inhibition of thromboxane A2 synthesis (e.g., COX-1 inhibitors like aspirin); (ii) blockade of adenosine diphosphate (ADP)-mediated signaling (e.g., P2Y12 receptor inhibitors such as clopidogrel, prasugrel, and ticagrelor); (iii) antagonism of integrin αIIbβ3 (GPIIb/IIIa) to prevent fibrinogen binding and platelet aggregation (e.g., abciximab, eptifibatide, and tirofiban); (iv) inhibition of thrombin-induced platelet activation via the PAR1 receptor (e.g., vorapaxar); (v) modulation of phosphodiesterase activity to increase intracellular cyclic AMP levels (e.g., dipyridamole and cilostazol).^10^

Despite significant advances in antithrombotic therapies, including antiplatelet agents, bleeding remains a persistent and potentially life-threatening concern. Other serious adverse effects, including neutropenia and thrombocytopenia, are also associated with these drugs, complicating their use, especially in vulnerable patient populations. The considerable variability in individual responses to treatment, along with the development of tolerance over time, further highlights the limitations of current therapies. These challenges underscore the urgent need for continued research into novel antithrombotic agents.^9,11^

In this context, thiourea derivatives have attracted attention for their antithrombotic potential.^12–16^ In a previous study by our research group, Lourenço et al. (2015) synthesized and tested a series of *N*,*N*’-disubstituted thioureas, initially assessing their potency against arachidonic acid (AA)-induced platelet aggregation, which is a potent inducer of platelet activation. The compounds exhibited significant inhibition (96%–98%), comparable to aspirin (97.5%) at the same concentration (100 μM). Further analysis revealed that these thioureas effectively inhibited COX-1 activity, as demonstrated by their impact on PGE2 and TXB2 production, well-established markers of COX-1 activity, showing inhibition rates like the COX inhibitor indomethacin. Conversely, aspirin exhibited significantly higher PGE2 inhibition, possibly attributed to its irreversible COX-1 inhibition through selective acetylation of Ser530.^13^

Building on the promising results with thiourea derivatives (Figure 1), we explore additional structural modifications to further enhance their antiplatelet activity. The incorporation of the piperazine group offers a compelling strategy, as this heterocyclic ring is present in a variety of molecules with proven effectiveness *in vitro* against arachidonic acid (AA)-induced platelet aggregation^17–21^ in addition to demonstrating significant COX-1 inhibitory potency.^22–24^ In this study, we therefore propose the design of novel antiplatelet agents through the incorporation of piperazine into the thiourea structure. By leveraging the synergistic effects of both structural components,^25^ our primary goal was to improve the efficiency of thiourea-based antiplatelet agents.

**Figure 1:**
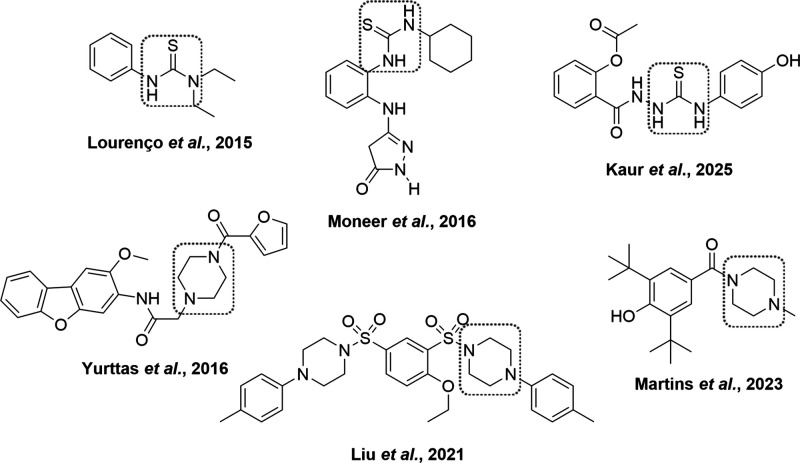
Thiourea and piperazine-based derivatives reported in the literature showing *in vitro* COX-1 inhibition and antiplatelet activity on arachidonic-acid (AA)–induced platelet aggregation.

To this end, a novel series of piperazine thioureas was designed and synthesized. We then evaluated *in vitro* their activity against arachidonic acid (AA)-induced platelet aggregation, conducted a robust *in silico* characterization of their pharmacokinetics and toxicological profiles, and simulated their interactions with the COX-1 enzyme.

## 2 Materials and Methods

### 2.1 Synthesis of Piperazine Thioureas

#### 2.1.1 General Information

Reagents and solvents were obtained commercially and used without further purification. Melting points were determined using a Shimadzu DSC-60 differential scanning calorimeter, operating at a heating rate of 10 °C/min over the temperature range of 30–200 °C, under a constant nitrogen flow of 50 mL/min, and using an aluminum standard. Infrared spectra were recorded on a Nicolet Magna-IR 760 spectrophotometer employing KBr pellets. The ^1^H and ^13^C NMR spectra were acquired on Bruker Avance spectrometers operating at 400 or 500 MHz, using CDCl_3_ or DMSO-*d*_*6*_ as solvent. Chemical shifts (δ) are expressed in parts per million (ppm) and coupling constants (*J*) in hertz (Hz). High-resolution mass spectrometric (HRMS) data were obtained using a Bruker ESI-qTOF mass spectrometer. Thin-layer chromatography (TLC) analyses were performed on silica gel 60 F_254_ aluminum plates (Merck), employing ethyl acetate/hexane (1:4 v/v) as the mobile phase. Visualization of the chromatographic spots was achieved under UV light or by spraying with a 5% (w/v) ethanolic solution of phosphomolybdic acid followed by gentle heating.

#### 2.1.2 General Procedure for the Synthesis of Piperazine Thioureas

A solution of the appropriate isothiocyanate **1** (1.1 mmol) and piperazine derivative **2** (1.0 mmol) in dichloromethane (20 mL) was stirred at room temperature for 1 h. Upon completion of the reaction, as monitored by TLC, the solvent was removed under reduced pressure, and the crude material was washed three times with hexane, taking advantage of the higher solubility of the unreacted isothiocyanate in this solvent. The resulting solids were collected to afford the corresponding thiourea derivatives in good yields, typically without further purification. All final compounds were fully characterized by ^1^H and ^13^C NMR, IR spectroscopy, and high-resolution mass spectrometry (HRMS), and their melting points were recorded. The purity of all products was judged to be >95% based on NMR analysis.

### N,4-Diphenylpiperazine-1-carbothioamide (3a)

^26^ White solid; 160.38 °C; IR (KBr): 3160, 2820, 1599, 1529, 1320, 1220, 1025, 936, 753, 707, 517 cm^–1^; ^1^H NMR (400 MHz, CDCl_3_) δ 7.46 (br s, 1H), 7.39–7.21 (m, 4H), 7.20–7.09 (m, 3H), 6.97–6.82 (m, 3H), 3.96 (t, *J* = 5.10 Hz, 4H), 3.24 (t, *J* = 5.10 Hz, 4H); ^13^C NMR (100 MHz, CDCl_3_) δ 183.42, 150.54, 140.12, 129.44, 129.32, 125.48, 123.41, 120.47, 116.26, 49.28, 48.62; HR-MS-ESI: *m*/*z* [M + Na]^+^ calculated for C_17_H_19_N_3_S: 320.1192; found: 320.1196.

### N-Benzyl-4-phenylpiperazine-1-carbothioamide (3b)

^27^ White solid; 180.33 °C; IR (KBr): 3251, 2821, 1600, 1546, 1504, 1417, 1338, 1236, 1060, 1014, 869, 755, 701, 555 cm^–1^; ^1^H NMR (400 MHz, CDCl_3_) δ 7.37–7.24 (m, 7H), 7.08–6.97 (m, 3H), 6.17 (br s, 1H), 4.88 (d, *J* = 4.80 Hz, 2H), 4.14–4.06 (m, 4H), 3.30 (t, *J* = 4.98 Hz, 4H); ^13^C NMR (100 MHz, CDCl_3_) δ 182.43, 148.39, 137.94, 129.61, 128.81, 128.13, 127.76, 122.43, 117.04, 50.29, 49.76, 46.75; HR-MS-ESI: *m*/*z* [M + Na]^+^ calculated for C_18_H_21_N_3_S: 334.1340; found: 334.1344.

### N-Phenethyl-4-phenylpiperazine-1-carbothioamide (3c)

White solid; 141.97 °C; IR (KBr): 3309, 2934, 1600, 1539, 1379, 1228, 1004, 930, 868, 691, 524 cm^–1^; ^1^H NMR (400 MHz, CDCl_3_) δ 7.39–7.16 (m, 7H), 6.95–6.80 (m, 3H), 5.53 (br t, 1H), 4.03–3.82 (m, 6H), 3.23 (t, *J* = 5.3 Hz, 4H), 2.97 (t, *J* = 6.8 Hz, 2H); ^13^C NMR (100 MHz, CDCl_3_) δ 182.13, 150.49, 138.99, 129.43, 128.96, 128.92, 126.83, 120.24, 115.95, 48.38, 47.07 (2C), 35.29; HR-MS-ESI: *m*/*z* [M + Na]^+^ calculated for C_19_H_23_N_3_S: 348.1505; found: 348.1503.

### 4-Phenyl-N-(3,4,5-trimethoxyphenyl)­piperazine-1-carbothioamide (3d)

White solid; 204.90 °C; IR (KBr): 3186, 2946, 2819, 1605, 1509, 1492, 1328, 1219, 1131, 1032, 1000, 932, 772 cm^–1^; ^1^H NMR (400 MHz, CDCl_3_) δ 7.36 (br s, 1H),7.32–7.25 (m, 2H), 6.95–6.87 (m, 3H), 6.45 (s, 2H), 4.01 (t, *J* = 4.6 Hz, 4H), 3.85–3.79 (m, 9H), 3.28 (t, *J* = 4.6 Hz, 4H); ^13^C NMR (100 MHz, CDCl_3_) δ 183.14, 153.45, 150.38, 135.77, 135.66, 129.34, 120.48, 116.18, 101.31, 60.98, 56.20, 49.04, 48.64; HR-MS-ESI: *m*/*z* [M + H]^+^ calculated for C_20_H_25_N_3_O_3_S: 388.1689; found: 388.1689.

### 4-(4-Hydroxyphenyl)-*N*-phenylpiperazine-1-carbothioamide (3e)

^27^ White solid; 196.83 °C; IR (KBr): 3230, 2823, 1589, 1513, 1444, 1322, 1270, 1213, 1139, 1020, 931, 761, 700, 516 cm^–1^; ^1^H NMR (400 MHz, DMSO-*d*_*6*_) δ 9.40 (br s, 1H), 8.89 (br s, 1H), 7.35–7.27 (m, 4H), 7.14–7.07 (m, 1H), 6.84 (d, *J* = 8.70 Hz, 2H), 6.68 (d, *J* = 8.70 Hz, 2H), 4.09–3.97 (m, 4H), 3.09–2.98 (m, 4H); ^13^C NMR (100 MHz, DMSO-*d*_*6*_) δ 182.01, 151.83, 144.07, 141.50, 128.47, 125.68, 124.76, 118.71, 116.01, 50.43, 48.49; HR-MS-ESI: *m*/*z* [M + H]^+^ calculated for C_17_H_19_N_3_OS: 314.1322; found: 314.1326.

### N-Benzyl-4-(4-hydroxyphenyl)­piperazine-1-carbothioamide (3f)

Brown solid; 164.61 °C; IR (KBr): 3630, 3259, 2827, 2806, 1541, 1516, 1375, 1286, 1159, 1020, 960, 815, 726, 695 cm^–1^; ^1^H NMR (500 MHz, DMSO-*d*_6_) δ 8.88 (br s, 1H), 8.32 (t, J = 5.6 Hz, 1H), 7.32–7.27 (m, 4H), 7.24–7.18 (m, 1H), 6.82 (d, J = 8.9 Hz, 2H), 6.67 (d, J = 8.9 Hz, 2H), 4.82 (d, J = 5.6 Hz, 2H), 3.95 (t, J = 4.9 Hz, 4H), 2.98 (t, J = 4.9 Hz, 4H); ^13^C NMR (125 MHz, DMSO-*d*_6_) δ 182.32; 151.73; 144.19; 140.17; 128.56; 127.64; 127.03; 118.63; 115.97; 50.37; 48.82; 47.89; HR-MS-ESI: *m*/*z* [M + H]^+^ calculated for C_18_H_21_N_3_OS: 328.1478; found: 328.1490.

### 4-(4-Hydroxyphenyl)-N-phenethylpiperazine-1-carbothioamide (3g)

White solid; 191.55 °C; IR (KBr): 3376, 3226, 2938, 1552, 1518, 1414, 1342, 1233, 1160, 1096, 1003, 927, 820, 703 cm^–1^; ^1^H NMR (400 MHz, DMSO-*d*_*6*_) δ 8.86 (br s, 1H), 7.87 (br t, *J* = 4.9 Hz, 1H), 7.37–7.16 (m, 5H), 6.81 (d, *J* = 8.8 Hz, 2H), 6.67 (d, *J* = 8.8 Hz, 2H), 3.94–3.85 (m, 4H), 3.75–3.67 (m, 2H), 3.01–2.91 (m, 4H), 2.87 (t, *J* = 7.7 Hz, 2H); ^13^C NMR (100 MHz, DMSO-*d*_*6*_) δ 181.75, 151.74, 144.21, 140.02, 129.11, 128.81, 126.52, 118.64, 115.97, 50.33, 47.61, 47.31, 35.27; HR-MS-ESI: *m*/*z* [M + H]^+^ calculated for C_19_H_23_N_3_OS: 342.1635; found: 342.1633.

### 4-(4-Hydroxyphenyl)-N-(3,4,5-trimethoxyphenyl)­piperazine-1-carbothioamide (3h)

^27^ White solid; 236.51 °C; IR (KBr): 3438, 3158, 2940, 1604, 1508, 1463, 1322, 1216, 1126, 1031, 931, 840, 728, 555 cm^–1^; ^1^H NMR (500 MHz, DMSO-*d*_*6*_) δ 9.30 (br s, 1H), 8.88 (br s, 1H), 6.83 (d, *J* = 8.88 Hz, 2H), 6.69–6.65 (m, 4H), 4.00 (t, *J* = 4.65 Hz, 4H), 3.73 (s, 6H), 3.64 (s, 3H), 3.03 (t, *J* = 4.65 Hz, 4H); ^13^C NMR (125 MHz, DMSO-*d*_*6*_) δ 181.70, 152.65, 151.78, 144.12, 137.24, 134.89, 118.66, 115.99, 103.56, 60.54, 56.31, 50.40, 48.45; HR-MS-ESI: *m*/*z* [M + H]^+^ calculated for C_20_H_25_N_3_O_4_S: 404.1631; found: 404.1638.

### 4-(2-Chlorophenyl)-*N*-phenylpiperazine-1-carbothioamide (3i)

^27^ White solid; 175.78 °C; IR (KBr): 3222, 2809, 1585, 1529, 1479, 1411, 1307, 1218, 1172, 1022, 943, 761, 721, 597 cm^–1^; ^1^H NMR (500 MHz, CDCl_3_) δ 7.49 (br s, 1H), 7.39–7.31 (m, 3H), 7.27–7.20 (m, 1H), 7.17–7.11 (m, 3H), 7.03–6.98 (m, 2H), 3.98 (t, *J* = 4.53 Hz, 4H), 3.08 (t, *J* = 4.84 Hz, 4H); ^13^C NMR (125 MHz, CDCl_3_) δ 183.57, 148.30, 140.05, 130.75, 129.25, 128.90, 127.76, 125.20, 124.43, 122.83, 120.56, 50.69, 49.88; HR-MS-ESI: *m*/*z* [M + H]^+^ calculated for C_17_H_18_ClN_3_S: 332.0913; found: 332.0982.

### N-Benzyl-4-(2-chlorophenyl)­piperazine-1-carbothioamide (3j)

^28^ White solid; 111.40 °C; IR (KBr): 3295, 2898, 2819, 1589, 1548, 1481, 1322, 1222, 1145, 1022, 968, 863, 738, 686, 549 cm^–1^; ^1^H NMR (500 MHz, CDCl_3_) δ 7.38–7.33 (m, 5H), 7.32–7.27 (m, 1H), 7.26–7.20 (m, 1H), 7.03–6.98 (m, 2H), 5.80 (br s, 1H), 4.89 (d, *J* = 4.77 Hz, 2H), 3.99 (t, *J* = 5.00 Hz, 4H), 3.08 (t, *J* = 5.00 Hz, 4H); ^13^C NMR (125 MHz, CDCl_3_) δ 182.46, 148.34, 137.85, 130.76, 128.87, 128.18, 127.85, 127.77, 124.41, 120.50, 50.73, 50.47, 47.79; HR-MS-ESI: *m*/*z* [M + Na]^+^ calculated for C_18_H_20_ClN_3_S: 368.0889; found: 368.0958.

### 4-(2-Chlorophenyl)-N-phenethylpiperazine-1-carbothioamide (3k)

^28^ White solid; 111.76 °C; IR (KBr): 3301, 2939, 2809, 1587, 1527, 1479, 1346, 1228, 1124, 1008, 931, 862, 742, 701, 493 cm^–1^; ^1^H NMR (500 MHz, CDCl_3_) δ 7.36 (dd, *J* = 1.45 and 7.80 Hz, 1H), 7.32 (t, *J* = 7.46 Hz, 2H), 7.25–7.20 (m, 4H), 7.02–6.97 (m, 2H), 5.58 (t, *J* = 4.57 Hz, 1H), 3.96 (q, *J* = 6.80 Hz, 2H), 3.89 (t, *J* = 5.02 Hz, 4H), 3.04 (t, *J* = 5.07 Hz, 4H), 2.97 (t, *J* = 6.90 Hz, 2H); ^13^C NMR (125 MHz, CDCl_3_) δ 182.30, 148.36, 138.88, 130.75, 128.88, 128.85, 128.78, 127.76, 126.69, 124.40, 120.49, 50.69, 47.53, 46.95, 35.16; HR-MS-ESI: *m*/*z* [M + H]^+^ calculated for C_19_H_22_ClN_3_S: 360.1226; found: 360.1295.

### 4-(2-Chlorophenyl)-N-(3,4,5-trimethoxyphenyl)­piperazine-1-carbothioamide (3l)

^28^ White solid; 180.66 °C; IR (KBr): 3160, 2813, 2811, 1602, 1479, 1317, 1216, 1124, 1027, 931, 813, 730, 615, 439 cm^–1^; ^1^H NMR (500 MHz, CDCl_3_) δ 7.43 (br s, 1H), 7.38 (d, *J* = 7.75 Hz, 1H), 7.24 (t, *J* = 7.75 Hz, 1H), 7.02 (t, *J* = 7.75 Hz, 2H), 6.46 (s, 2H), 4.03 (t, *J* = 4.70 Hz, 4H), 3.83 (s, 9H), 3.11 (t, *J* = 4.70 Hz, 4H); ^13^C NMR (125 MHz, CDCl_3_) δ 183.25, 153.43, 148.24, 135.89, 135.54, 130.78, 128.88, 127.79, 124.48, 120.48, 101.16, 60.98, 56.21, 50.75, 49.61; HR-MS-ESI: *m*/*z* [M + Na]^+^ calculated for C_20_H_24_ClN_3_O_3_S: 444.1125; found: 444.1119.

### 4-Benzhydryl-*N*-phenylpiperazine-1-carbothioamide (3m)

^28^ White solid; 216.60 °C; IR (KBr): 3440, 3193, 2798, 1594, 1513, 1450, 1324, 1226, 1035, 995, 929, 757, 692, 609 cm^–1^; ^1^H NMR (CDCl_3_) δ 7.39 (d, *J* = 7.57 Hz, 4H), 7.31–7.23 (m, 7H), 7.17 (t, *J* = 7.18 Hz, 2H), 7.10 (t, *J* = 7.35 Hz, 1H), 7.06 (d, *J* = 7.79 Hz, 2H), 4.23 (s, 1H), 3.78 (br s, 4H), 2.42 (t, *J* = 4.54 Hz, 4H); ^13^C NMR (CDCl_3_) δ 183.12, 142.00, 140.12, 129.17, 128.66, 127.84, 127.25, 125.03, 122.70, 75.78, 51.28, 49.78; HR-MS-ESI: *m*/*z* [M + H]^+^ calculated for C_24_H_25_N_3_S: 388.1835; found: 388.1842.

### 4-Benzhydryl-N-benzylpiperazine-1-carbothioamide (3n)

^28^ White solid; 156.54 °C; IR (KBr): 3315, 3021, 2800, 1598, 1535, 1450, 1330, 1220, 1143, 997, 879, 732, 694, 609, 453 cm^–1^; ^1^H NMR (CDCl_3_) δ 7.38 (d, *J* = 7.45 Hz, 4H), 7.33–7.23 (m, 9H), 7.17 (t, *J* = 7.26 Hz, 2H), 5.64 (br s, 1H), 4.82 (d, *J* = 4.77 Hz, 2H), 4.22 (s, 1H), 3.77 (t, *J* = 4.86 Hz, 4H), 2.41 (t, *J* = 4.86 Hz, 4H); ^13^C NMR (CDCl_3_) δ 182.11, 142.00, 137.98, 128.82, 128.67, 128.09, 127.86, 127.76, 127.27, 75.76, 51.29, 50.32, 47.62; HR-MS-ESI: *m*/*z* [M + H]^+^ calculated for C_25_H_27_N_3_S: 402.1992; found: 402.1998.

### 4-Benzhydryl-N-phenethylpiperazine-1-carbothioamide (3o)

^28^ White solid; 138.93 °C; IR (KBr): 3345, 2927, 2805, 1540, 1407, 1338, 1238, 1143, 997, 881, 734, 705, 593, 499 cm^–1^; ^1^H NMR (CDCl_3_) δ 7.39 (d, *J* = 7.47 Hz, 4H), 7.30–7.23 (m, 6H), 7.22–7.15 (m, 5H), 5.43 (br s, 1H), 4.21 (s, 1H), 3.90 (q, *J* = 6.30 Hz, 2H), 3.68 (t, *J* = 4.91 Hz, 4H), 2.91 (t, *J* = 6.89 Hz, 2H), 2.38 (t, *J* = 5.02 Hz, 4H); ^13^C NMR (CDCl_3_) δ 181.91, 142.04, 138.89, 128.81, 128.76, 128.66, 127.85, 127.25, 126.65, 75.79, 51.27, 47.33, 46.82, 35.16; HR-MS-ESI: *m*/*z* [M + H]^+^ calculated for C_26_H_29_N_3_S: 416.2148; found: 416.2154.

### 4-Benzhydryl-N-(3,4,5-trimethoxyphenyl)­piperazine-1-carbothioamide (3p)

^28^ White solid; 151.96 °C; IR (KBr): 3415, 2917, 2805, 1596, 1504, 1317, 1228, 1126, 995, 892, 823, 705, 609 cm^–1^; ^1^H NMR (400 MHz, CDCl_3_) δ 7.43–7.36 (m, 4H), 7.31–7.15 (m, 7H), 6.36 (s, 2H), 4.25 (s, 1H), 3.85–3.76 (m, 13H), 2.44 (t, *J* = 4.80 Hz, 4H); ^13^C NMR (100 MHz, CDCl_3_) δ 183.00, 153.43, 141.82, 135.97, 135.49, 128.66, 127.87, 127.28, 100.88, 75.76, 60.95, 56.17, 51.29, 49.62; HR-MS-ESI: *m*/*z* [M + H]^+^ calculated for C_27_H_31_N_3_O_3_S: 478.2153; found: 478.2159.

### 2.2 Biological Assays

#### 2.2.1 *In Vitro* Platelet Aggregation Assays

Blood samples were collected from healthy adult volunteers via venipuncture into vacuum tubes containing 3.2% buffered sodium citrate, in accordance with the guidelines approved by the Ethics Committee (protocol n° 3.807.671, UFRJ, Brazil). The samples were centrifuged at 800 rpm for 10 min to obtain platelet-rich plasma (PRP). The remaining blood was then centrifuged at 4,000 rpm for 10 min to obtain platelet-poor plasma (PPP).^29^ Platelet aggregation assays were performed using a Chrono-log 560 VS aggregometer. The test compounds (100 μM) were preincubated in cuvettes with PRP for 2 min before platelet aggregation was induced using arachidonic acid (500 μM). To determine the IC_50_ values, different concentrations of the compounds were tested, representing the concentration required to inhibit 50% of arachidonic acid-induced platelet aggregation. All platelet aggregation assays were conducted in triplicate. Acetylsalicylic acid (ASA, Sigma) was used as the positive control, while 1% dimethyl sulfoxide (DMSO, Sigma) served as the negative control.^30^

#### 2.2.2 *In Vitro* Coagulation Assays

Platelet-poor plasma (PPP) obtained from the platelet aggregation assay was used for prothrombin time (PT) and activated partial thromboplastin time (aPTT) tests. Coagulation assays were performed using the CoagLab IV coagulation analyzer (Beijing Shining Sun Technology Co., Ltd., Beijing, China) according to the manufacturer’s instructions (STAGO Diagnóstica). Anticoagulant activity was assessed by calculating the ratio of the clotting time of the test sample to that of the control [T1/T0 (seconds)].^31^ The negative control consisted of 1% dimethyl sulfoxide (DMSO, Sigma).

#### 2.2.3 Hemolysis Assay

Blood samples were centrifuged at 2500 rpm for 10 min, and the erythrocytes were washed three times with phosphate-buffered saline (PBS, pH 7.4). An erythrocyte suspension was then prepared by repeated centrifugation and resuspension in the same buffer. The suspension was incubated in microtubes with the test compounds (100 μM) for 3 h at 37 °C. Hemoglobin release was quantified using a microplate reader (KASUAKI) at 546 nm. The assay was performed in triplicate, with complete hemolysis induced by 1% Triton X-100 serving as the positive control. Hemolysis values of ≤ 10% were considered indicative of hemocompatibility and nontoxicity of the tested derivatives.^13^

### 2.3 *In Silico* Pharmacokinetics and Toxicological Assessment

The SMILES (Simplified Molecular Input Line Entry System) notation of the active thioureas (**3a**, **3g**, **3j**, and **3p**) were input into the ADMET Predictor XII software (Simulations Plus, Inc., Lancaster, CA, USA) to perform an *in silico* characterization of their pharmacokinetic and toxicological profiles, collectively known by the acronym ADMET (Absorption, Distribution, Metabolism, Excretion, and Toxicity).

The software incorporates accurate QSAR (Quantitative Structure–Activity Relationship) models–typically achieving R^2^ between 0.7 and 0.9–into a robust risk assessment framework. By analyzing data from 2,260 commercial drugs listed in the World Drug Index (WDI),^32^ ADMET Predictor sets thresholds for safety and pharmacokinetic properties, penalizing compounds that surpass these limits. The overall ADMET risk factor is determined by combining outputs from three different statistical models—TOX risk (toxicity), Absn risk (absorption), and CYP risk (metabolism)—along with two key parameters for distribution and excretion: volume of distribution (Vd) and fraction unbound (fu). This integrated methodology provides a thorough analysis of the pharmacokinetic and toxicity profiles of new drug candidates.

The TOX risk model combines the following end points: hepatotoxicity, mutagenicity, carcinogenicity, cardiotoxicity, and acute toxicity. Hepatotoxicity was evaluated by estimating serum levels of liver-damage-related enzymes, including alanine aminotransferase (ALT), aspartate aminotransferase (AST), lactate dehydrogenase (LDH), alkaline phosphatase (ALP), and gamma-glutamyltransferase (GGT).^33^

Mutagenicity was initially assessed using the MUT risk systems, which employs a variety of Ames’s test^34^ models to simulate *in silico* assays with *Salmonella typhimurium strains* (TA97, TA98, TA100, TA1535, and TA1537) and the repair-deficient *Escherichia coli* strain WP2, both with and without metabolic activation by the S9 fraction. Mutagenicity was further evaluated using the MUTx risk model, an enhanced version of MUT risk, which incorporates interaction rules for the mutagenicity assays previously mentioned. This improvement reflects the existing correlations between strains, resulting in enhanced sensitivity and specificity compared to MUT risk standard model, while maintaining the same toxicity threshold.

Acute rat toxicity was estimated by determining the oral dose required to kill half of the rat population within 24 h. Cardiotoxicity, in turn, was assessed using a model based on hERG (Ether-à-go-go Related Gene) channel inhibition, a key marker of potential cardiac risk. Finally, carcinogenicity was evaluated using rat- and mouse-based models, considering the chronic oral daily dose needed to induce tumors in half of the population.^35^

Absorption risk (Absn risk) was evaluated to detect potential oral absorption issues in the piperazine thiourea based on their physicochemical properties. The following physicochemical parameters were included in this evaluation: molecular weight (MW, g/mol), octanol–water partition coefficient (log P), octanol–water distribution coefficient at pH 7.4 (log D), topological polar surface area (TPSA), hydrogen bond donor groups (HBD), hydrogen bond acceptor groups (HBA), number of rotatable bonds, and the ionization constant (p*K*_a_) for the most basic group. Violations of Lipinski’s rule of five were also checked for further characterization of drug-likeness.^36^

Finally, metabolic risk (CYP risk) was predicted based on the compound’s oxidation by CYP enzymes. CYP risk was assigned to compounds with elevated levels of intrinsic clearance calculated using recombinant assays (CYP1A2, CYP2C9, CYP2C19, CYP2D6, CYP3A4) and a human microsomal model (CL).^35^

### 2.4 Molecular Docking

The experimental structure of COX-1 from *Homo sapiens* (hCOX-1) was retrieved from the Protein Data Bank (PDB) under the identification code 6Y3C.^37^ This structure, which is the only hCOX-1 available to date, is not complexed with any ligand.^38^

As previously demonstrated, redocking validation on conserved sites of homologous proteins provides a consistent indication of the docking algorithm’s performance.^39^ Thus, to validate the docking protocol within the active site of COX-1, we initially performed a redocking procedure using the *Ovis aries* COX-1 (oCOX-1) structure complexed with mofezolac (PDB ID: 5WBE),^40^ a potent selective COX-1 inhibitor.^41^ hCOX-1 shares a high sequence identity (92%) with oCOX-1, along with remarkable conservation of the residues in their active sites.^42^

The docking protocol was initially validated through a redocking procedure using the ovine cyclooxygenase-1 (oCOX-1) structure cocrystallized with the selective inhibitor mofezolac (PDB ID: 5WBE), because no human COX-1 (hCOX-1) structures were available in complex with ligands. As a second validation step, mofezolac was docked into the cyclooxygenase active site of hCOX-1 to ensure the reproducibility of its binding mode within the human enzyme and to verify the transferability of the protocol across species. Together, these two validation steps establish the reliability of the docking methodology for hCOX-1. Accordingly, all subsequent molecular docking simulations of the newly designed piperazine–thiourea derivatives were performed exclusively using the human cyclooxygenase-1 structure (PDB ID: 6Y3C).^39,43^ The GOLD software 2022.3.0 ^44^ was employed for the (re)­docking procedure. The system was prepared by adding hydrogen atoms to COX-1 residues using the ProteinPrepare server,^45^ and to mofezolac using OpenBabel 3.1.1,^46^ considering the physiological pH of 7.4. The genetic algorithm (GA) was defined as the search algorithm, while GoldScore was selected as the scoring function. GoldScore is an empirical function optimized for predicting binding affinity, considering energy terms from hydrogen bonds, van der Waals interactions, metal interactions, and ligand torsions. It has shown high correlation with experimental affinity values in benchmark analyses, outperforming other available methods in various investigated scenarios.^47^ Then, a grid box with dimensions of 10Å was positioned in the active site, centered on ligand, which corresponds to the coordinates: x = −20.490; y = 50.288; z = 9.939.

After system preparation, (re)­docking simulations were conducted using the semiflexible approach, considering the ligand as flexible and the protein as rigid. The conformational search was conducted with 100 runs of the genetic algorithm, using the default parameters of Gold 2022.3.0, i.e., initial population: 100 individuals; selective pressure rate: 1.1; minimum number of operations: 10,000; maximum number of operations: 125,000; number of islands: 5; niche size: 2; crossover rate: 95; mutation rate: 95; migration frequency: 10; conformational search efficiency: 100%. Early stopping was configured to occur when the top three solutions differ by less than 1.5 Å. The solution with the highest GoldScore value was selected to represent the simulation.^43^

The redocking was considered validated when the predicted pose for the ligand deviated less than 2 Å from the pose observed in the experimental complex, along with the conservation of the interaction profile, verified through visual inspection in 3D^48^ in Pymol 3.0.2.^49^ Once validated, the established docking protocol was applied to simulate the potential binding modes of the active thioureas **3a**, **3g**, **3j**, and **3p** in the hCOX-1 receptor (PDB ID: 6Y3C). The inactive thioureas, i.e., **3b**, **3c**, **3d**, **3e**, **3f**, **3h**, **3i**, **3k**, **3l**, **3m**, **3n**, and **3o** were subsequently docked using the same validated protocol to evaluate their interaction profiles and binding orientations within the cyclooxygenase site. These derivatives were designed in the OpenBabel 3.1.1 software^46^ and minimized with the MMFF94s force field.^50^ The generated three-dimensional structures were saved in *.sdf format and used as input files for docking simulations.

### 2.5 Molecular Dynamics Simulations

The docking solutions predicted for the active thioureas **3a**, **3g**, **3j**, and **3p** within the active site of hCOX-1 were used as input files for molecular dynamics (MD) simulations. The ligands were initially submitted to the SwissParam server to generate CHARMM-based topologies.^51^ The CHARMM27 force field was then selected for the simulations, which were performed using the GROMACS 2020.6 package.^52^ Simulations of apo hCOX-1 were also performed to provide a baseline for evaluating ligand-induced effects. All simulations were conducted without the heme prosthetic group, in accordance with the crystallographic structure of hCOX-1 (PDB ID: 6Y3C).^37^ Once prepared, the complexes were centered in triclinic boxes with a margin of 1.0 nm from its surface, ensuring adequate solvation by TIP3P water molecules. The systems were neutralized by adding Na^+^ and Cl^–^ ions at a concentration of 0.15 mol/L, and subsequently minimized using the steepest descent method.^53^

In all simulations, the prepared hCOX-1 protein consisted of 557 amino acid residues, totaling 8,945 protein atoms. The ligands included the following number of atoms: **3a**, 39 atoms; **3g**, 46 atoms; **3j**, 42 atoms; and **3p**, 66 atoms. The number of sodium and chloride ions added to neutralize the systems was typically 58 Na^+^ and 58 Cl^–^, except for the hCOX1-**3p** complex, which contained 59 Cl^–^ ions. The number of water molecules solvating the complexes was 18,017, 18,030, 18,012, and 18,025 for **3a**, **3g**, **3j**, and **3p**, respectively. The apo hCOX-1 system was prepared similarly, comprising 8,945 protein atoms, 58 Na^+^ and 58 Cl^–^ ions, and 18,038 water molecules.After energy minimization, equilibration was performed using NVT (constant number of particles, volume, and temperature) and NPT (constant number of particles, pressure, and temperature) ensembles. The NVT ensemble was conducted for 100 ps at 310 K using the v-rescale thermostat, while the NPT ensemble was carried out for 100 ps at 1 bar and 310 K with the Parrinello–Rahman barostat.^53^ Then, independent production simulations were conducted in replicates for 300 ns. The Particle Mesh Ewald (PME) method was used to handle long-range electrostatic interactions,^54^ while the LINCS algorithm was applied to constrain all covalent bonds. A time step of 2 fs was defined for the simulations.^55^

Trajectory analysis was carried out using the GROMACS tools *gmx rms, gmx rmsf, gmx sasa, gmx gyrate*, and *gmx mindist*. System equilibration was confirmed by monitoring the stability of both the hCOX-1 backbone and ligand RMSD individually, as well as the radius of gyration (Rg), and solvent-accessible surface area (SASA) of the complexes over time. Following equilibration, a more detailed analysis of the interaction profile and local flexibility were performed, focusing specifically on the equilibrated phase of the simulation.^56^

The number of protein–ligand contacts and the minimum distances between these groups were calculated using a 5 Å threshold.^57^ Replicate simulations were concatenated and individual frames were extracted from the corresponding GROMACS topology and trajectory files using the MDAnalysis package,^58^ with each frame saved as a separate PDB file. Individual frames were analyzed using the Protein–Ligand Interaction Profiler (PLIP) library to identify noncovalent protein–ligand interactions, including hydrogen bonds, hydrophobic contacts, π-stacking, cation−π, salt bridges, and halogen bonds. Detailed interaction reports generated by PLIP^59^ were then integrated into a proprietary Python framework using NumPy^60^ and pandas libraries,^61^ which computed the occupancy for each interaction type for relevant hCOX-1 residues. Binding free energy calculations (ΔG_binding)_ were performed using the *g_mmpbsa* software,^62^ which implements the Molecular Mechanics Poisson–Boltzmann Surface Area (MM/PBSA) method. The decomposed molecular mechanics potential energies (E_MM_) and the polar (E_GB_) and nonpolar SASA-based solvation (E_SA_) terms were computed for each simulation snapshot.

Finally, to assess local flexibility, i.e., per residue, and identify hCOX-1 regions influenced by ligand binding, root-mean-square fluctuation (RMSF) analyses were performed on both ligand-bound and apo hCOX-1 simulations. The N- and C-terminal regions were excluded from the RMSF analysis, as terminal residues typically exhibit enhanced conformational freedom that can dominate RMSF profiles, thereby masking more subtle and functionally relevant dynamics of the structured protein core. Accordingly, terminal residues were omitted to enable clearer visualization and more reliable comparison of representative protein flexibility.^63^

Data visualization and descriptive analyses were performed using Python scripts that integrated the *g_mmpbsa* workflow,^62^ along with functions from the *matplotlib*, *seaborn*, *pandas*, and *numpy* libraries.^61^

## 3 Results and Discussion

### 3.1 Synthesis of Piperazine Thioureas

A series of thiourea compounds were synthesized as summarized in Scheme 1. Piperazine thioureas **3a–p** were obtained via the reaction of an excess of phenyl isothiocyanate (**1a**), benzyl isothiocyanate (**1b**), phenethyl isothiocyanate (**1c**), or 3,4,5-trimethoxyphenyl isothiocyanate (**1d**) with the appropriate *N*-monosubstituted piperazine **2** in dichloromethane at room temperature. The use of an excess of isothiocyanate in these reactions was strategically chosen due to its higher solubility in nonpolar solvents such as hexane, in contrast to the more polar thiourea products. This difference in solubility made it possible to purify the crude products simply by washing with hexane, thus eliminating the need for chromatographic purification and contributing to the practicality and cost-effectiveness of the method.

**Scheme 1:**
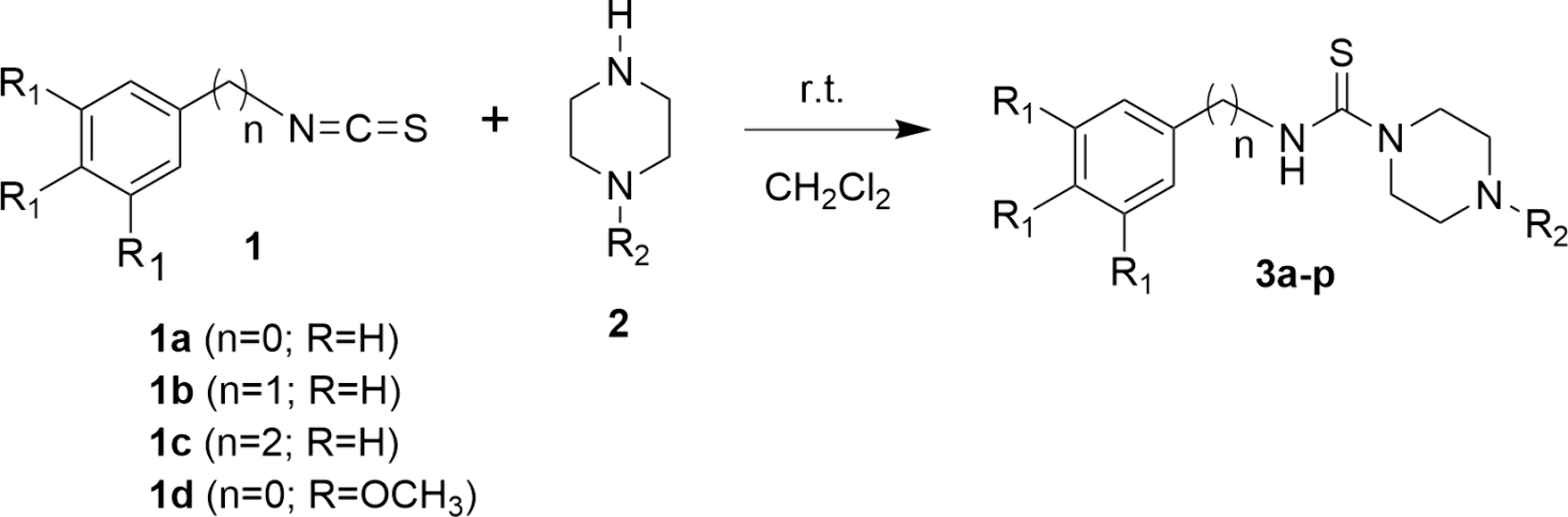
Synthesis of Piperazine Thioureas 3a–p

All reactions proceeded smoothly, affording the corresponding thiourea derivatives in good yields (typically above 90%; Table 1), confirming the efficiency and reproducibility of the adopted synthetic route. This methodology stands out for its operational simplicity, mild reaction conditions, and economic feasibility, as it requires no catalyst, heating, or complex purification steps. Despite its simplicity, the approach enables access to a structurally diverse library of thiourea derivatives through straightforward modification of either the isothiocyanate or piperazine precursors. Such versatility is highly advantageous in Medicinal Chemistry, as it facilitates the rapid generation of new molecular prototypes with distinct physicochemical and pharmacophoric features, thereby accelerating the identification of promising drug candidates.

**Table 1:** Reaction Yields for Thioureas 3a–p

Thiourea^a^	Yield (%)^b^	R_1_ ^c^	R_2_ ^c^	n^c^
**3a**	90	H	Ph	0
**3b**	95	H	Ph	1
**3c**	92	H	Ph	2
**3d**	95	OMe	Ph	0
**3e**	99	H	4-OH-Ph	0
**3f**	97	H	4-OH-Ph	1
**3g**	99	H	4-OH-Ph	2
**3h**	99	OMe	4-OH-Ph	0
**3i**	99	H	2-Cl-Ph	0
**3j**	99	H	2-Cl-Ph	1
**3k**	97	H	2-Cl-Ph	2
**3l**	99	OMe	2-Cl-Ph	0
**3m**	99	H	CH(Ph)_2_	0
**3n**	99	H	CH(Ph)_2_	1
**3o**	99	H	CH(Ph)_2_	2
**3p**	99	OMe	CH(Ph)_2_	0

a Products were characterized by physical and spectroscopic methods.

b Isolated yield.

c Substituent groups (R_1_ and R_2_) and the variable methylene chain length (n) correspond to the structure in Scheme 1.

### 3.2 Biological Assays

#### 3.2.1 Inhibition of AA-Induced Platelet Aggregation

An evaluation of the antiplatelet activity of piperazine thiourea derivatives was initially conducted using an induced platelet aggregation assay with arachidonic acid (AA) and human platelet-rich plasma (PRP) (Figure 2). The results demonstrated an inhibitory effect on maximal platelet aggregation (at a concentration of 100 μM) for four of the tested molecules (**3a**, **3g**, **3j**, and **3p**) that showed inhibition of human platelet aggregation at approximately 91.9 ± 6.7%, 93.1 ± 10.1%, 91.7 ± 0.5% and 94.5 ± 1.8%, respectively (Table 2). These results were statistically comparable to aspirin (ASA) at 100 μM (89.8 ± 0.7%), the most prescribed drug for cardiovascular disease.

**Figure 2:**
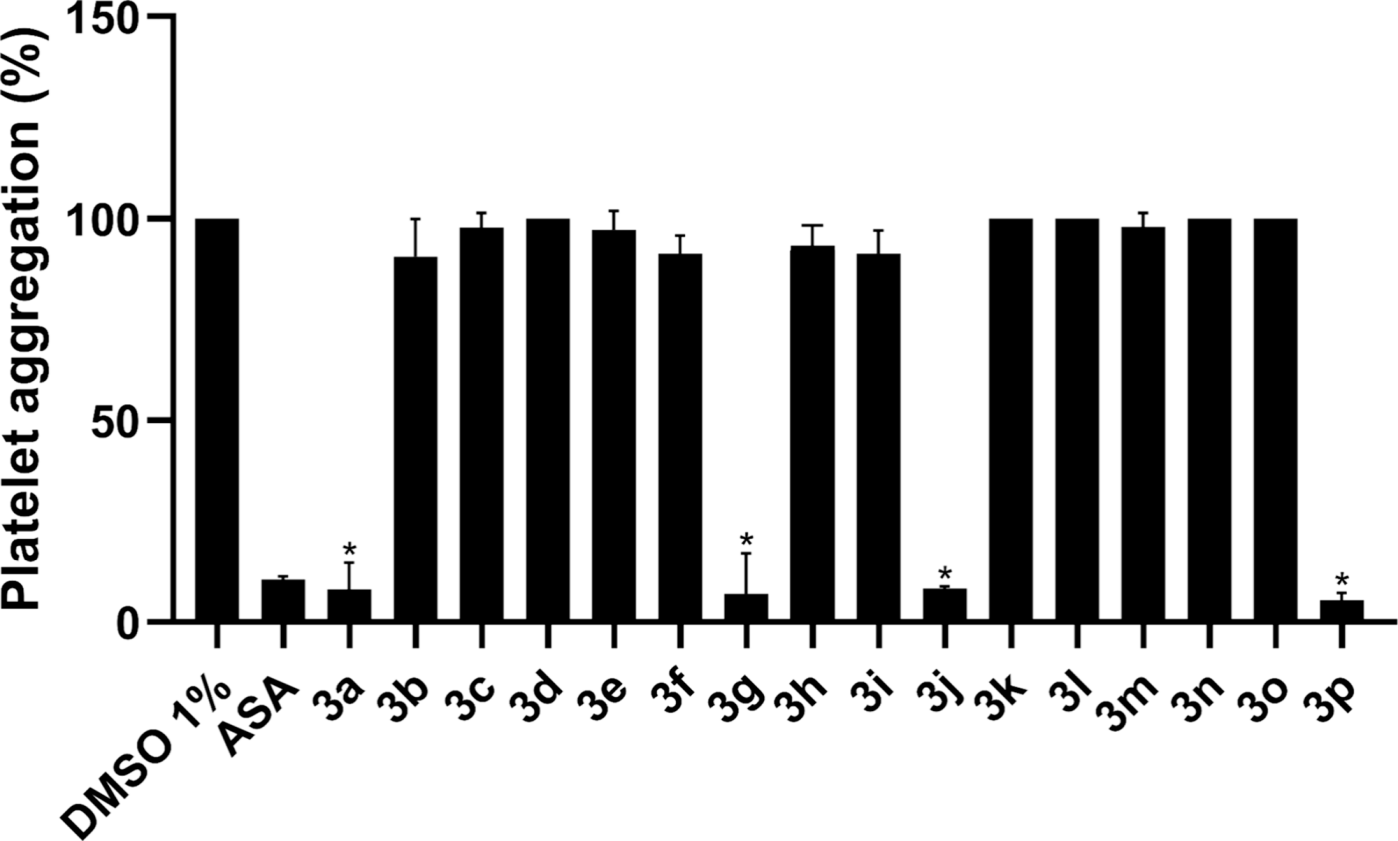
Antiplatelet effects of piperazine thiourea derivatives at a concentration of 100 μM on platelet aggregation induced by arachidonic acid (AA) (500 μM). Acetylsalicylic acid (ASA) was used as positive control, and DMSO (1%) as a negative control. *p ≤ 0.05 versus negative control (one-way ANOVA, Tukey’s test).

**Table 2:** Antiplatelet Profile of Piperazine Thiourea Derivatives (100 μM) on Platelet Aggregation Induced by Arachidonic Acid (AA) (500 μM)^a^

Compound	Aggregation (%)	Inhibition (%)
**3a**	8.9 ± 6.7	91.9 ± 6.7
**3g**	6.9 ± 10.1	93.1 ± 10.1
**3j**	8.3 ± 0.5	91.7 ± 0.5
**3p**	5.5 ± 1.8	94.5 ± 1.8
**DMSO 1%**	100.0 ± 0.0	0.0 ± 0.0
**ASA**	10.2 ± 0.7	89.8 ± 0.7

a Acetylsalicylic acid (ASA) was used as a positive control, and DMSO (1%) as a negative control. Results are expressed as mean ± standard deviation (SD) (*n* = 3). *p ≤ 0.05 versus negative control (one-way ANOVA, Tukey’s test).

The mechanism of action of ASA involves the inhibition of the arachidonic acid (AA) pathway, wherein the ASA molecule irreversibly acetylates cyclooxygenase (COX-1), leading to the permanent inactivation of the enzyme. Inactivated COX-1 is unable to catalyze the conversion of AA into prostaglandin H2 (PGH_2_), the precursor of prostaglandins, prostacyclins, and primarily thromboxane A2 (TxA_2_), which stimulates platelet activation and aggregation.^64^ These findings, obtained from *in vitro* platelet aggregation assays, are consistent with our previous studies on earlier urea derivatives (*N*,*N*’-disubstituted ureas), which also demonstrated antiplatelet effects associated with COX-1 inhibition.^30^ Additionally, the compounds were evaluated for their concentration required to inhibit 50% of platelet aggregation induced by the AA agonist (IC_50_) (Figure 3). The results revealed that **3p** (74.5 ± 4.8 μM) exhibited the highest potency, followed by **3a** (80.7 ± 11.0 μM), **3g** (83.1 ± 14.7 μM) and **3j** (88.0 ± 9.9 μM).

**Figure 3:**
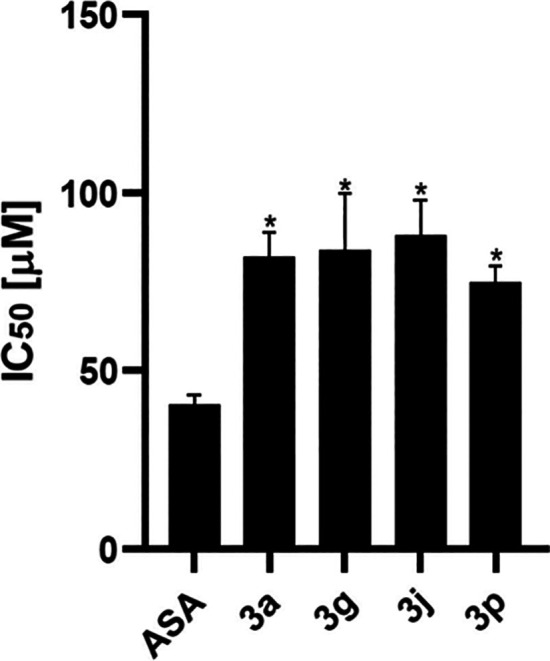
Concentration required to inhibit 50% of arachidonic acid (AA)-induced platelet aggregation (IC_50_) for piperazine thiourea derivatives compared to acetylsalicylic acid (ASA). Data are expressed as the mean ± standard deviation (SD) (*n* = 3). IC_50_ values (μM) were determined for compounds 3a (80.7 ± 11.0), 3g (83.1 ± 14.7), 3j (88.0 ± 9.9), 3p (74.5 ± 4.8), and ASA (36.3 ± 4.2). *p ≤ 0.05 versus positive control (one-way ANOVA, Tukey’s test)..

#### 3.2.2 *In Vitro* Anticoagulant Assays

The coagulation assays performed, prothrombin time (PT) and activated partial thromboplastin time (aPTT), revealed no anticoagulant activity and no statistical significance (*p* ≥ 0.05) for the piperazine thiourea derivatives, either in the extrinsic or intrinsic pathway. There was no increase in the ratio between the coagulation time of the derivatives and the control (DMSO 1%) (Figure 4). These results provide evidence of the hemocompatibility of the compounds and their action specifically in primary hemostasis without a dual activity in both primary and secondary hemostasis, a mechanism which can cause severe bleeding disorders as reported in the literature.^65^

**Figure 4:**
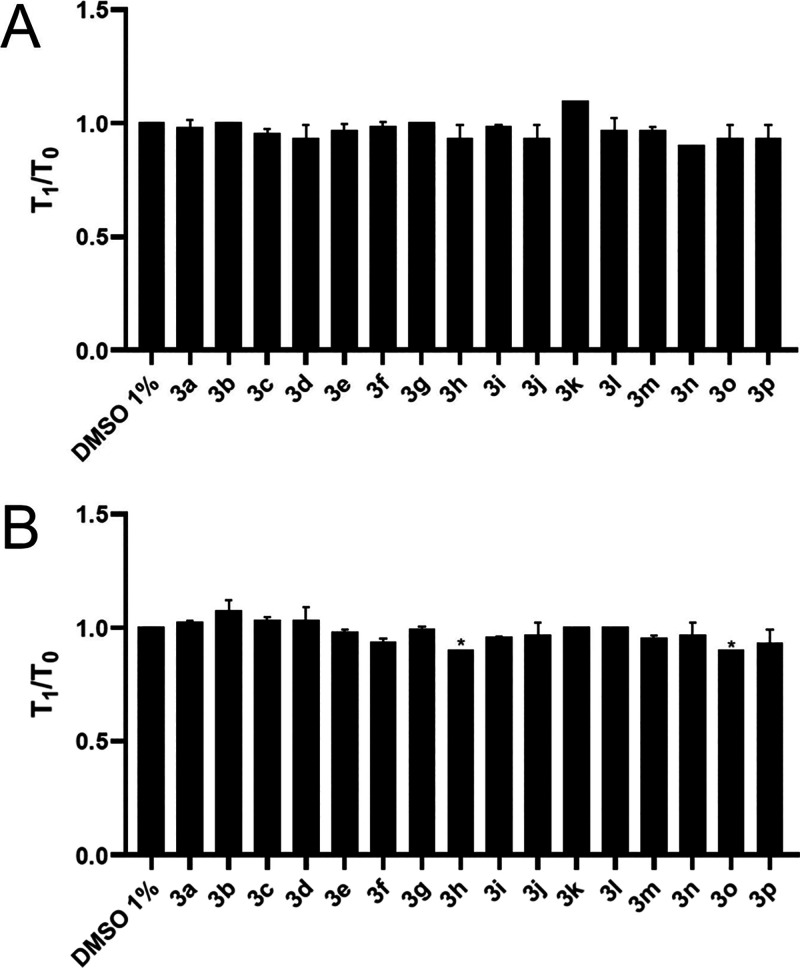
Evaluation of piperazine thiourea derivatives on the in vitro coagulation of human plasma, assessed by (A) prothrombin time (PT) and (B) activated partial thromboplastin time (aPTT). All compounds were tested at a concentration of 100 μM, with 1% DMSO used as a negative control. T_1_ /T_0_ represents the ratio of test time to control time (in seconds). The results are expressed as the mean ± standard deviation (SD) (*n* = 3). *p ≤ 0.05 versus negative control (one-way ANOVA, Tukey’s test).

#### 3.2.3 Hemolytic Activity

The hemolysis assay was conducted to evaluate whether the piperazine thiourea derivatives interact with erythrocyte membranes, potentially causing premature cell lysis and hemoglobin release. According to Fischer et al. (2003),^66^ hemolysis values exceeding 10% are indicative of hemolytic activity. None of the compounds at 100 μM exhibited a hemolytic profile above this threshold, with hemolysis values ranging from 0.00 ± 0.00% to 9.9 ± 0.1%. Although the values obtained for the compound **3d** were close to 10% (9.9 ± 0.1%), these results were significantly lower than the positive control, Triton X-100 (100%) (Figure 5), indicating that the compounds are hemocompatible, safe, and present a low risk of toxicity.

**Figure 5:**
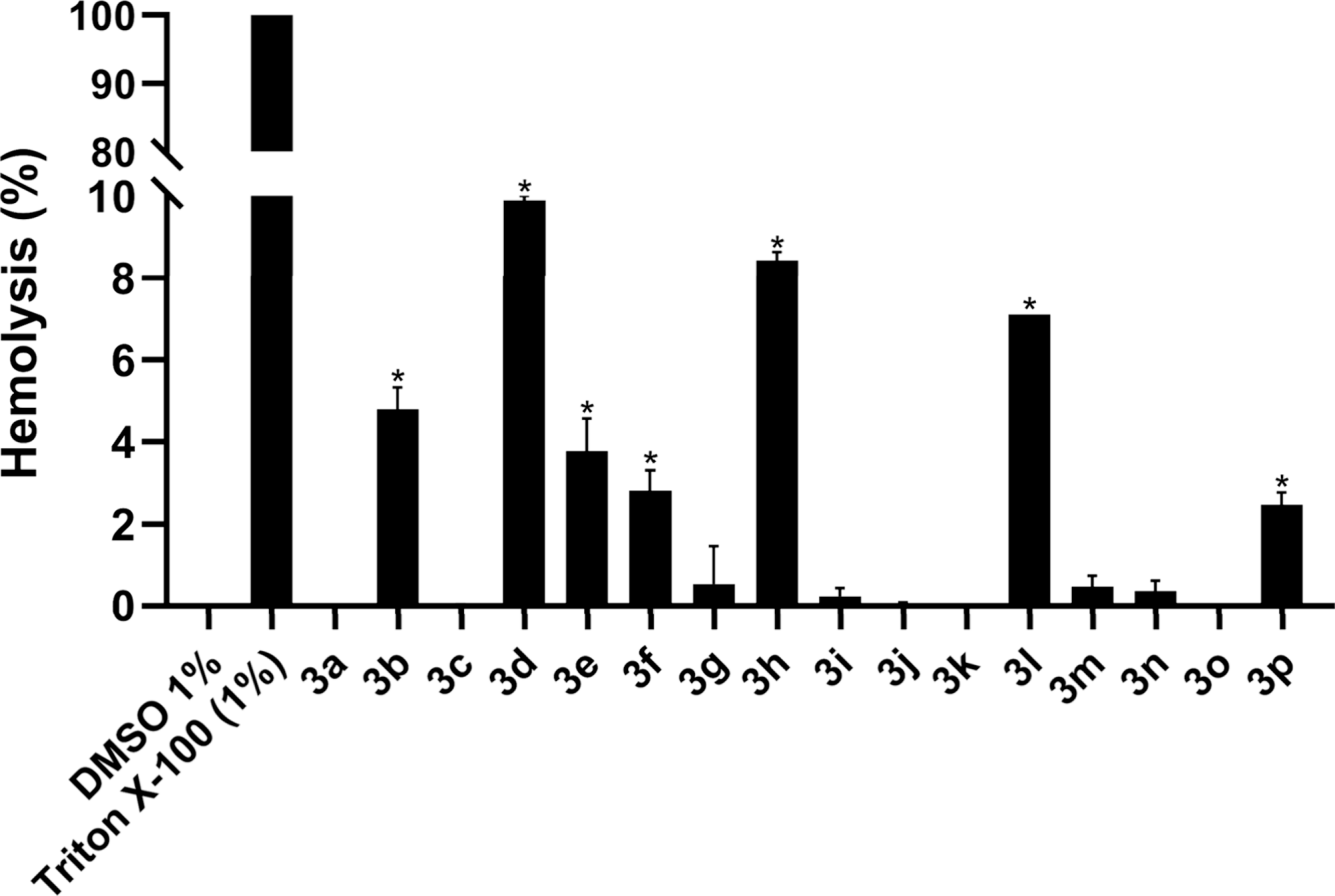
Hemocompatibility assessment of piperazine thioureas derivatives (100 μM) using the hemolysis assay after 3 h of incubation. Values below 10% are considered nonhemolytic. DMSO 1% is the negative control and Triton X-100 1% is the positive control. The data were presented as mean ± standard deviation (SD) (*n* = 3). *p ≤ 0.05 versus negative control (one-way ANOVA, Tukey’s test).

### 3.3 *In Silico* Pharmacokinetics and Toxicological Assessment

Inadequate pharmacokinetic and toxicological properties are among the leading causes of failure in drug development.^67^ QSAR models, such as the ADMET Predictor, have been widely utilized for decades to establish correlations between chemical structure and biological activity, owing to their recognized ability to predict the pharmacokinetic and toxicological properties of novel molecules.^68^ Consequently, they have become essential tools for guiding decision-making in the early stages of drug development, particularly in identifying compounds with a higher likelihood of achieving desired pharmacological properties *in vivo*—achieving effective concentrations at therapeutic targets with low toxicity.^69^ This approach also aligns with the principles of the 3Rs (replacement, refinement, and reduction) by adopting alternative methods to reduce animal testing in preclinical studies, as recommended by the Organization for Economic Cooperation and Development (OECD).^70^

Therefore, we performed a comprehensive *in silico* characterization of the ADMET profiles of the active piperazine thioureas **3a**, **3g**, **3j**, and **3p**, from which the results are presented in Table 3. The ADMET Predictor software uses a risk scoring system providing a quantitative evaluation of pharmacokinetic and toxicological risks. Each risk model was calibrated with specific thresholds based on approximately 90% of the empirical distribution of commercial drugs in the WDI database,^32^ as follows: ADMET Risk ≤ 7, Absn Risk ≤ 4, CYP Risk ≤ 2, Tox Risk ≤ 2, Mut Risk ≤ 1, and Mutx Risk ≤ 1.^57^

**Table 3:** Predicted Pharmacokinetic and Toxicological Profiles of Active Thioureas

**Compound**	**ADMET Risk**	**Absn Risk**	**CYP Risk**	**Tox Risk**	**Mut Risk**	**Mutx Risk**	**Lipinski’s violations^a^**
**3a**	1.29 (Xm^–^; 2D6)	0	0.79 (2D6)	0,5 (Xm^–^)	0	0	0
**3g**	0.99 (3A4)	0	0.99 (3A4)	0	0	0	0
**3j**	2.11 (Sw; fu; 2D6; 3A4; CL)	0.27 (Sw)	1.365 (2D6; 3A4; CL)	0	0	0	0
**3p**	5.39 (Size; RotB; Kow; fu; Xm^–^; 3A4; CL)	2.02 (Size; RotB; Kow)	2 (3A4; CL)	0.5 (Xm^–^)	0	0	0
*Cut-off* ^*1*^	≤7	≤4	≤2	≤2	≤1	≤1	<2

a The following codes, shown in parentheses, correspond to potential pharmacokinetic and toxicological issues a compound may have Kow = lipophilicity; Sw = water solubility; Size = molecular weight; RotB = rotatable bonds; fu = unbound fraction in human plasma; Xm = carcinogenicity in mice; 3A4 = high clearance by CYP3A4; 2D6 = high clearance by CYP2D6; CL = high clearance in microsomal model; Superscripted minus represent predictions outside the model’s applicability domain. ^1^Limit based on an empirical distribution calculated from commercially available drugs in the WDI subset.

The overall ADMET evaluation, based on the ADMET risk model, revealed that the antiplatelet thioureas received penalty scores ranging from 0.99 to 5.39 (Table 3), all of which are well below the rejection threshold of 7 penalties. The individual evaluations additionally demonstrate that all compounds display favorable profiles across the risk categories but raised some risk flags related to potential ADMET issues, which are properly discussed below.

Although integrated into the overall ADMET risk model, ADMET Predictor also offers separate evaluations for the Vd (volume of distribution) and fu (fraction unbound) parameters, which are central for assessing the distribution and excretion profiles. Among the active thioureas, the Vd risk model did not indicate any violations. **3j** and **3p**, in turn, exhibited fu values outside the optimal range, raising potential issues related to distribution or excretion. Despite this, both compounds exhibited fu parameter values still within the model’s soft threshold. This indicates that although the compound slightly deviates from the algorithm’s ideal limit, its predicted risk remains within a narrow margin of the defined cutoff, incurring only partial penalties (penalty <1) (Simulations Plus, Inc., Lancaster, CA, USA).

Only compounds **3j** and **3p** raised alerts regarding possible absorption issues, resulting in penalties of 0.27 and 2.02, respectively, for this risk category. Compound **3j** triggered the water solubility (Sw) alert, while **3p** raised alerts for size (Size), number of rotatable bonds (RotB), and lipophilicity (Kow). Despite these alerts, they remain below the absorption risk limit, i.e., Absn risk ≤4. Additionally, none of the active thioureas violated Lipinski’s rule of five. Lipinski’s rule of five states that a compound is likely to have poor oral absorption or permeability when ≥2 rules are violated, a criterion that applies to most commercial oral drugs.^71^ Given the respective Absn risk values and number of Lipinski rule violations, the antiplatelet thioureas exhibit drug-like properties, indicating they might have satisfactory oral bioavailability.

The metabolism assessment reveals that all active thiourea were flagged for potentially elevated intrinsic clearance levels, particularly through CYP2D6, CYP3A4 enzymes or hepatic microsomal pathways (CL). Intrinsic clearance refers to the liver’s ability to eliminate a specific drug from circulation without restrictions due to blood flow.^72^ Extensive metabolism, driven by high clearance levels, is known to reduce the half-life and bioavailability of drugs.^73^ Despite these alerts, all compounds displayed a favorable CYP risk profile, with scores comparable to those of most commercial drugs (CYP risk ≤ 2). This suggests that their metabolic clearance is unlikely to compromise their effectiveness (Simulations Plus, Inc., Lancaster, CA, USA).

Given the importance of the mutagenicity end point in ensuring drug safety, ADMET Predictor includes a dedicated mutagenicity model, which is also factored into the overall TOX risk evaluation. Compounds that do not raise alerts in this model are likely to exhibit minimal genotoxic risk, contributing to an improved overall safety profile. This favorable prediction was observed for all antiplatelet thioureas (Table 3).

The toxicity assessment also indicated that no hepatotoxicity, acute toxicity nor cardiotoxicity alerts were raised, suggesting a low likelihood of these adverse effects across all active thioureas. Additionally, **3g** and **3j** incurred no toxicity penalties, achieving a TOX risk score of 0, thus, presenting an optimal safety profile. Compounds **3a** and **3p**, in turn, received a toxicity penalty of 0.5, primarily attributed to out-of-scope carcinogenicity in mice (Xm^–^). The out-of-scope condition indicates that the evaluated compounds fall outside the chemical space represented in the model’s training set. Consequently, the predictions for these compounds may lack reliability and should be interpreted with caution. For this reason, these compounds were assigned only half of a complete penalty, i.e., a penalty of 0.5 (Simulations Plus, Inc., Lancaster, CA, USA). Our findings therefore suggested that all antiplatelet thioureas remain within the established toxicity cutoff limit (TOX risk ≤ 2), indicating that their safety profiles are comparable to those of most commercial drugs (Simulations Plus, Inc., Lancaster).

In addition to showing promising *in vitro* antiplatelet aggregation activity and drug-like properties (Table 3), the investigated thiourea derivatives appear to have an adequate safety profile, as indicated by the ADMET Predictor analysis.

### 3.4 Molecular Docking

Molecular docking is a well-established method for predicting the preferred conformation of a ligand within the active site of a target protein, thus elucidating potential binding modes and affinities. The accuracy of molecular docking simulation results depends significantly on the quality of the selected target protein structure and the docking algorithm’s ability to predict interactions within the site of interest. To this end, docking algorithms are previously subjected to rigorous evaluation using a test set composed of numerous protein–ligand complexes available in the PDB, from which the poses predicted are compared with the experimental reference conformations.^74^

Despite the previous validation process, a common practice is to conduct an additional site-directed validation through redocking. This procedure involves removing a known ligand from the active site of the target protein and verifying whether the docking algorithm can approximate the experimental pose.^48^ To confirm this behavior, structural overlapping metrics, such as RMSD, are used. In redocking, the RMSD is calculated based on the deviation between the corresponding atoms of the predicted ligand pose and the experimental reference conformation. In addition to RMSD analysis, it is also important that the method reproduces experimental binding modes, which is usually verified through visual inspection.^75^

There are no determined structures of hCOX-1 complexed with ligands to date, and thus, the validation of this site could not be directly conducted. Accordingly, the apo structure of hCOX-1 (PDB ID: 6Y3C)^37^ represents the only experimentally resolved structure of human COX-1,^38^ as shown in Figure S1.

Nonetheless, hCOX-1 has significant evolutionary conservation in the cyclooxygenase site and a considerably high sequence identity with oCOX-1, i.e., 92%.^37^ This close homology is reflected in the pronounced structural similarity between the apo hCOX-1 structure (PDB ID: 6Y3C)^37^ and the oCOX-1 structure cocrystallized with the selective inhibitor mofezolac (PDB ID: 5WBE).^38^ Beyond global structural similarity, Figure 6 highlights the complete conservation of the cyclooxygenase active site, revealing identical amino-acid composition and near-perfect three-dimensional superposition of the catalytic channel. Together, these features demonstrate that both the overall fold and the precise spatial arrangement of catalytically relevant residues are preserved across species.

**Figure 6:**
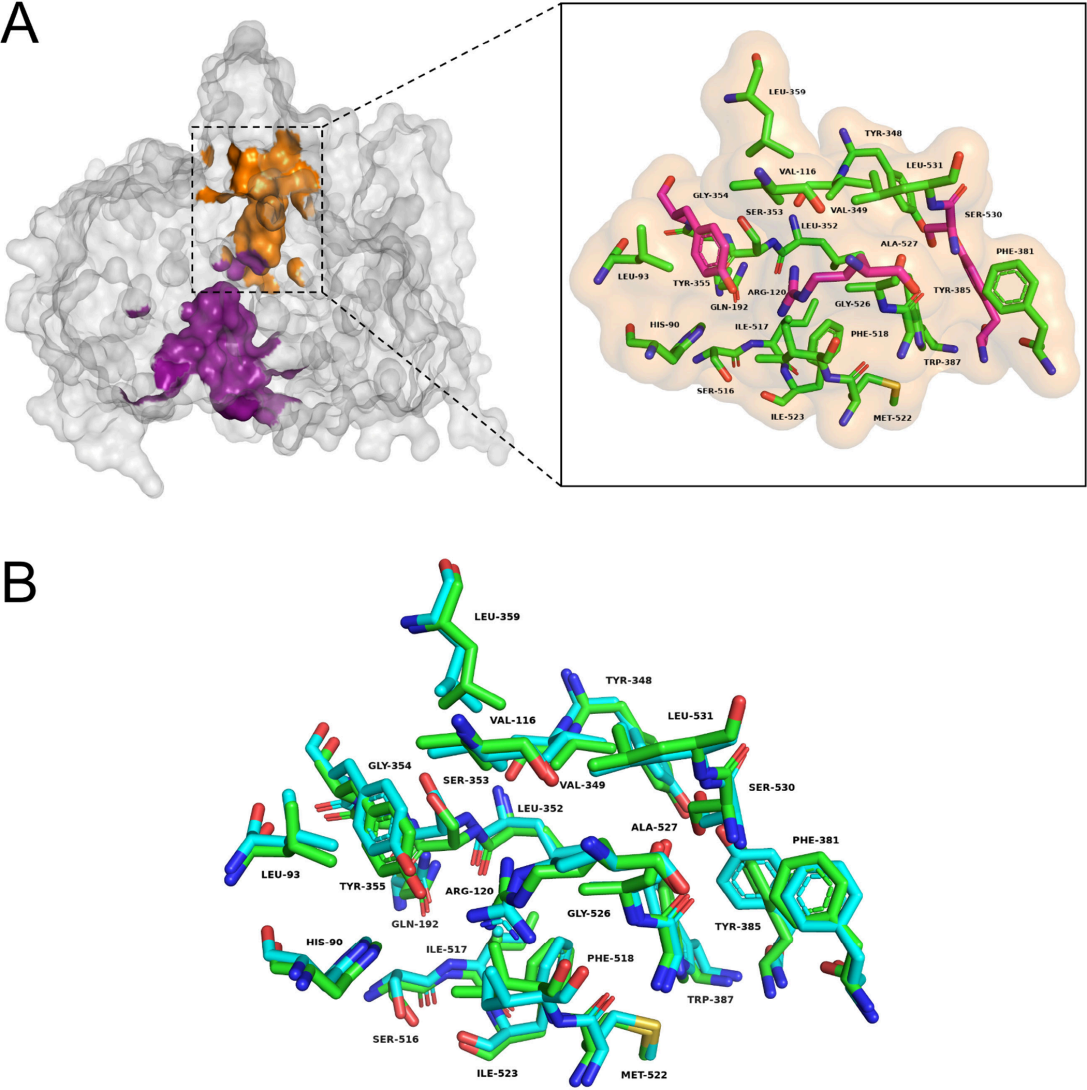
Structural organization and active-site conservation of cyclooxygenase-1 (COX-1) in ovine and human enzymes. (A) Three-dimensional representation of COX-1 highlighting the spatial organization of its two functionally coupled catalytic regions: the cyclooxygenase catalytic site (orange) and the peroxidase site (purple). Key residues involved in substrate binding, catalytic initiation, and site-specific inhibition are highlighted in pink. (B) Superposition of the cyclooxygenase catalytic site showing identical amino-acid composition and near-perfect spatial overlap between human COX-1 (green) and ovine COX-1 (cyan). Amino acid residues are represented using their standard three-letter codes, as follows: LEU: Leucine, HIS: Histidine, ARG: Arginine, VAL: Valine, TYR: Tyrosine, GLN: Glutamine, ILE: Isoleucine, SER: Serine, MET: Methionine, PHE: Phenylalanine, ALA: Alanine, TRP: Tryptophan, and GLY: Glycine.

As shown in Figure 6, the cyclooxygenase active site, which is highlighted in orange, adopts a long, narrow, and predominantly hydrophobic channel that serves as the primary binding cavity for the substrate arachidonic acid and nonsteroidal anti-inflammatory drugs like mofezolac. Previous crystallographic studies have demonstrated that this channel is defined by residues essential for substrate recognition and catalysis, mainly Arg120, Tyr355, Tyr385, and Ser530, which are strictly conserved between human and oCOX-1 and exhibit near-identical spatial organization within the binding pocket. Particularly, Arg120 and Tyr355, positioned at the entrance of the channel, stabilize ligand binding through electrostatic and hydrogen-bond interactions with the carboxylate group of arachidonic acid. Tyr385 and Ser530, in turn, are located near the bend of the characteristic L-shaped cavity and are respectively involved in catalytic initiation and inhibitor binding.^76,77^

In this context, the validation through redocking of oCOX1 (5WBE)^40^ could serve as a relevant indicator of the accuracy of the Gold algorithm in the cyclooxygenase site of hCOX1. The representative redocking conformation presented an RMSD value of 0.74 Å relative to the crystalline pose of mofezolac, suggesting a suitable match between the poses.^78^ This conclusion was further supported by visual inspection (Figure 7), which reaffirmed the similarity between the poses and allowed analysis of the formed interactions.^75^ Similar to the binding modes described in the 5WBE structure,^40^ the redocking suggests that the carboxylate portion of mofezolac forms a salt bridge with Arg120, as well as hydrogen bonds with Arg120 and Tyr355. This reaffirms the predictive capability of the algorithm in the cyclooxygenase site of ovine COX1, which could be extrapolated to the same site in human COX-1.^43,78^

**Figure 7:**
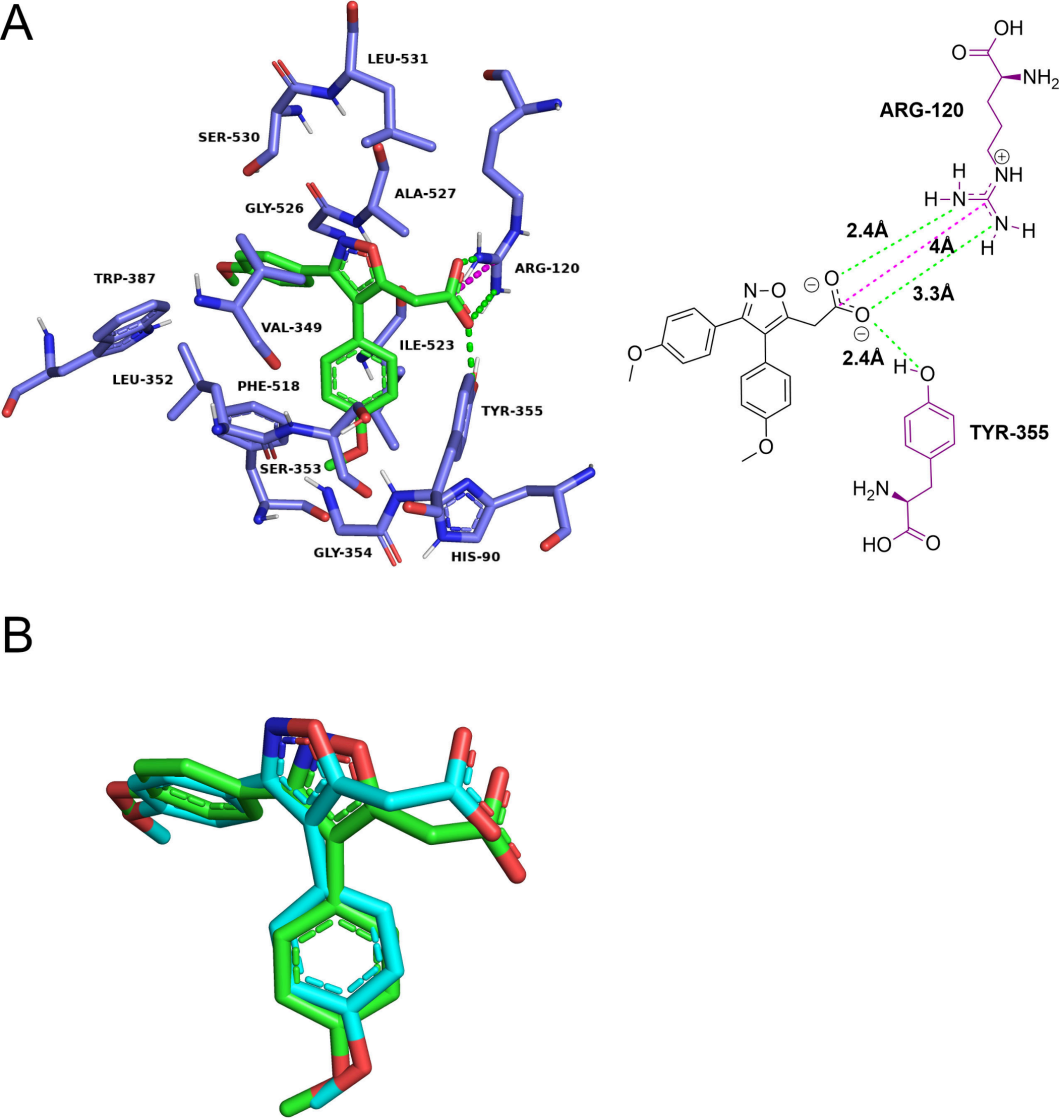
Redocking simulations conducted to validate the Gold algorithm in the reference site of oCOX-1 receptor. (A) Experimentally determined binding modes of the COX-1–mofezolac complex, as recorded in the PDB structure 5WBE. Mofezolac is represented by green sticks, color-coded by atom type. The residues interacting with the ligand are shown as purple sticks, color-coded by atom type, and labeled according to the corresponding amino acid. Hydrogen bonds are depicted as green dashed lines, while salt bridges are represented by pink dashed lines. Residues involved in hydrophobic contacts with the inhibitor are also displayed. A complementary 2D interaction diagram is also provided to facilitate visualization of protein–ligand interactions, with all interaction distances displayed. (B) Redocking of the COX-1-mofezolac complex (5WBE). The experimental pose is represented by green sticks, while the docked pose is shown as cyan sticks, both color-coded by atom type. Amino acid residues are represented using their standard three-letter codes, as follows: ALA: Alanine, ARG: Arginine, GLY: Glycine, HIS: Histidine, ILE: Isoleucine, LEU: Leucine, PHE: Phenylalanine, SER: Serine, TRP: Tryptophan, TYR: Tyrosine, and VAL: Valine.

To further strengthen the validation of the docking protocol, an additional docking simulation was performed in which mofezolac was docked into the cyclooxygenase active site of hCOX-1 (PDB ID: 6Y3C). The predicted pose of mofezolac in hCOX-1 (Figure S2) closely reproduces the binding mode observed in the oCOX-1 crystal structure, preserving the orientation within the cyclooxygenase channel and the key interactions with conserved active-site residues, including anchoring contacts at the channel entrance involving Arg120 and Tyr355. Notably, the interaction between mofezolac and Tyr355 is characterized by a (N···O) donor–acceptor heavy-atom distance of approximately 3.6 Å, which is consistent with a weak hydrogen bond. Although such interactions are associated with lower energetic contributions, they remain structurally and functionally relevant in protein–ligand recognition and can contribute to stabilizing ligand binding within the active site.^79,80^ Comparative analysis of the docking poses in oCOX-1 (Figure 8) and hCOX-1 (Figure S2) further reveals a highly similar ligand orientation and interaction pattern, consistent with the strict conservation of the cyclooxygenase binding pocket between these species. Therefore, our findings demonstrate the predictive capability of the docking algorithm used in the active site of the study’s target protein, i.e., COX-1, indicating the reliability of the results obtained through this method.^43,48^

**Figure 8:**
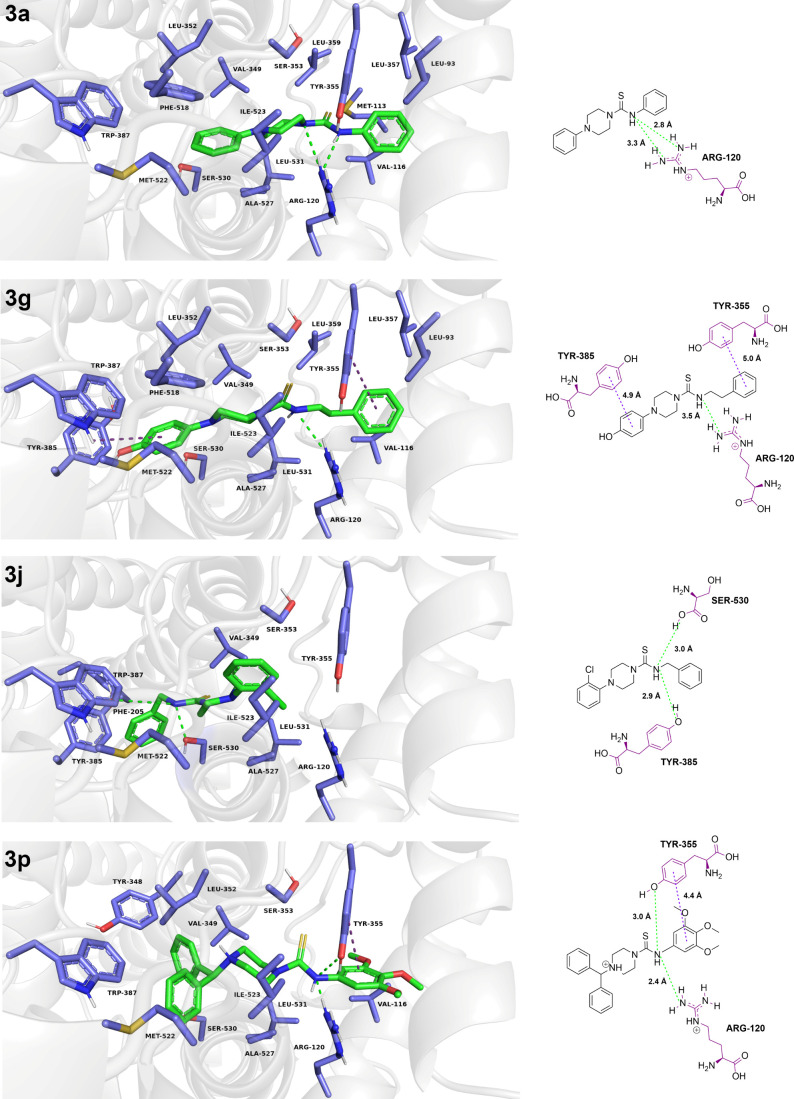
Docking of active thioureas **3a**, **3g**, **3j**, and **3p** on the COX-1 receptor. The ligands are represented by green sticks and colored by atom type. The residues involved in interactions with the ligand are shown as purple sticks, colored by atom type, and labeled according to the corresponding amino acid. Hydrogen bonds are represented by dashed green lines, salt bridges by dashed pink lines, π–π interactions by dashed purple lines, and cation−π interactions by dashed orange lines. Residues involved in hydrophobic contacts with the inhibitor are also shown. Complementary 2D interaction diagrams are also provided to facilitate visualization of protein–ligand interactions, with all interaction distances displayed. Amino acid residues are represented using their standard three-letter codes, as follows: ALA: Alanine, ARG: Arginine, ILE: Isoleucine, LEU: Leucine, MET: Methionine, PHE: Phenylalanine, SER: Serine, TRP: Tryptophan, TYR: Tyrosine, and VAL: Valine.

After verifying the predictive capability of the molecular docking algorithm, we elucidated the binding modes of the antiaggregant thiourea derivatives in the active site of their potential receptor, i.e., COX-1 ^13^. The molecular docking analysis on the COX-1 receptor (Figure 8) suggested that the compounds **3a**, **3g**, **3j**, and **3p** interacted with key residues involved in receptor inhibition, i.e., Arg120 and/or Tyr355.^40^ These compounds consistently form hydrogen bonds with Arg120 and Tyr355 through the amine group of their thiourea moiety (Figure 8). Notably, **3p** exhibited binding modes similar to those of the selective COX-1 inhibitors mofezolac (5WBE) and flurbiprofen (3N8Z). The primary distinction is the absence of a salt bridge with Arg120, an interaction observed in the reference inhibitors but absent in the evaluated thioureas, due to the lack of a negatively charged moiety.^81^

The docking analysis additionally reveals a conserved binding profile for part of the series, particularly with respect to the thiourea moiety, which becomes evident upon superposition of the predicted poses within the COX-1 binding cavity (Figure S3). Compounds **3a**, **3g**, and **3p** exhibit a similar spatial orientation of this core, supporting the presence of a common anchoring mode within the active site. In contrast, compound **3j** deviates from this trend, adopting a distinct binding orientation and interaction profile.

For compounds **3a**, **3g**, and **3p**, the thiourea-piperazine moiety is consistently oriented toward the polar region of the binding pocket, where it establishes stabilizing hydrogen-bonding interactions. In these complexes, the thiourea nitrogen atoms recurrently interact with Arg120 (Figure 8), a residue recognized as critical for ligand anchoring in the cyclooxygenase active site.^40^ Notably, in compound **3p**, the thiourea nitrogen forms an additional hydrogen bond with Tyr355, which may contribute to enhanced stabilization of the protein–ligand complex. This interaction could partly explain the higher biological activity observed for this compound and further supporting the role of the thiourea group as a relevant pharmacophoric motif for COX-1 binding and inhibition.

In contrast, compound **3j** exhibits an alternative binding pattern, in which the thiourea group is displaced from the polar region of the pocket (Figure 8). As a result, the characteristic hydrogen-bonding interaction with Arg120 is not preserved, and the thiourea moiety does not participate in the same interaction network observed for compounds **3a**, **3g**, and **3p**. This divergence highlights the sensitivity of ligand orientation to substituent-dependent steric and electronic effects, likely associated with the presence of a chlorinated moiety absent in the other thiourea derivatives. The chlorine atom, due to its larger van der Waals radius and increased polarizability, may impose steric constraints that subtly reshape the binding pose. In addition, its hydrophobic character is well suited to the largely nonpolar environment of the deeper region of the COX-1 cyclooxygenase channel, which is enriched in aromatic residues and aliphatic side chains. Together, these steric and hydrophobic contributions may favor alternative ligand orientations that optimize van der Waals and hydrophobic contacts, thereby modulating the overall binding mode.^81^ AsQ1: Canal de entrada - bolsão hidrofóbico. a result, the thiourea-piperazine core adopts a distinct orientation within the binding cavity, positioning the thiourea nitrogen toward residues located deeper in the cyclooxygenase channel, i.e., Tyr385 and Ser530.

Tyr385 is a key residue situated near the apex of the cyclooxygenase active site and is directly involved in the hydrogen abstraction step that initiates arachidonic acid oxygenation. Ser530 is well-known as the acetylation site of aspirin, whose irreversible modification sterically blocks substrate access to the cyclooxygenase channel. Interactions with these residues have been reported for several nonsteroidal anti-inflammatory drugs, including aspirin, indomethacin, flurbiprofen, and ibuprofen, which bind deep within the cyclooxygenase site.^76,77,81–84^ Accordingly, the binding mode predicted for compound **3j** suggests an alternative interaction pattern that targets the deep catalytic region of COX-1, rather than the Arg120-centered anchoring observed for compounds **3a**, **3g**, and **3p**. Notably, this distinct binding mode remains compatible with effective occupation of the active site and consequent inhibition, consistent with the biological activity determined for compound **3j** in our experimental assay (Figure 3).

We additionally examined the docking poses of inactive compounds to characterize their interaction profiles and binding orientations within the cyclooxygenase site, thereby providing insight into the relationship between compound structure and biological activity across the thiourea–piperazine series.

Among the inactive thioureas (Figure 2), compounds **3c**, **3d**, **3e**, **3f**, and **3i**, fail to establish stable polar anchoring interactions within the cyclooxygenase channel (Figure S4 and Figure S5). These compounds typically adopt misoriented binding poses and exhibit weak or absent interactions with key COX-1 residues, resulting in poor stabilization within the active site. Although occasional π–π stacking or transient contacts are observed, such interactions alone appear insufficient to support effective inhibition.

Compounds **3h**, **3l**, **3m**, and **3n**, in turn, were able to establish stable polar anchoring interactions with the Ser530 residue within the cyclooxygenase binding site (Figure S4 and Figure S5). Nonetheless, they fail to reproduce the broader interaction pattern typically observed for nonsteroidal anti-inflammatory drugs or for the active thiourea derivatives **3a**, **3g**, and **3p**, as previously discussed. Although interaction with Ser530 is a recognized feature of several COX inhibitors, effective inhibition generally depends on the formation of an extensive hydrophobic contact network along the cyclooxygenase channel, involving deep occupation of the channel interior and additional stabilization through interactions with key residues such as Arg120 and Tyr355.^76,77,81–84^ Consequently, the limited interaction network displayed by these compounds likely underlies their lack of measurable biological activity (Figure 2).

Interestingly, compounds **3b**, **3k**, and **3o** adopted binding poses predominantly stabilized by polar interactions near the entrance of the cyclooxygenase channel, particularly involving Arg120 (Figure S4 and Figure S5), a binding pattern commonly reported for several nonsteroidal anti-inflammatory drugs. Their predicted orientations closely resemble those observed for the active thiourea derivatives **3a**, **3g**, and **3p** in this study (Figure 8). Despite these favorable interaction profiles predicted by docking simulations, compounds **3b**, **3k**, and **3o** were classified as inactive in our biological assays (Figure 2). This discrepancy likely reflects intrinsic limitations of docking simulations in reproducing the outcomes of human plasma–based assays. While molecular docking provides valuable insights into binding modes and relative affinities, its predictive scope is confined to direct protein–ligand interactions and thus did not capture pharmacokinetic factors. Consequently, aspects such as plasma protein binding, limited solubility, metabolic instability, and restricted access to the pharmacological target are not reflected in docking simulations, even though they can critically influence compound activity.^85^

Overall, our docking findings provided an in-depth characterization of interaction profiles and binding orientations within the cyclooxygenase site across the piperazine–thiourea series. Nevertheless, no clear correlation was observed between structural variations at R1 and R2 or the linker length (Scheme 1) and the predicted binding modes that could explain the experimental activity across the series.

### 3.5 Structure–Activity Relationship (SAR) Analysis

In order to integrate the biological and *in silico* findings and to rationalize the antiplatelet activity observed across this chemically diverse library, a structure–activity relationship (SAR) analysis was performed. Given the structural heterogeneity of the compounds, SAR trends were evaluated within well-defined subseries rather than through a single global model, allowing the identification of local patterns governing activity.

Within the phenyl-substituted derivatives (**3a**–**3d**), compound **3a** (n = 0) displayed the highest antiplatelet activity, whereas elongation of the linker (**3b**, n = 1; **3c**, n = 2) or introduction of electron-donating substituents on the aromatic ring (**3d**) led to reduced activity. This trend suggests that a shorter and more rigid scaffold favors an optimal positioning of the thiourea moiety within the COX-1 cyclooxygenase channel. Docking simulations support this interpretation, as compound **3a** adopts a binding pose that allows favorable interactions at key anchoring regions, including interactions with Arg120 at the channel entrance. In contrast, compounds **3c** and **3d** did not establish interaction patterns compatible with effective inhibition. In the case of derivative **3b**, despite adopting a binding pose compatible with interaction within the COX-1 site, this interaction did not translate into measurable antiplatelet activity, suggesting that factors not captured by docking simulations, such as solubility, permeability, or effective target engagement under the assay conditions, may limit its functional response.

A distinct SAR profile was observed for the 4-hydroxyphenyl derivatives (**3e**–**3h**), in which **3g** (n = 2) emerged as the most active compound. In this subseries, extension of the linker appears beneficial, likely enabling deeper penetration of the *para*-hydroxyl-phenyl group, which contributes to stabilizing π–π stacking interactions with Tyr385 located in the catalytic region of COX-1. Shorter analogues, such as **3e** and **3h**, showed diminished activity, indicating that insufficient reach or suboptimal spatial orientation compromises effective binding. Although compound **3f** is able to interact with Tyr385, docking analysis indicates that it fails to interact with Arg120 at the channel entrance, likely due to its shorter linker length, which may account for its lower antiplatelet activity compared to **3g**.

Among the 2-chlorophenyl derivatives (**3i**–**3l**), maximal activity was observed for **3j** (n = 1), whereas both shorter (**3i**) and longer (**3k**) analogues were less effective. This behavior reflects a balance between steric constraints imposed by the *ortho*-chloro substituent and hydrophobic complementarity within the cyclooxygenase channel, for which an intermediate linker length appears optimal. Docking analyses corroborate this trend, as **3j** exhibited favorable interactions with key residues involved in COX-1 inhibition. In contrast, compound **3k**, bearing a longer spacer, failed to establish interactions with Tyr385 and Ser530 at the bottom of the binding site, instead forming interactions preferentially at the channel entrance, including contacts with Arg120. Compound **3i**, lacking a linker, did not display an interaction pattern compatible with effective inhibition, as it failed to interact with both Arg120 at the entrance and Tyr385 in the catalytic region. Compound **3l**, which also lacks a linker but bears electron-donating substituents, exhibited a distinct interaction pattern that did not translate into enhanced biological activity.

The benzhydryl derivatives (**3m**–**3p**) constitute the bulkiest and most lipophilic subseries, among which **3p** exhibited the highest overall potency. According to docking results, the combination of a benzhydryl group with a 3,4,5-trimethoxyphenyl moiety maximizes hydrophobic interactions within the deeper regions of the COX-1 binding pocket while simultaneously enabling polar interactions at the channel entrance. Compounds **3m** and **3n** displayed alternative interaction patterns that minimized favorable contacts at the entrance of the catalytic site, likely due to the absence of electron-donating substituents capable of mediating polar interactions. In contrast, compound **3o**, despite lacking methoxy groups, retained favorable interactions with Arg120 at the channel entrance, albeit with a slightly altered binding pattern, which may be attributed to the presence of a longer linker.

Overall, this SAR analysis demonstrates that distinct structural features are optimal within different subseries, and that antiplatelet activity in this library is governed by the combined effects of linker length, as well as substituent-driven steric and electronic properties, which collectively determine the ability of each compound to establish key interactions within the COX-1 cyclooxygenase channel.

### 3.6 Molecular Dynamics Simulations

Docking and molecular dynamics (MD) simulations are complementary tools for evaluating the interaction profile of drug candidates. While docking serves as a preliminary step by generating static protein–ligand complexes and providing initial estimates of binding affinities, it has significant limitations in capturing the conformational flexibility inherent to biological systems and the role of solvent in these interactions. To overcome these limitations, MD simulations are often employed after docking to corroborate and refine these preliminary findings.^86^

By reproducing the dynamic behavior of proteins and ligands under physiological conditions, MD simulations allow for a detailed analysis of the stability of binding poses, the presence of alternative interaction modes, and the role of solvent molecules in the interaction. Furthermore, when combined with methods such as MM-PBSA, MD simulations provide more accurate estimates of binding affinities, which is crucial for evaluating drug candidates.^35^ This approach also enables the assessment of the stability of selected compounds within the active site of the target protein,^65^ contributing to a more informed and targeted process.^87^

To assess the equilibrium of the molecular dynamics simulations performed for COX1, we evaluated the Root-Mean-Square Deviation (RMSD), Radius of Gyration (Rg), and Solvent Accessible Surface Area (SASA) of the simulated systems. These included the ligand-bound complexes hCOX1-3a, hCOX1-3g, hCOX1-3j, and hCOX1-3p, and the apo hCOX-1 structure. RMSD reflects the average variation in atom positions relative to the initial structure of the simulation, providing insights into structural fluctuations over time. Rg, in turn, assesses the compactness of the molecular structure, reflecting changes in the spatial arrangement of atoms relative to the center of mass of the complex. SASA estimates the surface area of the complex exposed to the solvent, serving as an indicator of changes in the accessibility of hydrophilic and hydrophobic residues.^56^

As shown in Figure S6, the backbone RMSD values for all the analyzed simulations exhibit plateau behavior, suggesting that the complex structures reached equilibrium and fluctuate around stable average conformations after approximately 50 ns. In contrast, the apo hCOX-1 system exhibits a longer equilibration phase, reaching a steady RMSD regime only after approximately130 ns. Similarly, the protein Rg and SASA values (Figure S7) stabilized at plateau levels from these points onward in the analyzed simulations, suggesting that all the studied systems reached dynamic equilibrium shortly after the initial phase of the trajectories. This behavior suggests that the structural parameters observed during this period are representative of the molecular dynamics of the analyzed complexes.^35^

To assess the stability of the complexes, we first computed the time evolution of ligand RMSD (Figure S6). For all systems, the ligand RMSD profiles exhibit an initial adjustment phase followed by a stable plateau, indicating that the ligands adopt persistent conformations over the simulated time scale. This behavior follows a similar trend to the backbone RMSD observed in Figure S6, in which the protein backbone reaches stable plateau values after approximately 50 ns. The concurrent stabilization of both protein backbone and ligand conformations supports the formation of equilibrated protein–ligand complexes, justifying the use of the equilibrated trajectory segments for subsequent interaction and flexibility analyses.^35,88^

Additionally, the values of minimum distance and the number of contacts established between the protein and the ligand over time were also analyzed (Figure 9). The results indicate that the thioureas remained anchored in the active site of the COX1 protein throughout the trajectory, maintaining a consistently close distance, with average values of 0.196 ± 0.009 nm for **3a**, 0.189 ± 0.009 nm for **3g**, 0.191 ± 0.009 nm for **3j**, and 0.202 ± 0.010 nm for **3p**. Therefore, the stable number of molecular contacts reinforces the persistence of the interaction, while the recorded minimum distances suggest a proximity favorable to the interaction. These findings corroborate that the ligands not only remain in the proposed molecular target’s active site but also establish sufficiently robust interactions to withstand the dynamic forces present in the aqueous solvent simulated environment. This highlights the potential of the thioureas **3a**, **3g**, **3j**, and **3p** as candidates for COX-1 inhibition.^56^

**Figure 9:**
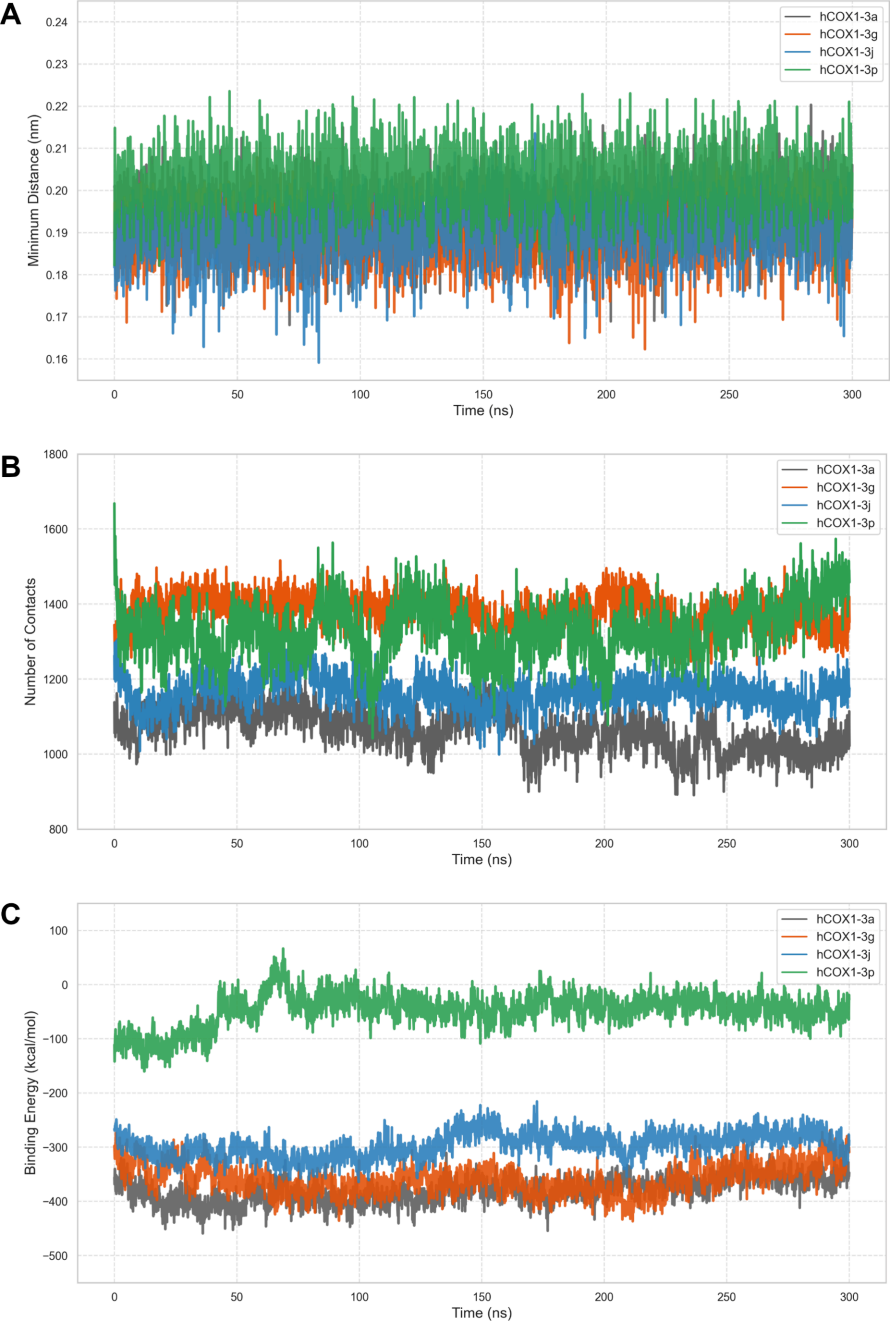
Analysis of contact number, minimum distance, and binding energy of hCOX1 complexes with antiplatelet thiourea derivatives. (A) Minimum distances established between the COX1 protein and the thiourea derivatives **3a**, **3g**, **3j**, and **3p**. (B) Number of contacts established between the COX1 protein and the thiourea derivatives **3a**, **3g**, **3j**, and **3p**. (C) Binding energy computed for the complexes formed between the hCOX1 protein and the thiourea derivatives **3a**, **3g**, **3j**, and **3p**.

To complement this analysis, MM-PBSA analysis was then performed on the MD trajectories to estimate the binding affinities of the hCOX1-thiourea derivative complexes. This method provides a precise estimation of the binding free energy in protein–ligand systems, surpassing the limitations of molecular docking approaches. Combined with molecular dynamics simulations, which offer a comprehensive sampling of binding modes, MM-PBSA calculations incorporate the effects of conformational fluctuations and binding entropy.^89^ These considerations significantly enhance the predictive accuracy of this method, albeit at a higher computational cost.^62^

As shown in Figure 9, the results revealed that the derivatives **3a** (−376.3 ± 46.9 kJ/mol), **3g** (−362.7 ± 49.9 kJ/mol), and **3j** (−293.9 ± 32.2 kJ/mol) exhibited a substantially higher average binding affinities to the COX-1 target compared to **3p** (−37.8 ± 64.8 kJ/mol). These data suggest that **3a**, **3g**, and **3j** form energetically more favorable complexes with hCOX-1, which may be related to stronger molecular interactions.

Despite the relatively low binding energy predicted for **3p**, all evaluated complexes exhibited negative free energy values, indicating that their formation is energetically favorable and occurs spontaneously, despite differences in binding strength.^35^

The analysis of the individual components of the binding energy computed through the MM-PBSA method (Figure S8) indicates that electrostatic interactions were the primary stabilizing forces in the complexes formed by the derivatives **3a**, **3g**, and **3j** with hCOX-1, possibly due to a greater formation of polar interactions with the active site of the molecular target. The establishment of these interactions may be related to a stronger and more stable fit of the **3a**, **3g**, and **3j** derivatives in the active site of hCOX-1, resulting in higher binding affinity (Figure S8). Nevertheless, for the derivative **3p**, van der Waals interactions played a substantially more relevant role in the formation of the complex with hCOX-1. This suggests that the stability of this complex is predominantly determined by interactions between apolar groups, possibly including hydrophobic contacts. This interaction profile directly reflects the structural composition of this compound, which is primarily composed of aliphatic and aromatic groups.

The analysis of solvation energies revealed that only apolar contributions favor the retention of ligands in hydrophobic environments, such as the cavities in the active site of hCOX-1. However, these apolar contributions have a relatively limited impact compared to the total computed energy (Figure S8). Conversely, the polar solvation energy stands as the main opposition to the interaction, disfavoring the retention of ligands in the active sites. These results highlight the importance of electrostatic and hydrophobic interactions as the primary factors favoring these complexes.^56^ We then assessed the persistence of key protein–ligand interactions during the simulations (Figure 10) to better understand the mechanistic basis underlying the energetic trends revealed by the MMPBSA analysis (Figure 9). For this purpose, we quantified the occupancy of each protein–ligand interaction, i.e., the percentage of simulation frames in which that contact was present during the simulation.^35^ This analysis focused on following interactions: hydrogen bonds, cation−π interactions, π-stacking, salt bridges, halogen bonds, and hydrophobic contacts.

**Figure 10:**
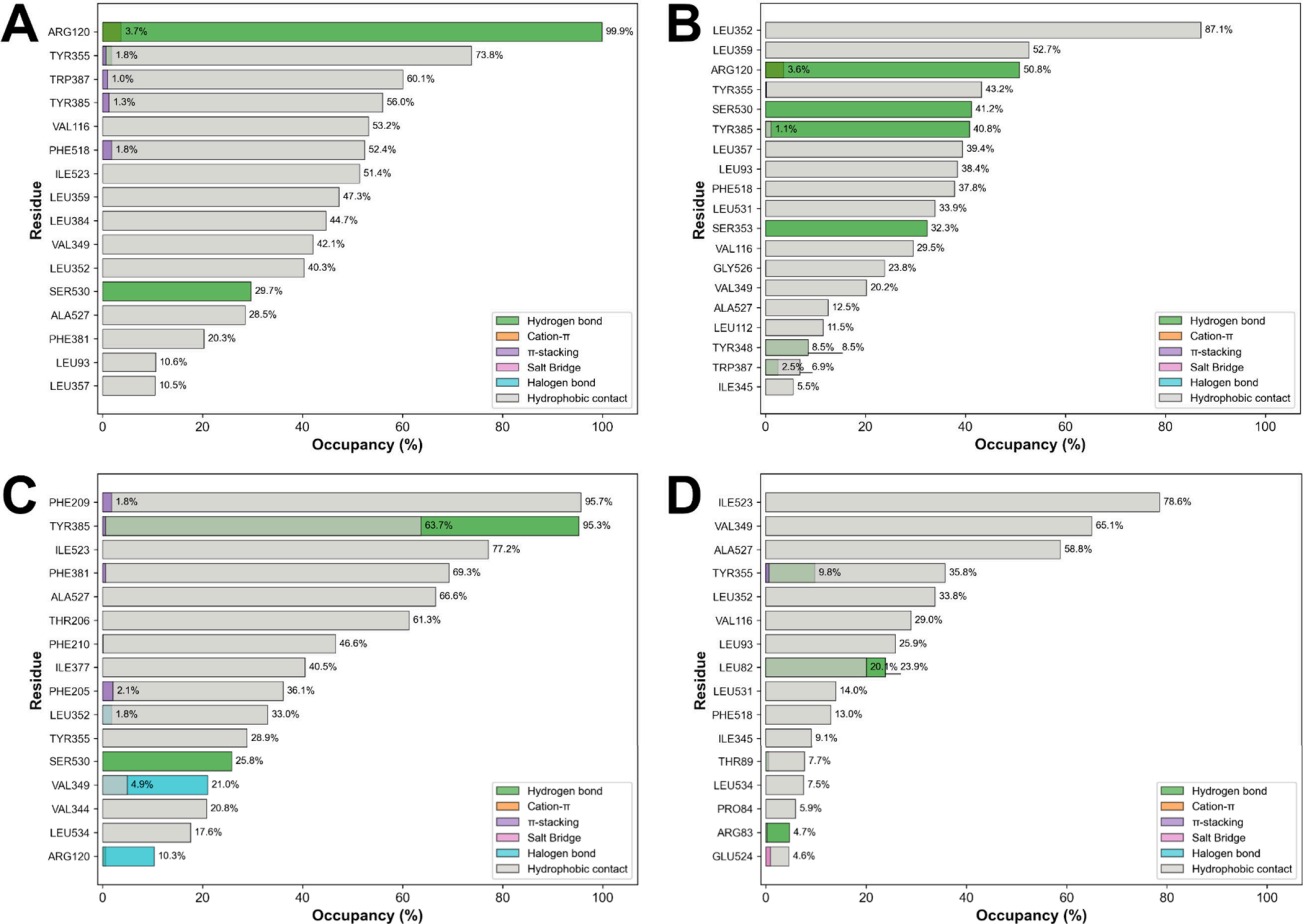
Protein–ligand interaction occupancy profiles for COX-1 complexes with thiourea derivatives **3a**, **3g**, **3j**, and **3p** obtained from molecular dynamics simulations. Panels show the percentage occupancy of key protein–ligand interactions along the simulation time for (A) **3a**, (B) **3g**, (C) **3j**, and (D) **3p**. Only residues exhibiting significant interaction occupancies (≥5%) are shown. Amino acid residues are represented using their standard three-letter codes, as follows: ALA: Alanine, ARG: Arginine, GLU: Glutamic Acid, GLY: Glycine, ILE: Isoleucine, LEU: Leucine, PHE: Phenylalanine, PRO: Proline, SER: Serine, THR: Threonine, TRP: Tryptophan, TYR: Tyrosine, and VAL: Valine.

Consistent with the MM-PBSA findings (Figure 9), our occupancy analysis indicates that derivatives **3a**, **3g**, and **3j** exhibit a substantially more favorable interaction profile within the hCOX-1 active site than **3p** (Figure 10). In particular, the high occupancies of stabilizing polar interactions appear to be the primary drivers of the enhanced binding affinity of **3a**, **3g**, and **3j**, while an extensive network of hydrophobic contacts provides a complementary stabilizing. A hydrogen bond in protein–ligand complexes typically contributes a larger binding free energy per individual contact (≈2–5 kcal·mol^–1^) than a single hydrophobic contact, which is mainly stabilized by van der Waals interactions and usually contributes ≤ 1 kcal·mol^–1^ per contact. This difference arises because hydrogen bonds are governed by direct electrostatic attraction and require strict geometric alignment between donor and acceptor groups, leading to a substantial and directional enthalpic contribution to the binding free energy. In contrast, individual hydrophobic contacts are weaker and largely nondirectional, providing smaller per-contact enthalpic stabilization, with their overall contribution becoming significant only when many such interactions act cooperatively.^90,91^

Compound **3a** exhibits persistent interactions with key residues within the hCOX-1 cyclooxygenase active site (Figure 10). Notably, it forms sustained hydrogen bonds with Arg120 and Ser530, with the interaction involving Arg120 persisting in 99.9% of the simulation frames. This finding is particularly relevant, as Arg120 plays a central role in anchoring substrates through ionic interactions at the entrance of the active site. Although Ser530, which is acetylated by aspirin—a well-known COX-1 inhibitor—is not directly involved in catalytic turnover, its interaction likely enhances inhibitor stabilization within the active site and may prevent arachidonic acid from accessing the catalytic pocket. In parallel, **3a** establishes an extensive network of hydrophobic contacts with Tyr355 and Tyr385. Tyr355, together with Arg120, forms a functional gate at the entrance of the cyclooxygenase channel, contributing to substrate and inhibitor positioning through hydrophobic and aromatic interactions. Meanwhile, Tyr385 plays a role in the catalytic activity of COX-1 by facilitating the abstraction of a hydrogen atom from arachidonic acid—a key step in prostaglandin synthesis.^76,77,81–84^ Therefore, the combined engagement of these residues suggests an efficient occupation of the active site and provides a structural basis for the enhanced binding affinity estimated by the MMPBSA analysis for this complex (Figure 9).

Similarly, compound **3g** exhibits sustained polar interactions with key cyclooxygenase residues, forming hydrogen bonds with Arg120, Tyr385, and Ser530 for 40–50% of the trajectory (Figure 10). It also forms hydrogen bonds with Ser353, providing additional, though less persistent, contacts. An extensive hydrophobic network is also observed for **3g**, further stabilizing the complex and complementing the polar interactions. Compound **3j** presents the most extensive interaction network among the series, with very high occupancies of hydrophobic contacts, alongside recurrent hydrogen bonding with Tyr385 and Ser530, and additional halogen bond interactions with Arg120 and Val349. Remarkably, hydrogen bonding between derivative **3j** and the catalytic residue Tyr385 persists for over 95% of the trajectories. This broad and persistent network likely underlies **3j**’s favorable binding affinity, despite structural differences relative to **3a** and **3g**.

The sustained polar and hydrophobic interactions observed in the MD simulations (Figure 10) for the derivatives **3a**, **3g**, and **3j** might play a key role in shaping the favorable energetic profiles demonstrated by the MM-PBSA analysis (Figure S8). The dominant electrostatic component observed for **3a**, **3g**, and **3j** (Figure S8) may be primarily attributed to persistent hydrogen bonds, with **3j** also forming stabilizing halogen bonds within the cyclooxygenase site (Figure 10).

Additionally, the hydrogen bonding patterns with key cyclooxygenase residues suggest a potential mechanism for their antiplatelet activity, as shown *in vitro* by AA-induced inhibition assays (Figure 2). These interactions likely stabilize the inhibitors within the active site, thereby preventing substrate access and contributing to the observed inhibitory effects. Interestingly, **3p** exhibited markedly weaker predicted binding affinities (Figure S8) and a consistently reduced interaction network in the occupancy analysis compared to the other three active thioureas in the series, i.e., **3a**, **3g**, and **3j**. Derivative **3p** interacts minimally with the key residue Tyr355 (<10% occupancy) and forms additional, albeit limited, hydrogen bonds with Leu82 (23.9%) and Arg83 (4.7%) (Figure 10). This sparse hydrogen-bonding network is consistent with the minimal contribution of the electrostatic term to **3p**’s binding energy (Figure S8). Its interaction profile is dominated by hydrophobic contacts (Figure 10), consistent with the predominance of the van der Waals component (Figure S8), which likely arises from interactions between **3p** and aliphatic or aromatic residues within the cyclooxygenase site. As these interactions are weaker and less directional than hydrogen or halogen bonds, they typically provide only modest stabilization,^90,91^ which could contribute to the overall lower binding affinity predicted for **3p**.

Finally, we investigated whether ligand binding influences the flexibility of hCOX-1 regions by performing RMSF analyses, comparing the dynamics of ligand-bound complexes with the apo hCOX-1 structure (Figure 11). In all simulations, RMSF analysis indicated that the N-terminal domain (residues 33–72) and its adjacent region up to residue 100 ^92^ consistently exhibit elevated flexibility. This behavior is expected, as the N-terminal domain in cyclooxygenases are not deeply integrated into the catalytic core. Consequently, it experiences reduced structural constraints and forms fewer stabilizing internal contacts than the rigid core regions, leading to diminished conformational restraints^93^ that are reflected in higher RMSF values. Notably, the inherent flexibility of this region was markedly attenuated upon binding to **3a** and **3j**, whereas complexes with **3p** and **3g** retained fluctuations comparable to the apo state. This behavior suggests a selective, ligand-dependent modulation of N-terminal domain dynamics even at long-range from the cyclooxygenase binding site.

**Figure 11:**
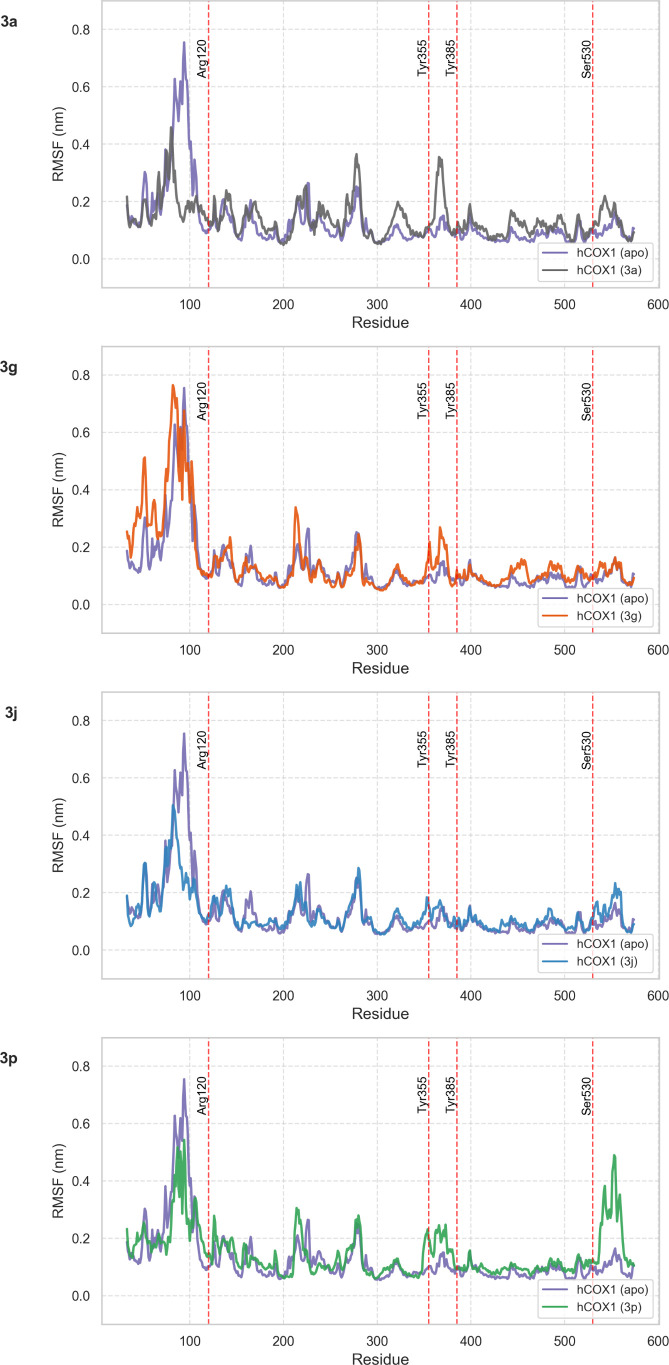
Per-residue Root-Mean-Square Fluctuation (RMSF) of hCOX-1 in apo and ligand-bound states over the simulations. For each panel, the RMSF of apo hCOX-1 (purple) is superimposed on those of the ligand-bound complexes **3a**–hCOX1, **3g**–hCOX1, and **3j**-hCOX1. Vertical red dashed lines indicate key cyclooxygenase residues: Arg120, Tyr355, Tyr385, and Ser530. Amino acid residues are represented using their standard three-letter codes, as follows: ARG: Arginine, TYR: Tyrosine, and SER: Serine.

Beyond the N-terminal region, the central catalytic core of hCOX-1 displays relatively low RMSF values in all simulations, reflecting its densely packed organization.^94^ Nevertheless, subtle flexibility alterations emerged within specific protein core regions upon ligand binding. In particular, the catalytic-associated segment encompassing residues 355–380 exhibited consistently increased RMSF values across all ligand-bound complexes, albeit to different extents relative to the apo state. This region is located adjacent to structural elements that govern substrate access to the active site, encompassing the key residue Tyr355.^37^ In contrast, the remaining key hCOX-1 residues, i.e., Arg120, Tyr385, and Ser530, did not exhibit significant flexibility changes upon ligand binding, maintaining RMSF profiles comparable to those observed in the apo state.

Extending the analysis toward the C-terminal portion of the enzyme, residues 535–575 generally exhibited only modest deviations upon binding to the active thioureas. An exception was observed for the **3p**–hCOX-1 complex, which displayed enhanced flexibility in this region.

## 4 Conclusions

In summary, this study reports the successful design, synthesis, and biological evaluation of a new series of piperazine-derived thioureas as potential antiplatelet agents targeting COX-1. The compounds were efficiently obtained under mild conditions, demonstrating high yields and synthetic versatility. Among the derivatives, four compounds (**3a**, **3g**, **3j**, and **3p**) exhibited potent inhibition of arachidonic acid-induced platelet aggregation in human platelet-rich plasma, with activities comparable to or greater than that of aspirin. These active thioureas did not interfere with coagulation pathways or induce hemolysis, indicating a selective action on primary hemostasis and a favorable hemocompatibility profile.

The *in* silico pharmacokinetic and toxicological evaluation supported their drug-like character and low predicted risk for mutagenicity, hepatotoxicity, and cardiotoxicity, reinforcing their safety for further development. Molecular docking and molecular dynamics simulations revealed that these compounds establish stable interactions with key residues within the COX-1 catalytic site (Arg120, Tyr385, and Ser530), consistent with their observed *in vitro* inhibitory effects. Together, MM-PBSA and interaction occupancy analyses consistently indicated a favorable binding profile for the active thioureas, suggesting that complex stabilization was predominantly driven by hydrogen bonding and, in the case of **3j**, additional halogen-bond interactions. In contrast, the stability of the **3p**–hCOX-1 complex was primarily governed by hydrophobic contacts.

Altogether, our findings highlight piperazine thioureas as promising molecular scaffolds for the development of next-generation antiplatelet agents with improved safety and selectivity profiles, offering opportunities to improve the quality of life for individuals affected by or at risk of CVDs.

## Supplementary Material



## Notes

Figure S1: Three-dimensional structures of cyclooxygenase-1; (A) Human COX-1 (hCOX-1) and (B) ovine COX-1 (oCOX-1) complexed with mofezolac, displayed as ribbon representations highlighting secondary structural elements, including α-helices, β-sheets, and loop regions; The hCOX-1 structure is shown in green, whereas the oCOX-1 structure is shown in blue; The cocrystallized ligand mofezolac and the heme prosthetic group are depicted in stick representation and colored in orange and purple, respectively; Figure S2: Docking simulation of mofezolac within the cyclooxygenase active site of human COX-1 (hCOX-1); The predicted binding mode of the hCOX-1–mofezolac complex is shown for the hCOX-1 structure (PDB ID: 6Y3C); Mofezolac is depicted as orange sticks and colored by atom type, while interacting active-site residues are shown as purple sticks and labeled accordingly; Hydrogen-bond interactions are represented by green dashed lines, and salt bridges by pink dashed lines; residues forming hydrophobic contacts with the inhibitor are also displayed; A complementary two-dimensional interaction diagram is provided to facilitate visualization of the protein–ligand interaction network, with all interaction distances explicitly indicated; Amino acid residues are represented using their standard three-letter codes, as follows: ALA: Alanine, ARG: Arginine, GLY: Glycine, HIS: Histidine, ILE: Isoleucine, LEU: Leucine, PHE: Phenylalanine, SER: Serine, TRP: Tryptophan, TYR: Tyrosine, VAL: Valine; Figure S3: Superposition of the predicted binding poses of thiourea derivatives within the cyclooxygenase active site of human COX-1; Compounds 3a (green), 3g (cyan), 3j (magenta), and 3p (orange) are shown as sticks and color-coded by atom type; Amino acid residues within the cyclooxygenase active site are depicted as purple sticks and labeled accordingly; The protein backbone is represented as a transparent gray ribbon, displaying the secondary structure elements of the enzyme; Amino acid residues are represented using their standard three-letter codes, as follows: ALA: Alanine, ARG: Arginine, ILE: Isoleucine, LEU: Leucine, MET: Methionine, SER: Serine, TRP: Tryptophan, TYR: Tyrosine, VAL: Valine; Figure S4: Docking of inactive thioureas 3b, 3c, 3d, 3e, 3f, and 3h on the COX-1 receptor; The ligands are represented by green sticks and colored by atom type; The residues involved in interactions with the ligand are shown as purple sticks, colored by atom type, and labeled according to the corresponding amino acid; Hydrogen bonds are represented by dashed green lines, salt bridges by dashed pink lines, π-π interactions by dashed purple lines, and cation-π interactions by dashed orange lines; Residues involved in hydrophobic contacts with the inhibitor are also shown; Amino acid residues are represented using their standard three-letter codes, as follows: ALA: Alanine, ARG: Arginine, GLU: Glutamic Acid, ILE: Isoleucine, LEU: Leucine, MET: Methionine, PHE: Phenylalanine, PRO: Proline, SER: Serine, THR: Threonine, TRP: Tryptophan, TYR: Tyrosine, VAL: Valine; Figure S5: Docking of inactive thioureas **3i**, **3k**, **3l**, **3m**, **3n**, and **3o** on the COX-1 receptor; The ligands are represented by green sticks and colored by atom type; The residues involved in interactions with the ligand are shown as purple sticks, colored by atom type, and labeled according to the corresponding amino acid; Hydrogen bonds are represented by dashed green lines, salt bridges by dashed pink lines, π–π interactions by dashed purple lines, and cation−π interactions by dashed orange lines; Residues involved in hydrophobic contacts with the inhibitor are also shown; Amino acid residues are represented using their standard three-letter codes, as follows: ALA: Alanine, ARG: Arginine, ILE: Isoleucine, LEU: Leucine, MET: Methionine, PHE: Phenylalanine, SER: Serine, TRP: Tryptophan, TYR: Tyrosine, VAL: Valine; Figure S6: Backbone and ligand root-mean-square-deviation (RMSD) analyses. (A) Backbone RMSD plot over time for hCOX1 complexed with thioureas **3a**, **3g**, **3j**, and **3p**, as well as apo hCOX1; (B) Ligand RMSD plot over time for hCOX1 complexed with thioureas **3a**, **3g**, **3j**, and **3p**; Figure S7: Radius of gyration (Rg) and solvent-accessible surface area (SASA) analyses of the COX1 complex with antiplatelet thiourea derivatives; (A) Rg plot over time for COX1 complexed with thioureas **3a**, **3g**, **3j**, and **3p**; (B) SASA plot over time for COX1 complexed with thioureas **3a**, **3g**, **3j**, and **3p**; Figure S8: Predicted total binding energy for the complexes formed between the antiplatelet thiourea derivatives and the COX-1 target calculated by the MM-PBSA method, as well as their individual energy terms; (A) COX1-3a complex; (B) COX1-3g complex; (C) COX1-3j complex; (D) COX1-3p complex (PDF)
