# Supplementary material for: Piperazine-Thiourea Hybrids as Novel Antiplatelet Agents Targeting COX-1: Synthesis, in Vitro, and in Silico Evaluation
Source: ACS Omega. 2026 Apr 1;11(14):21903–25. doi: 10.1021/acsomega.5c12576 (PMC13084458; doi:10.1021/acsomega.5c12576)
Supplement: Supplementary file 1 [file ao5c12576_si_001.pdf]

Supporting Information

Piperazine-Thiourea Hybrids as Novel Antiplatelet Agents Targeting COX-1: Synthesis, *In Vitro*, and *In Silico* Evaluation

Gabriel Rodrigues Coutinho Pereira[1,2], Gil Mendes Viana[2], Mariana Borges Huber[1], Pryscila Santiago Rodrigues[3], Anna Rita Santiago de Paula Gonçalves[3], Plínio Cunha Sathler[3], Carlos Rangel Rodrigues[1], Bárbara de Azevedo Abrahim-Vieira[1,*], Lucio Mendes Cabral[2,*]

[1]Laboratory of Molecular Modeling and QSAR; [2]Laboratory of Industrial Pharmaceutical Technology; [3]Laboratory of Experimental Hemostasis; Federal University of Rio de Janeiro, Rio de Janeiro, Brazil, Carlos Chagas Filho Avenue, 373, 21941-902.

*Corresponding authors: lmcabral2@yahoo.com.br, barbara.abrahim@ufrj.br

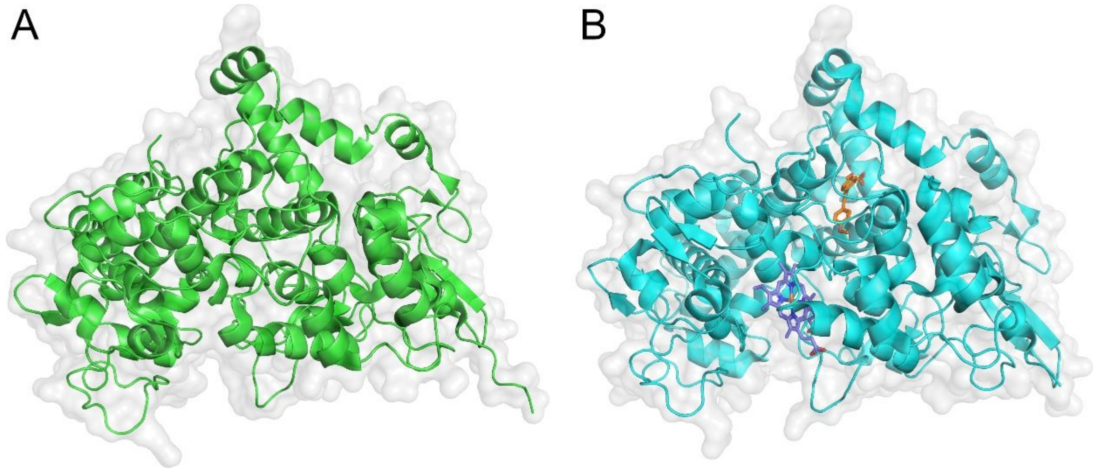

Figure S1. Three-dimensional structures of cyclooxygenase-1. (A) Human COX-1 (hCOX-1) and (B) ovine COX-1 (oCOX-1) complexed with mofezolac, displayed as ribbon representations highlighting secondary structural elements, including α-helices, β-sheets, and loop regions. The hCOX-1 structure is shown in green, whereas the oCOX-1 structure is shown in blue. The co-crystallized ligand mofezolac and the heme prosthetic group are depicted in stick representation and colored in orange and purple, respectively.

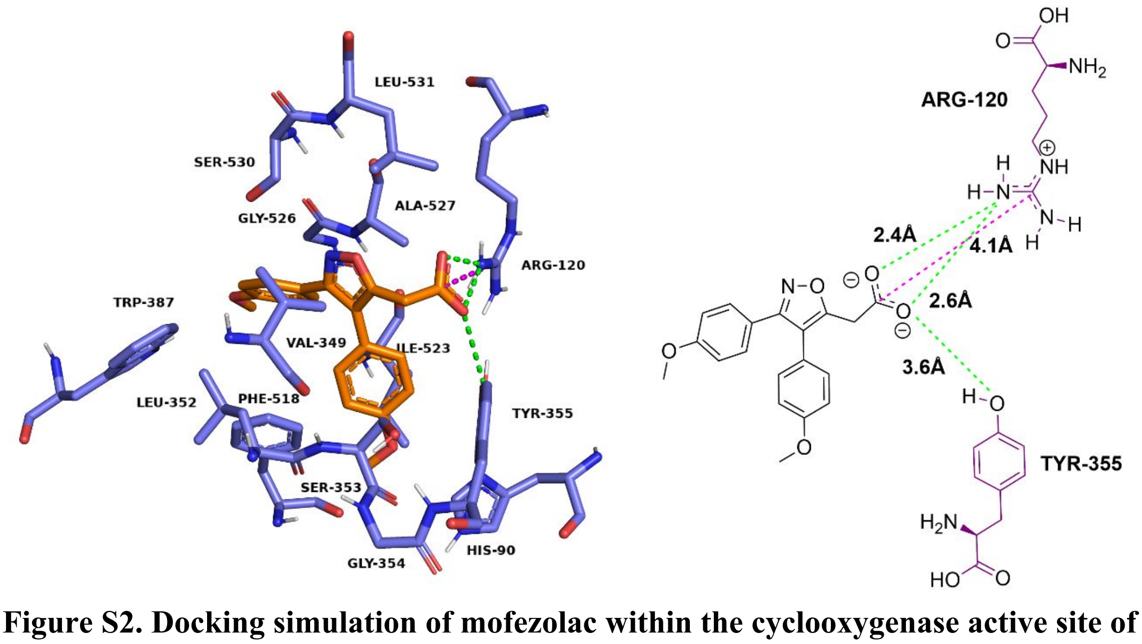

Figure S2. Docking simulation of mofezolac within the cyclooxygenase active site of human COX-1 (hCOX-1). The predicted binding mode of the hCOX-1–mofezolac complex is shown for the hCOX-1 structure (PDB ID: 6Y3C). Mofezolac is depicted as orange sticks and colored by atom type, while interacting active-site residues are shown as purple sticks and labeled accordingly. Hydrogen-bond interactions are represented by green dashed lines, and salt bridges by pink dashed lines; residues forming hydrophobic contacts with the inhibitor are also displayed. A complementary two-dimensional interaction diagram is provided to facilitate visualization of the protein–ligand interaction network, with all interaction distances explicitly indicated.

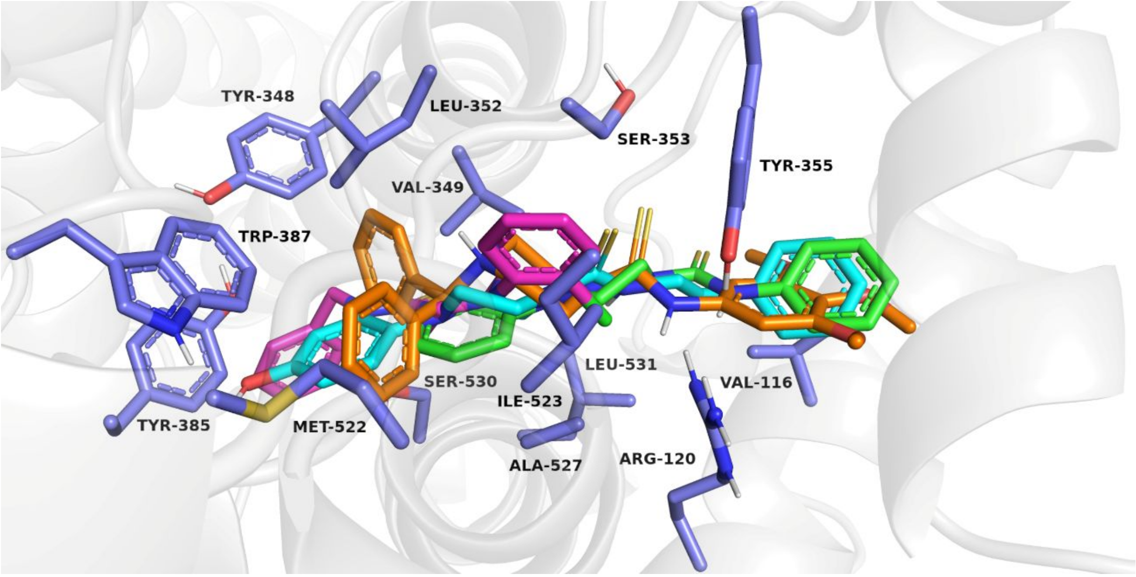

Figure S3. Superposition of the predicted binding poses of thiourea derivatives within the cyclooxygenase active site of human COX-1. Compounds 3a (green), 3g (cyan), 3j (magenta), and 3p (orange) are shown as sticks and color-coded by atom type. Amino acid residues within the cyclooxygenase active site are depicted as purple sticks and labeled accordingly. The protein backbone is represented as a transparent gray ribbon, displaying the secondary structure elements of the enzyme.

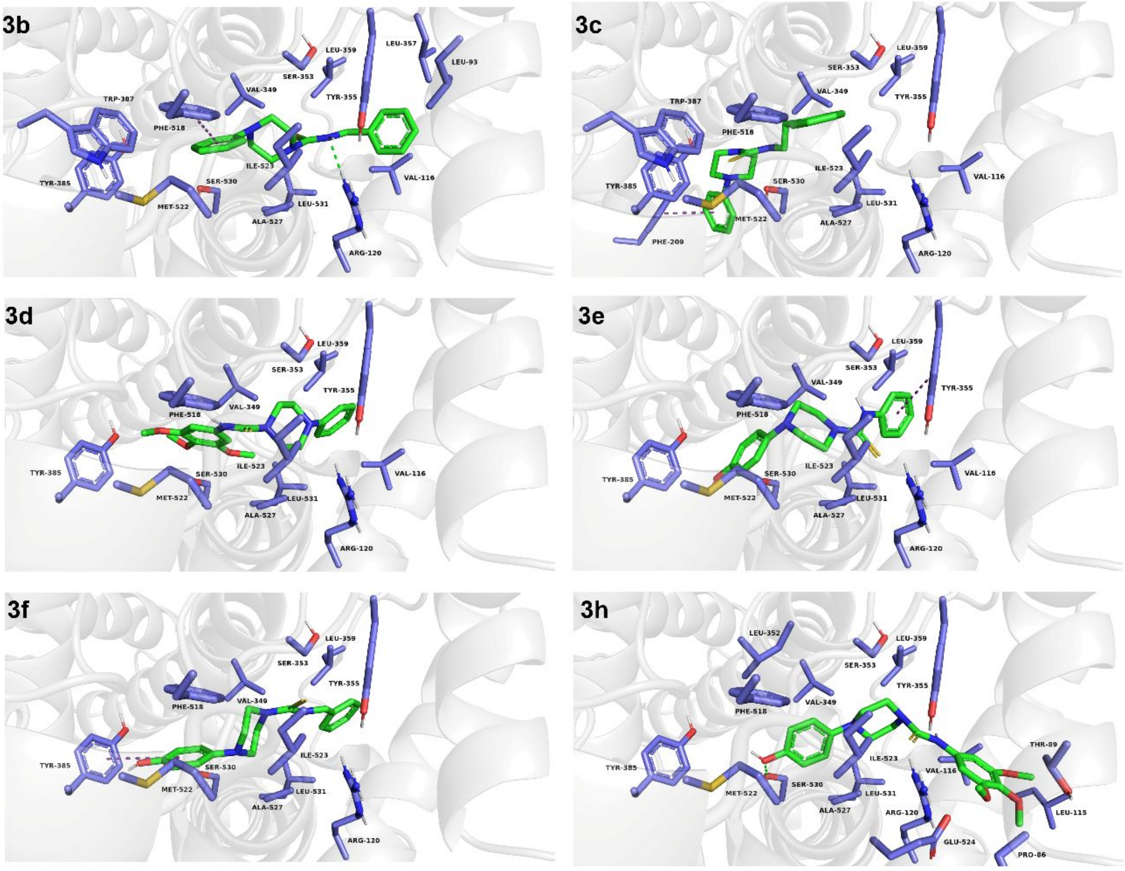

Figure S4. Docking of inactive thioureas 3b, 3c, 3d, 3e, 3f, and 3h on the COX-1 receptor. The ligands are represented by green sticks and colored by atom type. The residues involved in interactions with the ligand are shown as purple sticks, colored by atom type, and labeled according to the corresponding amino acid. Hydrogen bonds are represented by dashed green lines, salt bridges by dashed pink lines, π-π interactions by dashed purple lines, and cation-π interactions by dashed orange lines. Residues involved in hydrophobic contacts with the inhibitor are also shown.

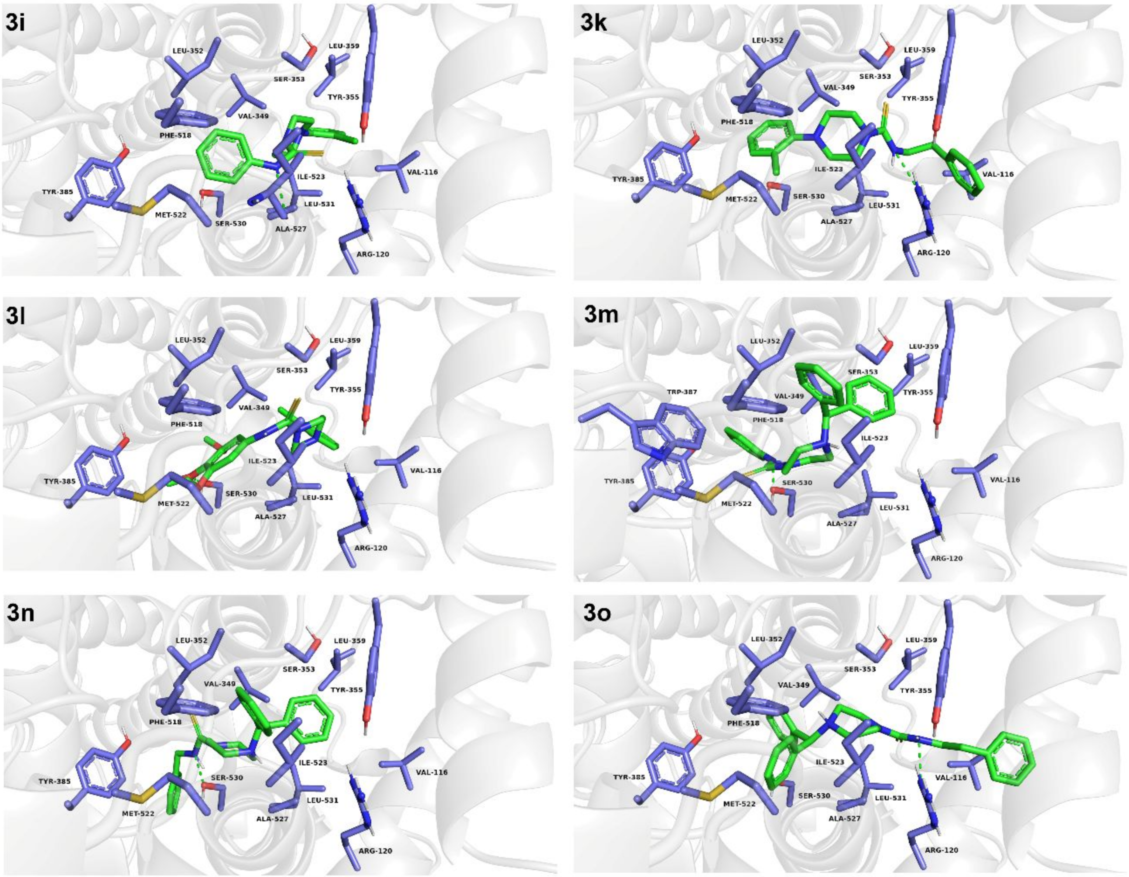

Figure S5. Docking of inactive thioureas 3i, 3k, 3l, 3m, 3n, and 3o on the COX-1 receptor. The ligands are represented by green sticks and colored by atom type. The residues involved in interactions with the ligand are shown as purple sticks, colored by atom type, and labeled according to the corresponding amino acid. Hydrogen bonds are represented by dashed green lines, salt bridges by dashed pink lines, π-π interactions by dashed purple lines, and cation-π interactions by dashed orange lines. Residues involved in hydrophobic contacts with the inhibitor are also shown.

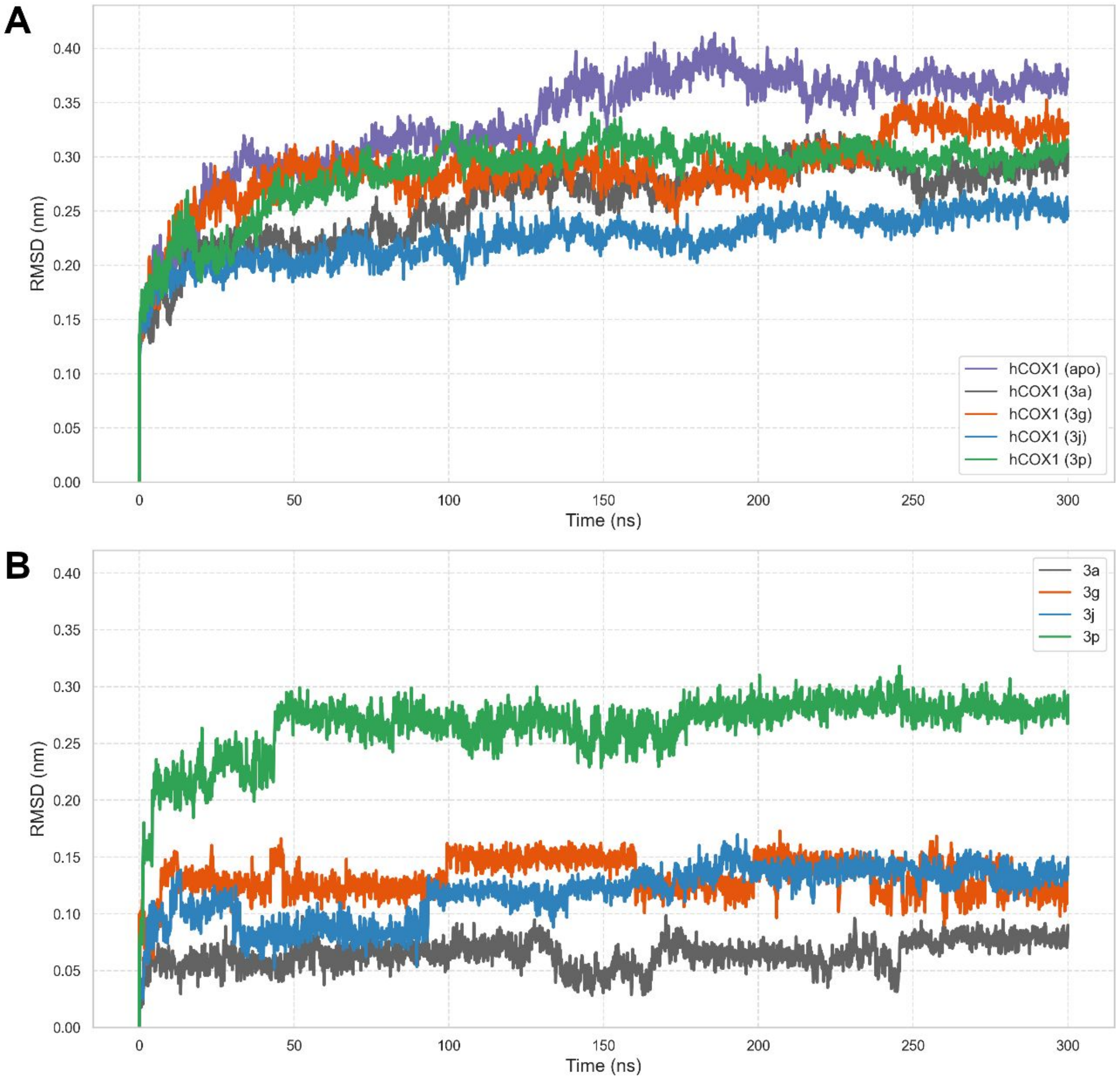

Figure S6. Backbone and ligand root-mean-square-deviation (RMSD) analyses. (A) Backbone RMSD plot over time for hCOX1 complexed with thioureas 3a, 3g, 3j, and 3p, as well as apo hCOX1. (B) Ligand RMSD plot over time for hCOX1 complexed with thioureas 3a, 3g, 3j, and 3p.

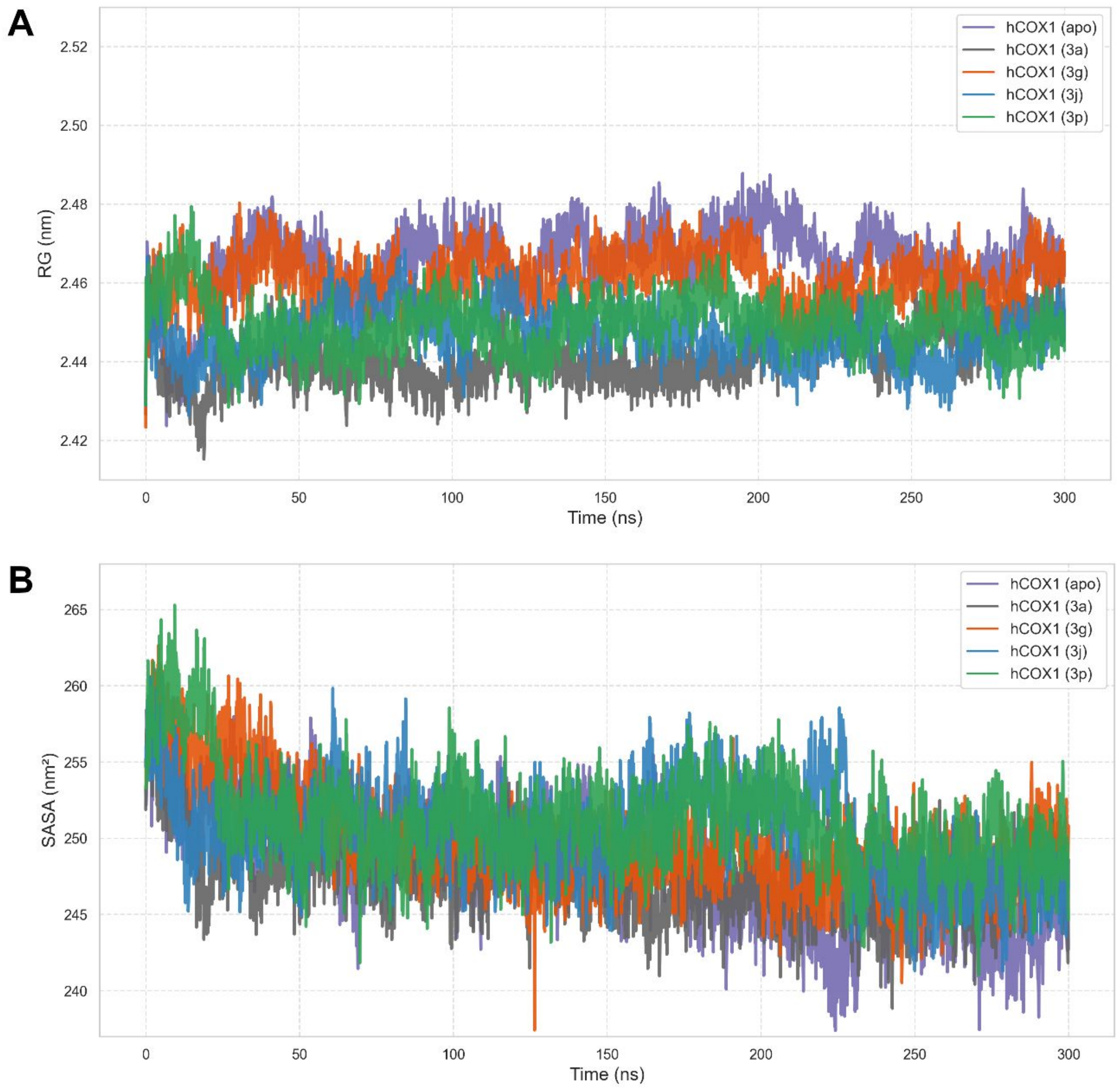

Figure S7. Radius of gyration (Rg) and solvent-accessible surface area (SASA) analyses of the COX1 complex with anti-platelet thiourea derivatives. (A) Rg plot over time for COX1 complexed with thioureas 3a, 3g, 3j, and 3p. (B) SASA plot over time for COX1 complexed with thioureas 3a, 3g, 3j, and 3p.

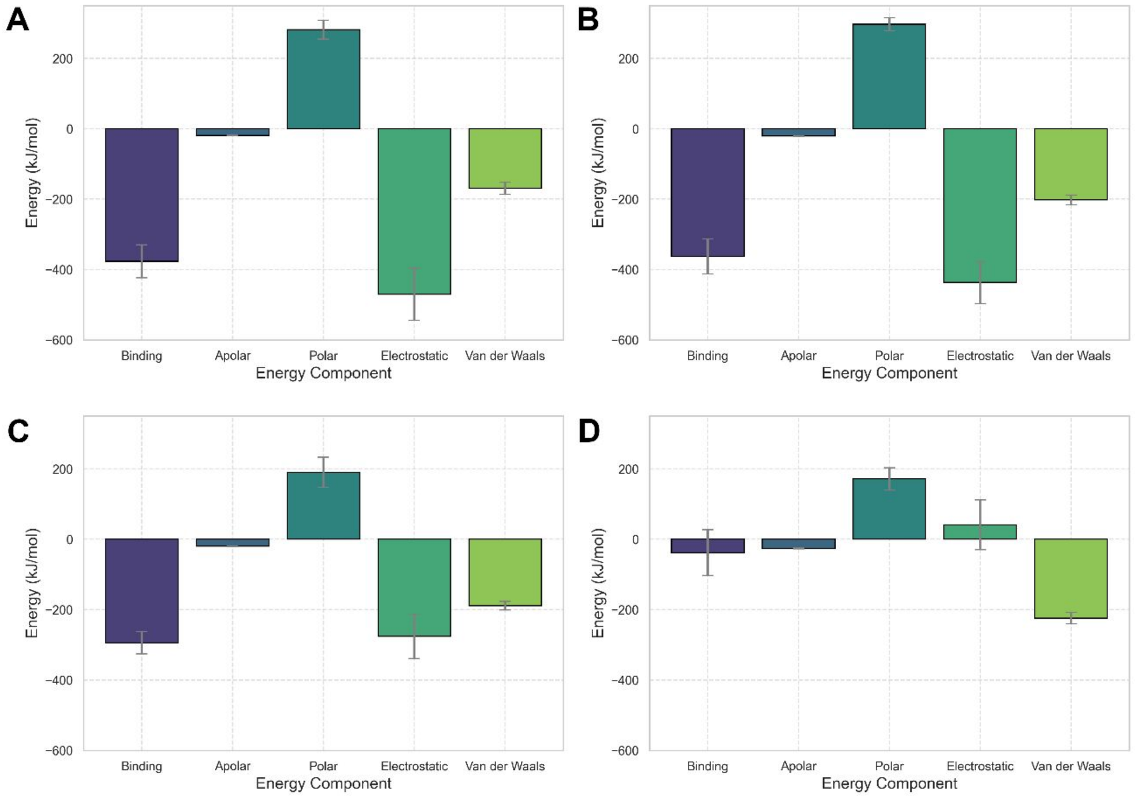

Figure S8. Predicted total binding energy for the complexes formed between the anti-platelet thiourea derivatives and the COX-1 target calculated by the MM-PBSA method, as well as their individual energy terms. (A) COX1-3a complex. (B) COX1-3g complex. (C) COX1-3j complex. (D) COX1-3p complex.

Copies of ^{1}H-NMR, ^{13}C-NMR, FT-IR and HR-MS Spectra for Thiourea Derivatives

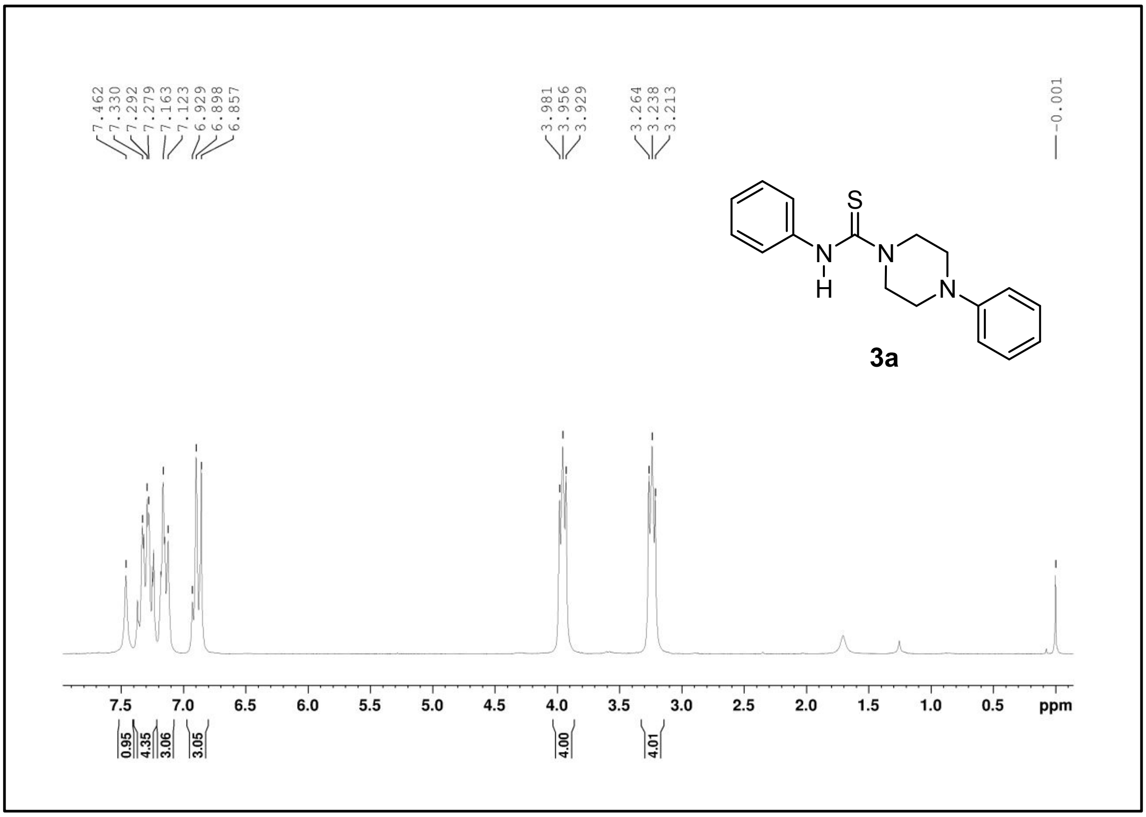

(A)

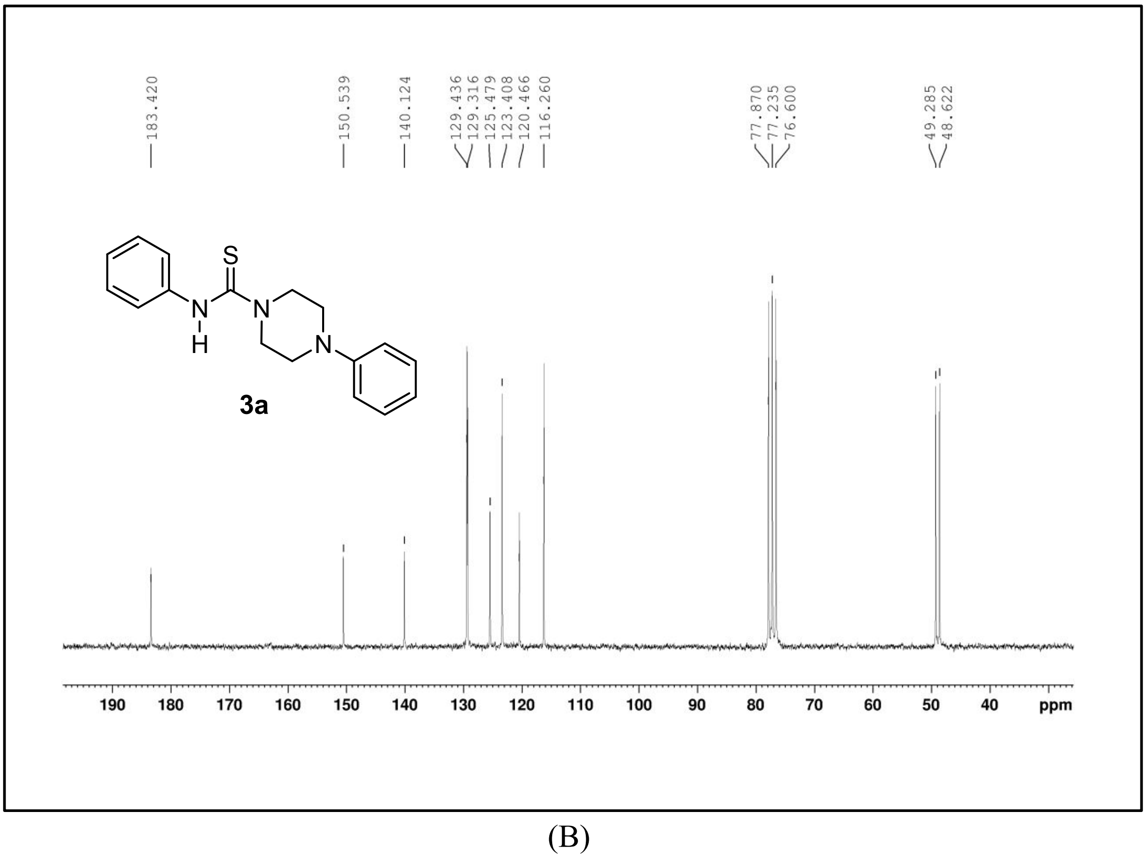

(B)

Figure S8. ^{1}H-NMR spectrum (A) and ^{13}C-NMR spectrum (B) of thiourea **3a**.

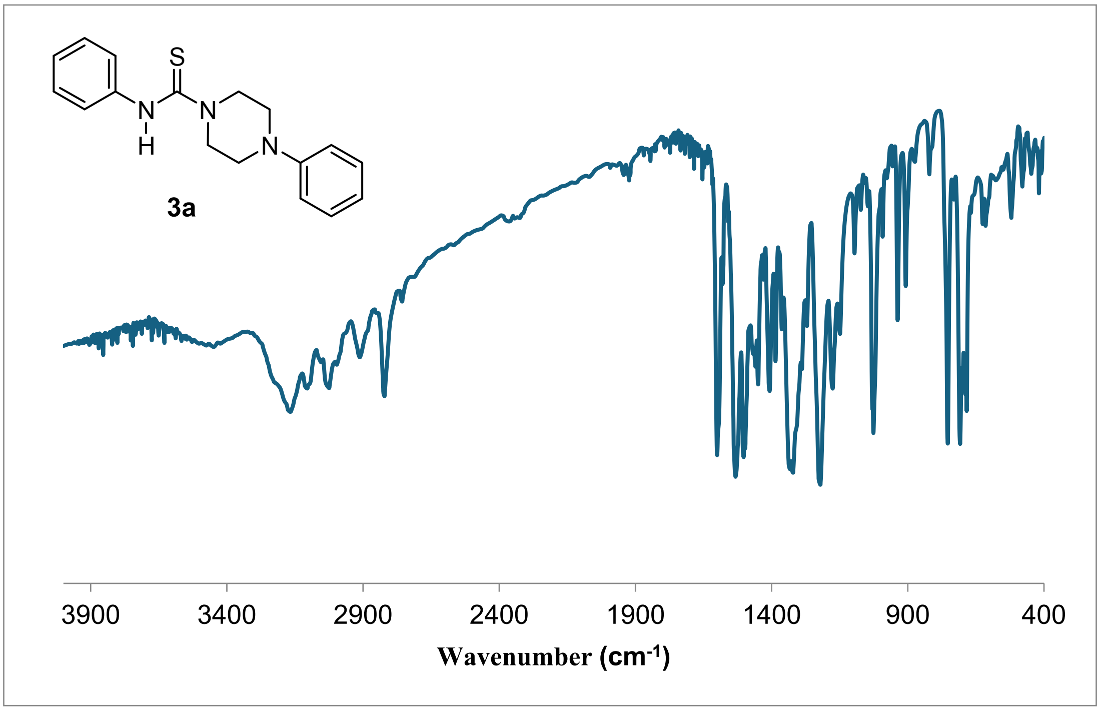

Figure S9. FT-IR spectrum of thiourea **3a**.

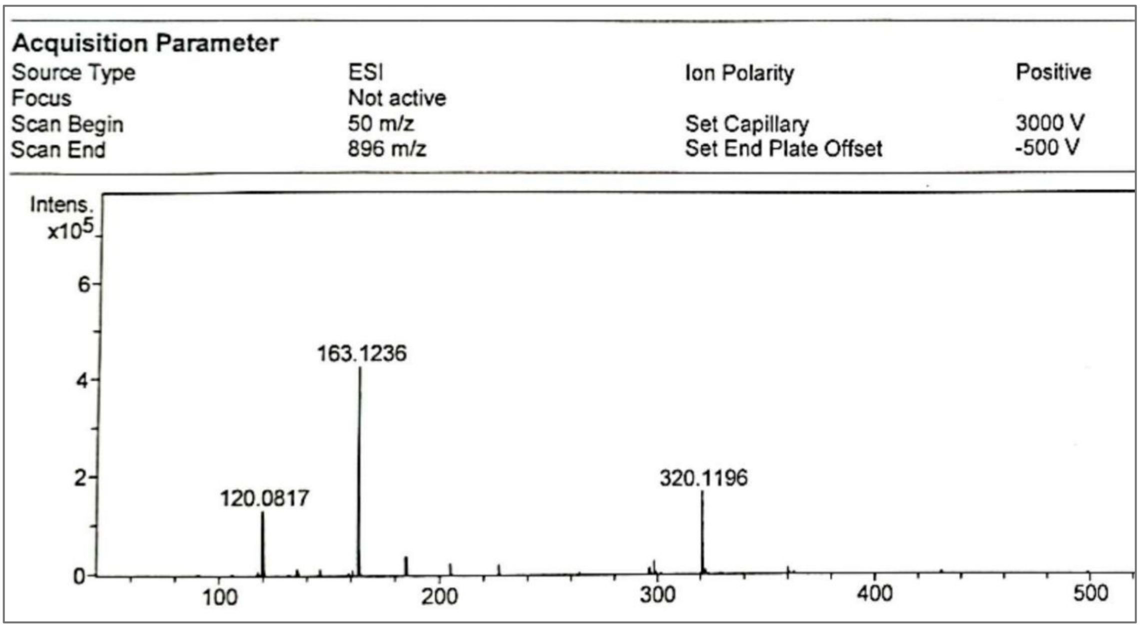

Figure S10. HR-MS spectrum of thiourea **3a**.

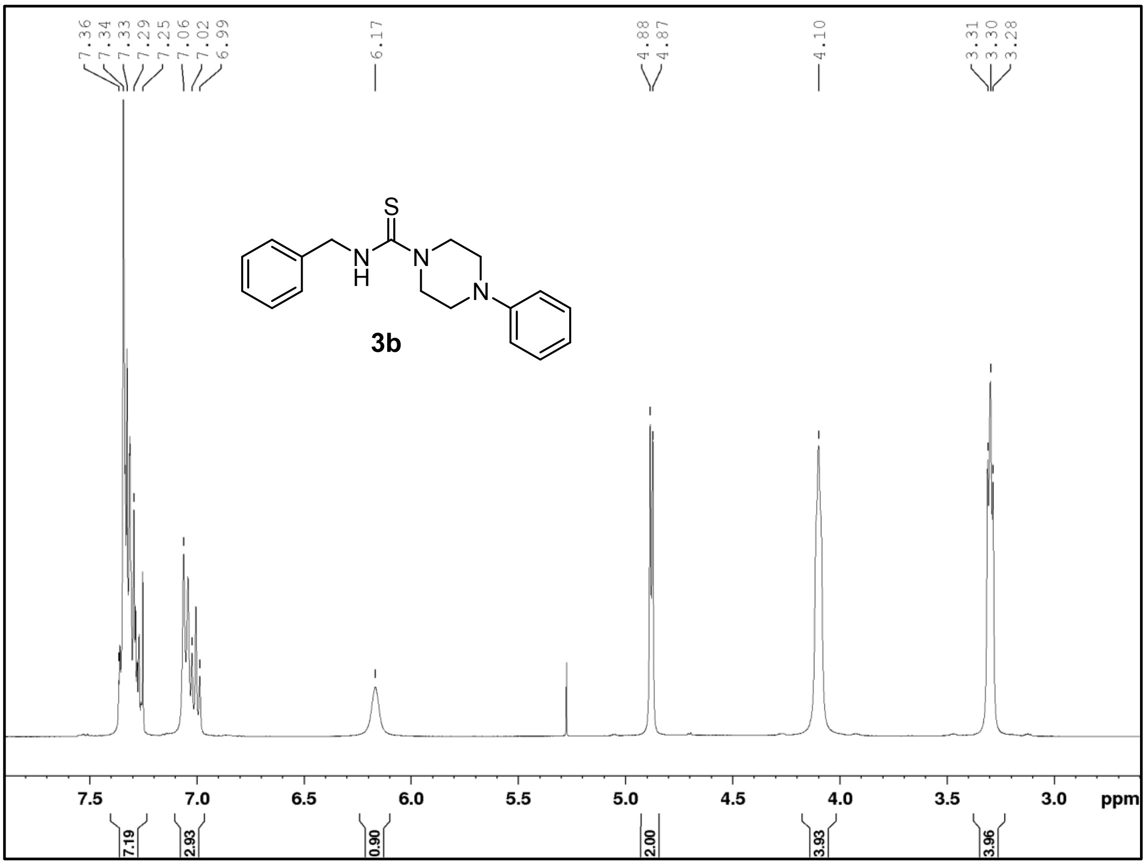

(A)

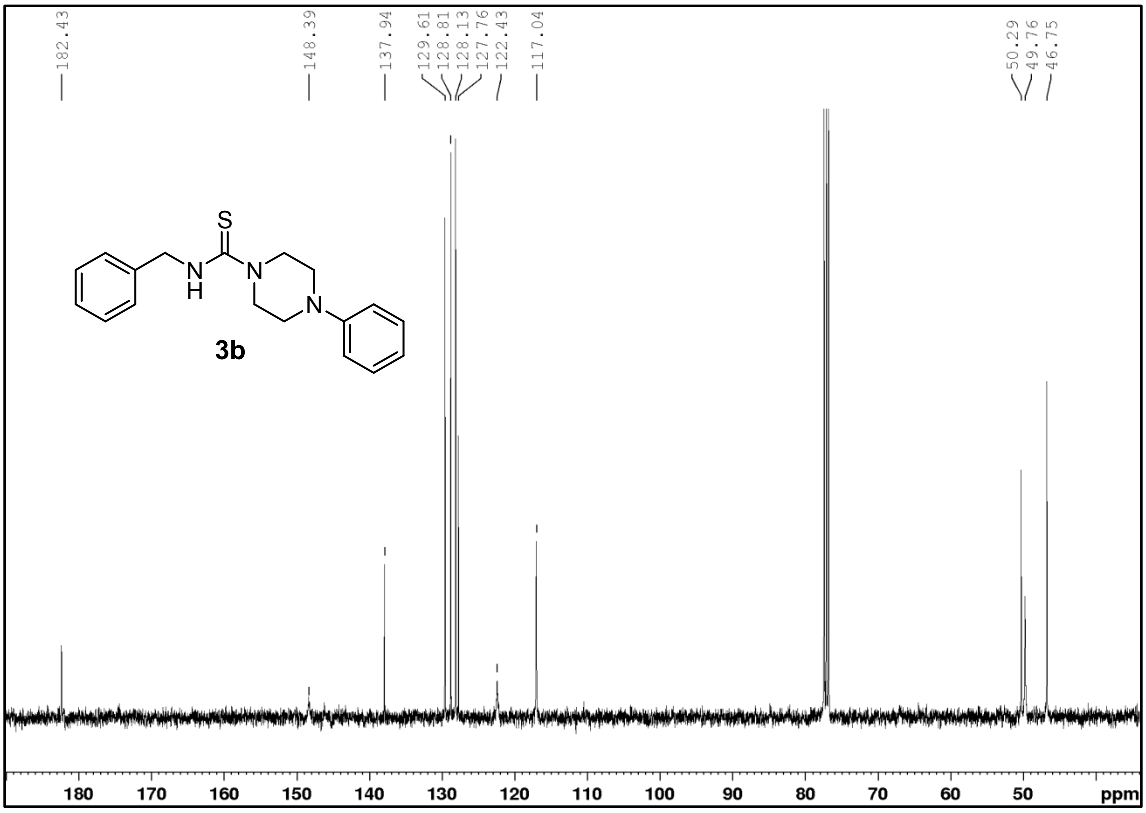

(B)

Figure S11. ^{1}H-NMR spectrum (A) and ^{13}C-NMR spectrum (B) of thiourea **3b**.

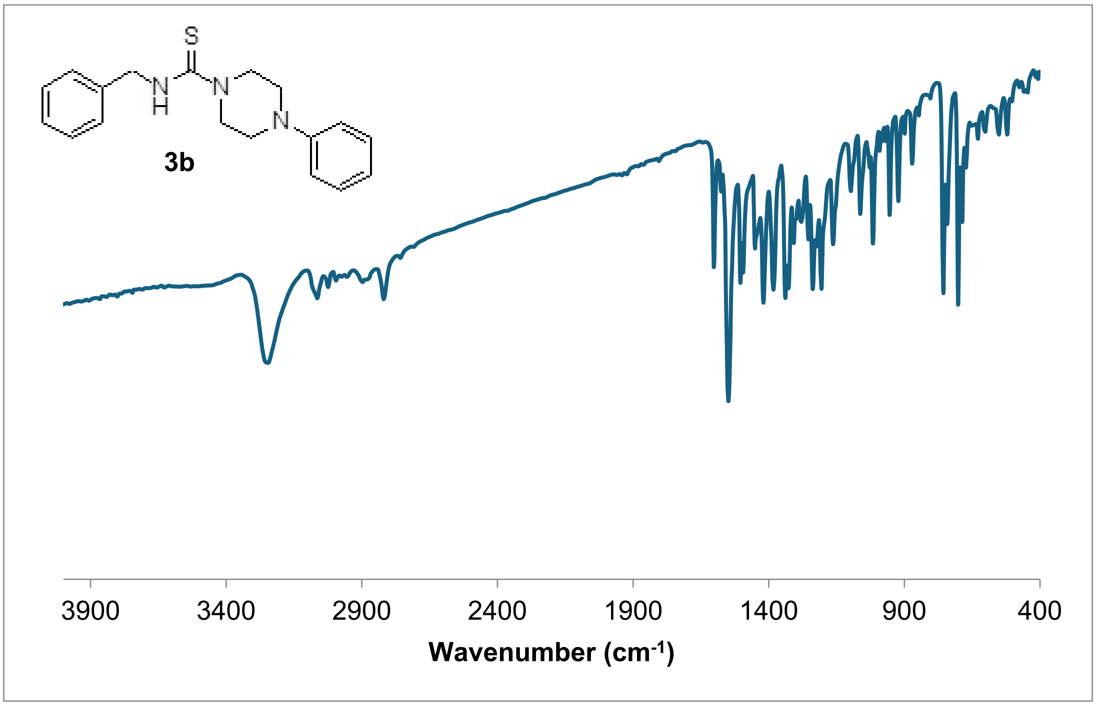

Figure S12. FT-IR spectrum of thiourea **3b**.

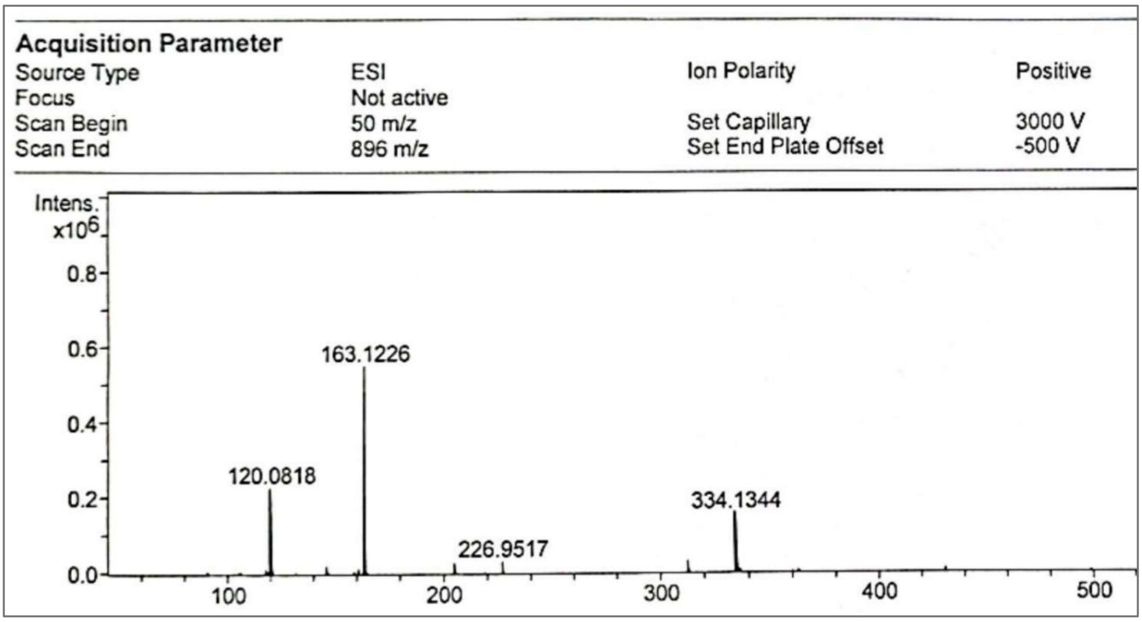

Figure S13. HR-MS spectrum of thiourea **3b**.

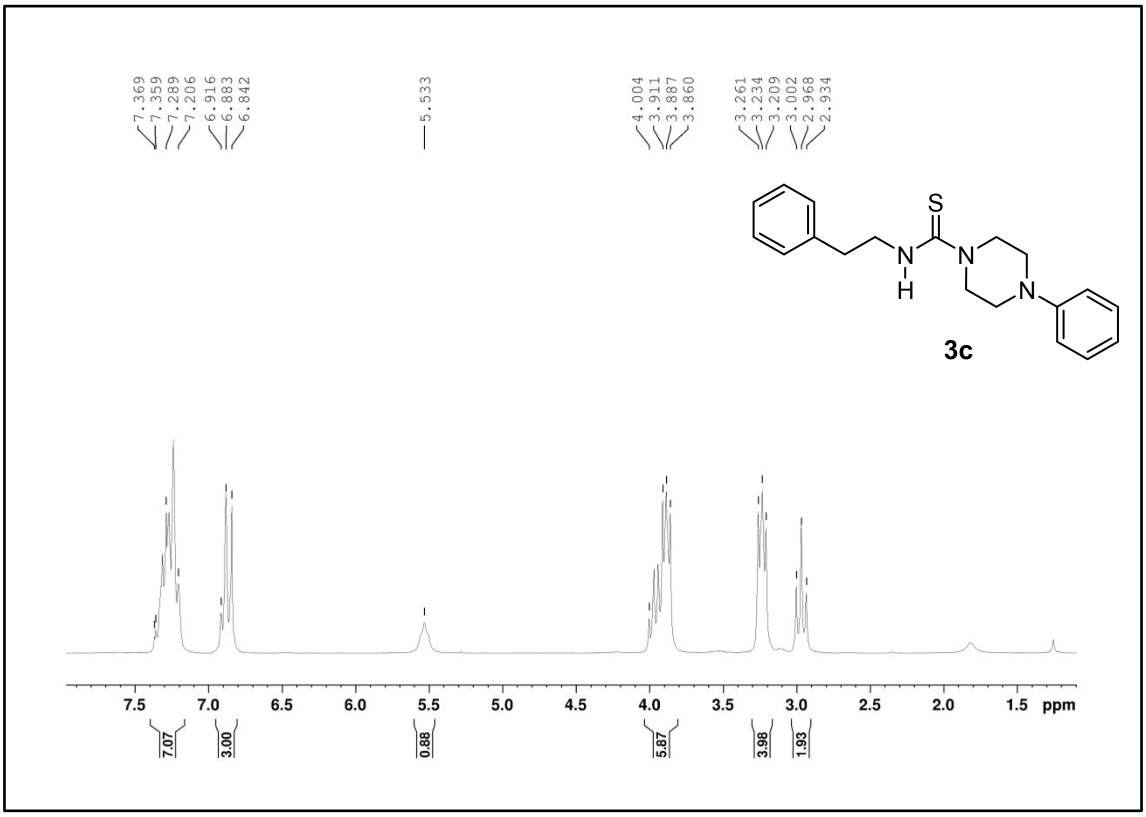

(A)

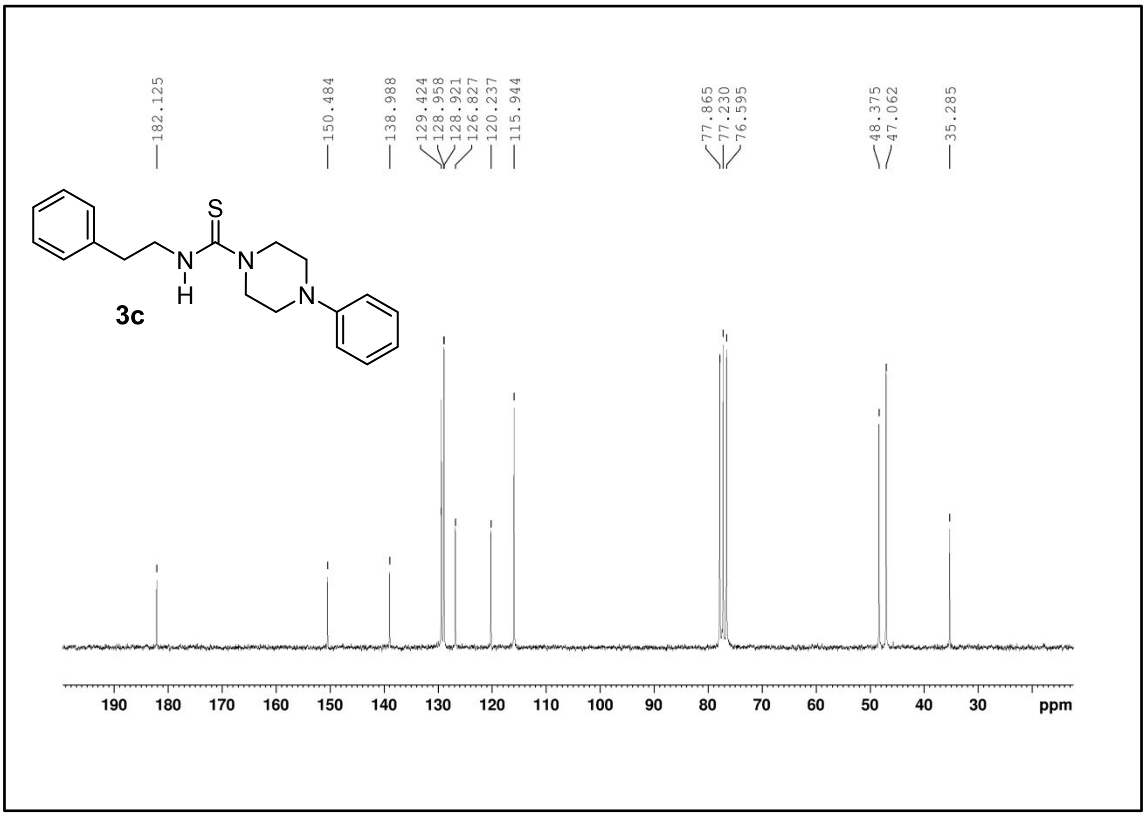

(B)

Figure S14. ^{1}H-NMR spectrum (A) and ^{13}C-NMR spectrum (B) of thiourea **3c**.

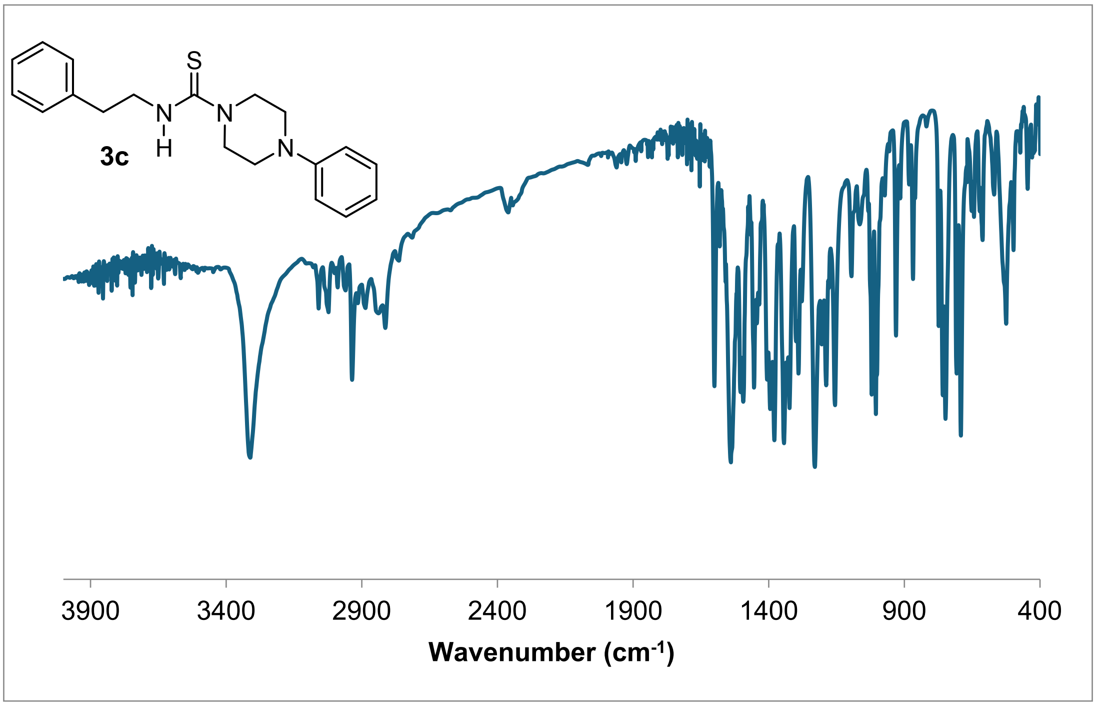

Figure S15. FT-IR spectrum of thiourea **3c**.

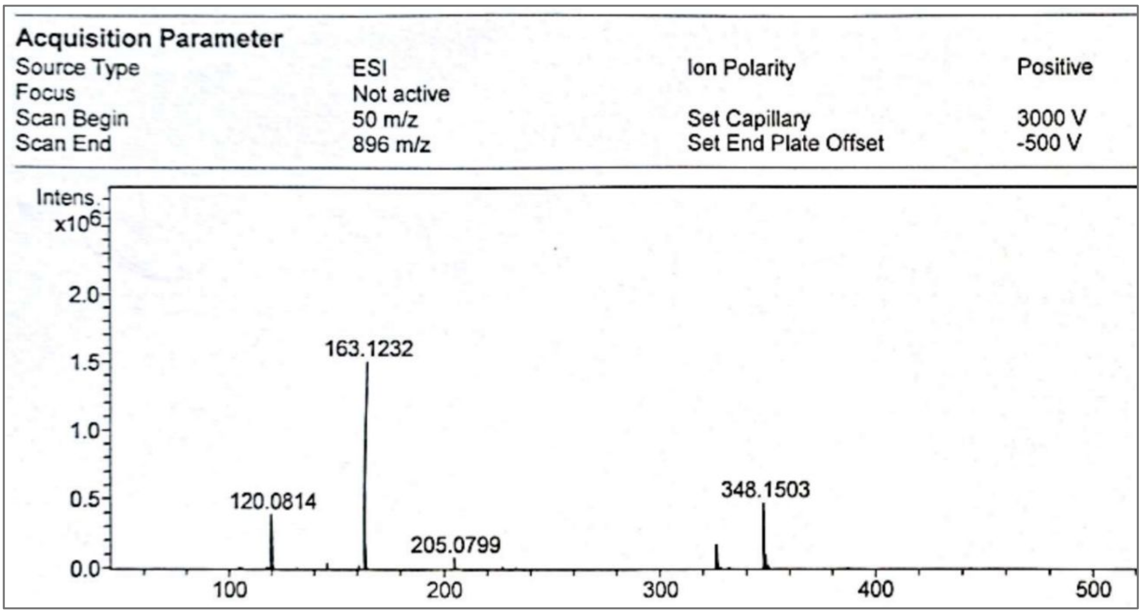

Figure S16. HR-MS spectrum of thiourea **3c**.

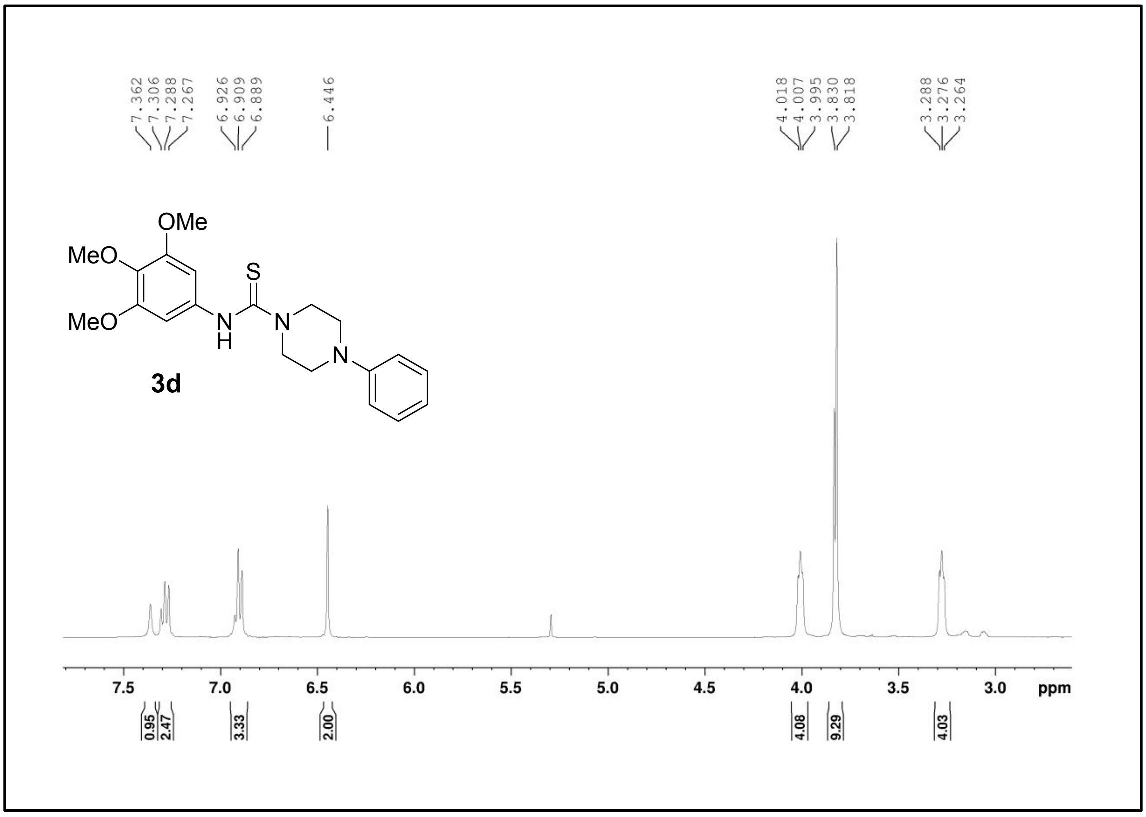

(A)

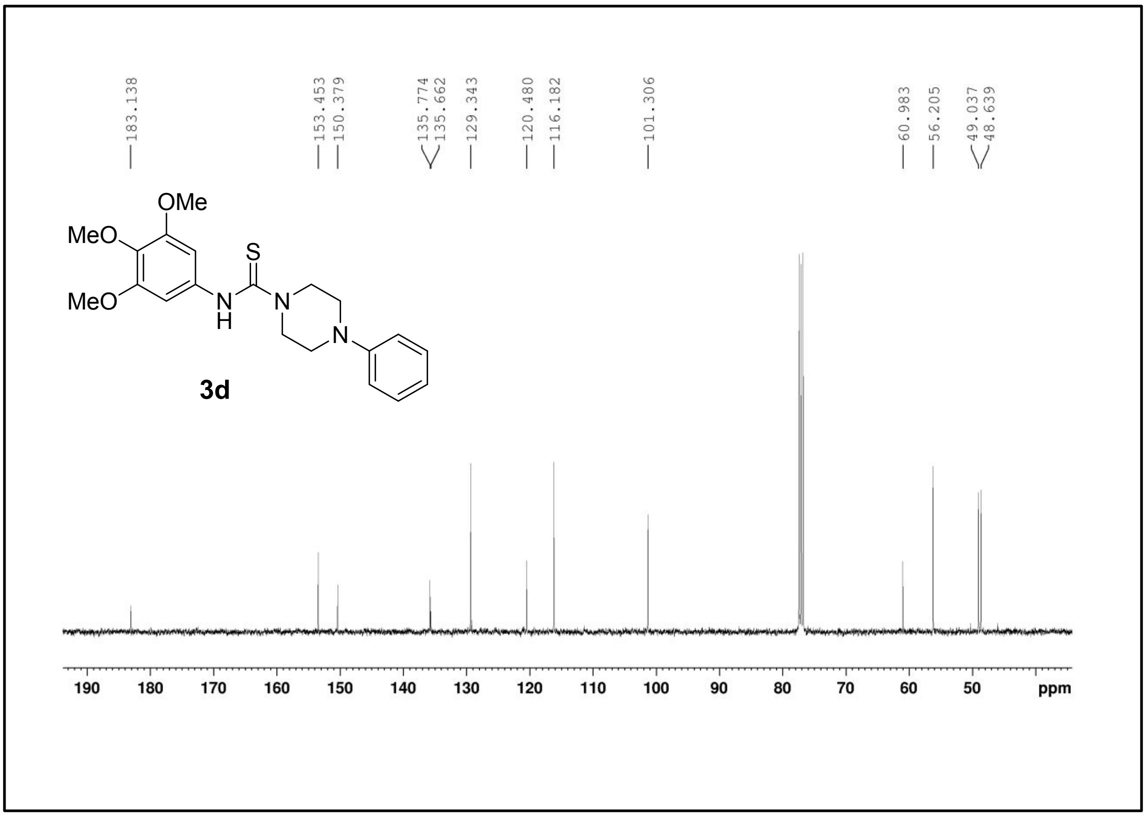

(B)

Figure S17. ^{1}H-NMR spectrum (A) and ^{13}C-NMR spectrum (B) of thiourea **3d**.

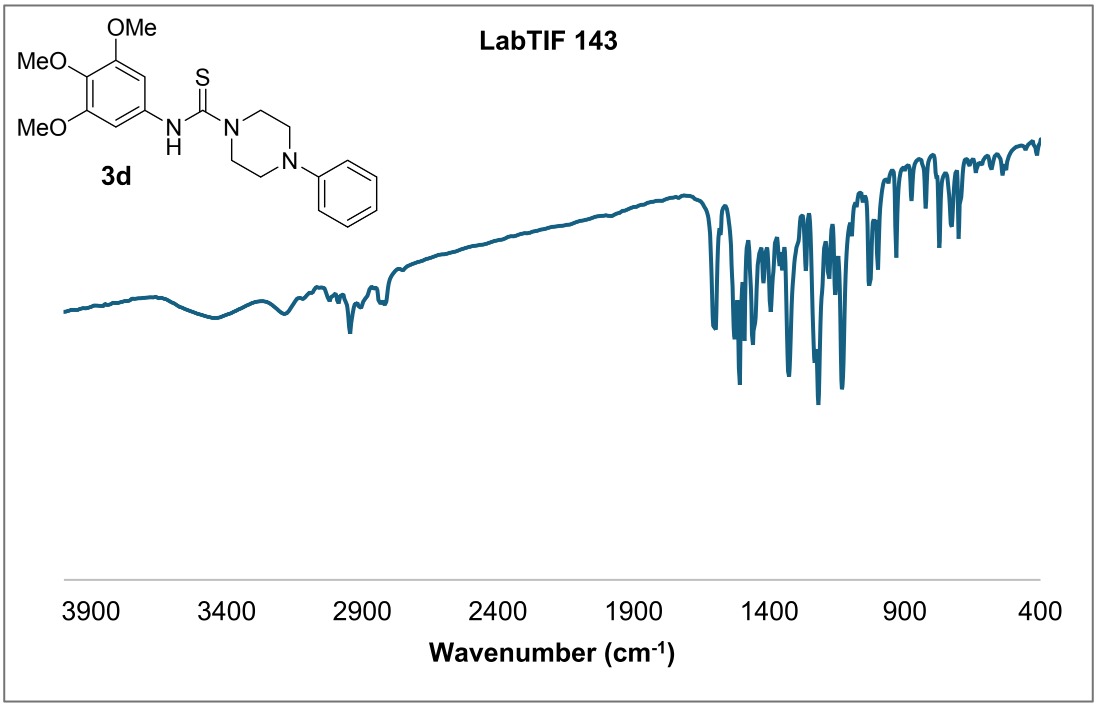

Figure S18. FT-IR spectrum of thiourea **3d**.

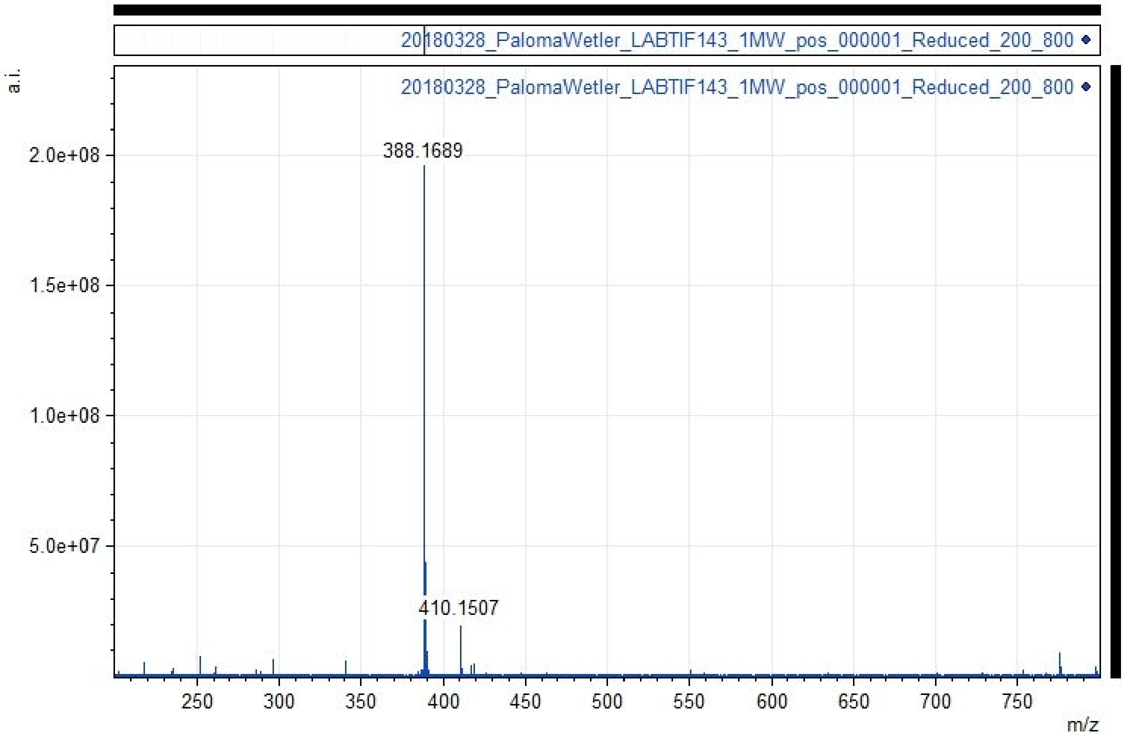

Figure S19. HR-MS spectrum of thiourea **3d**.

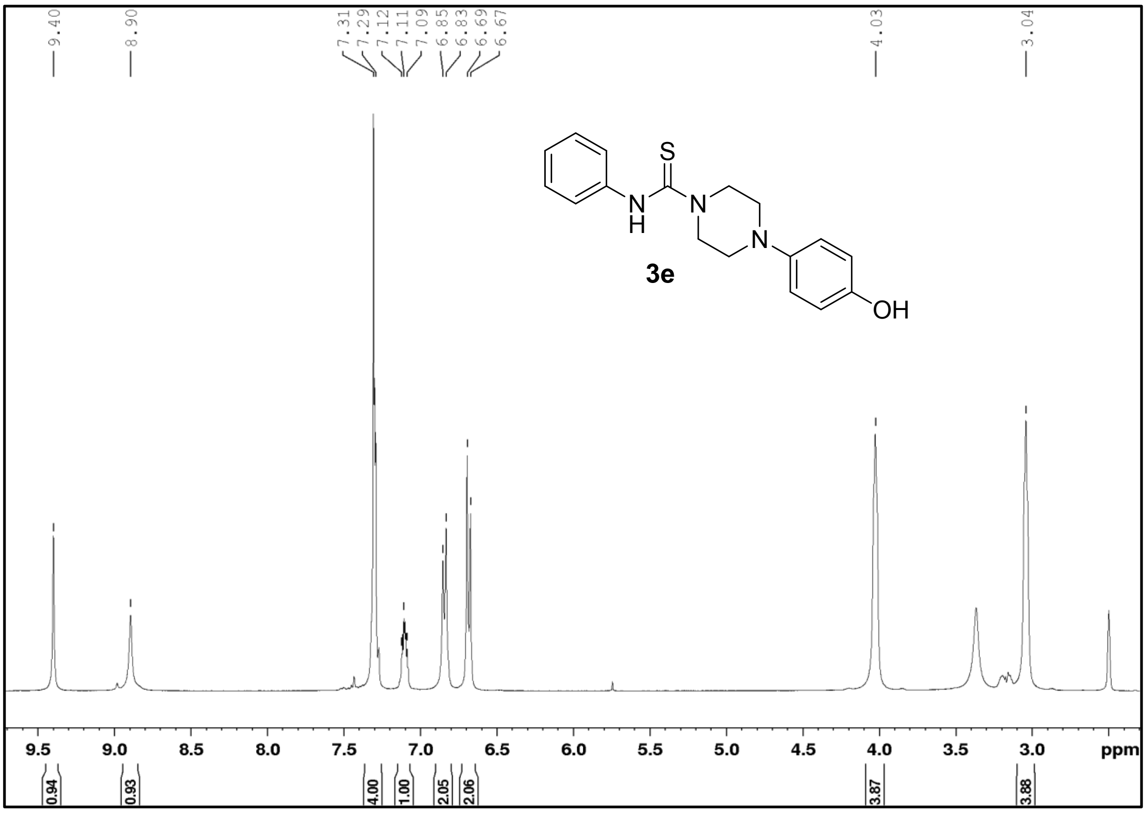

(A)

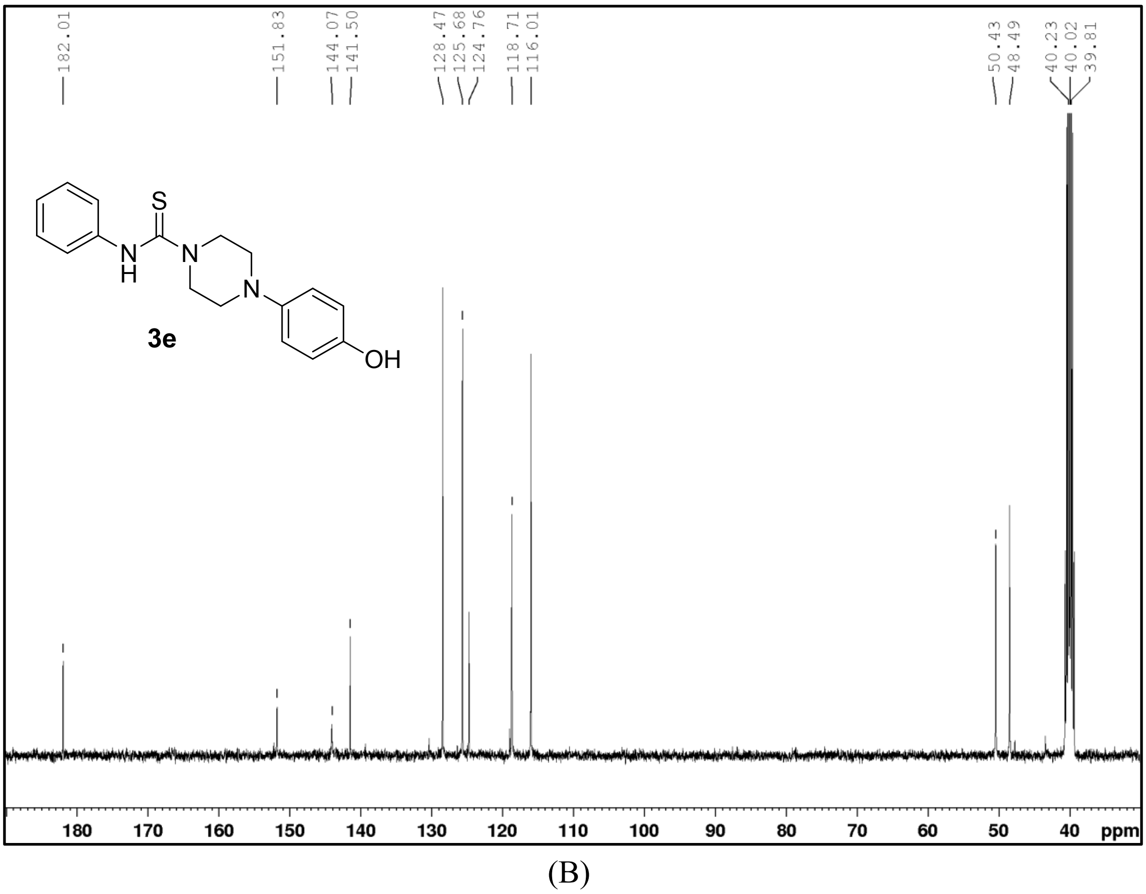

(B)

Figure S20. ^{1}H-NMR spectrum (A) and ^{13}C-NMR spectrum (B) of thiourea **3e**.

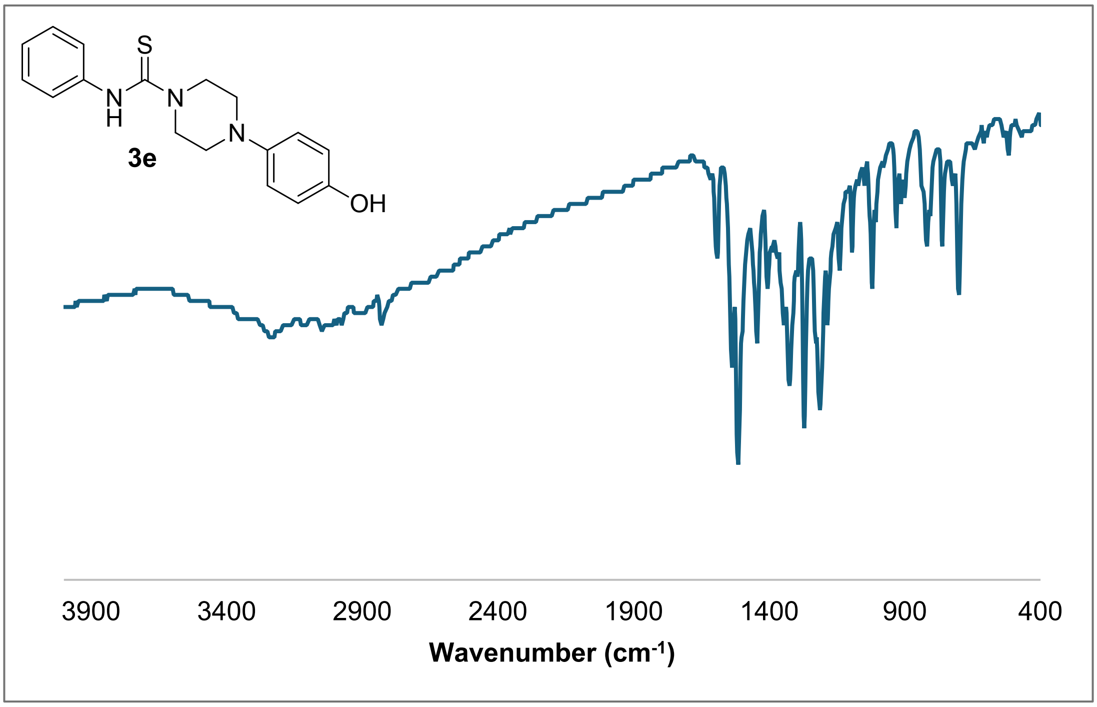

Figure S21. FT-IR spectrum of thiourea **3e**.

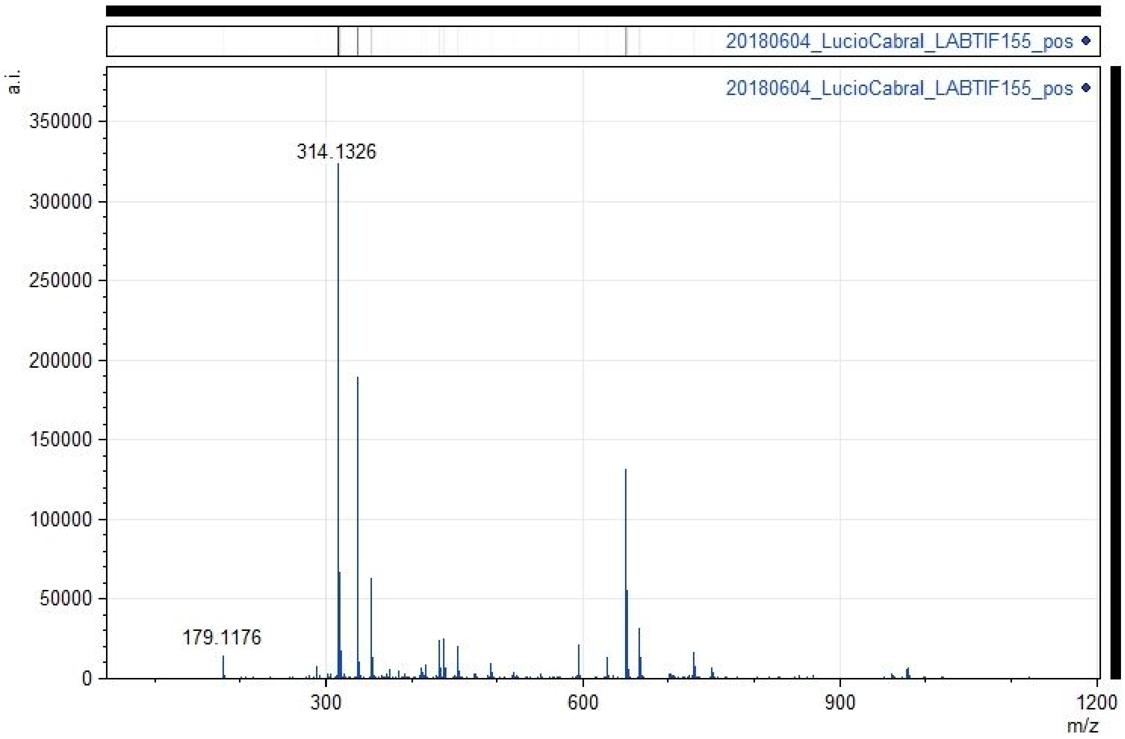

Figure S22. HR-MS spectrum of thiourea **3e**.

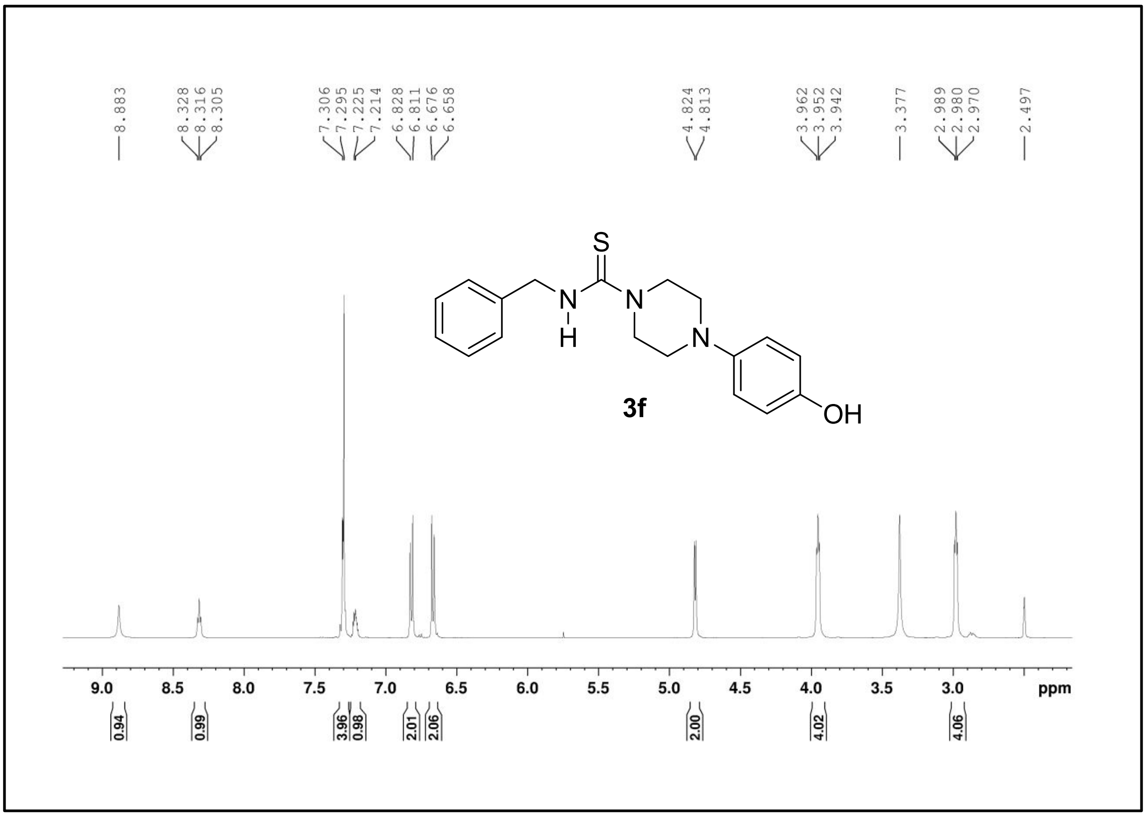

(A)

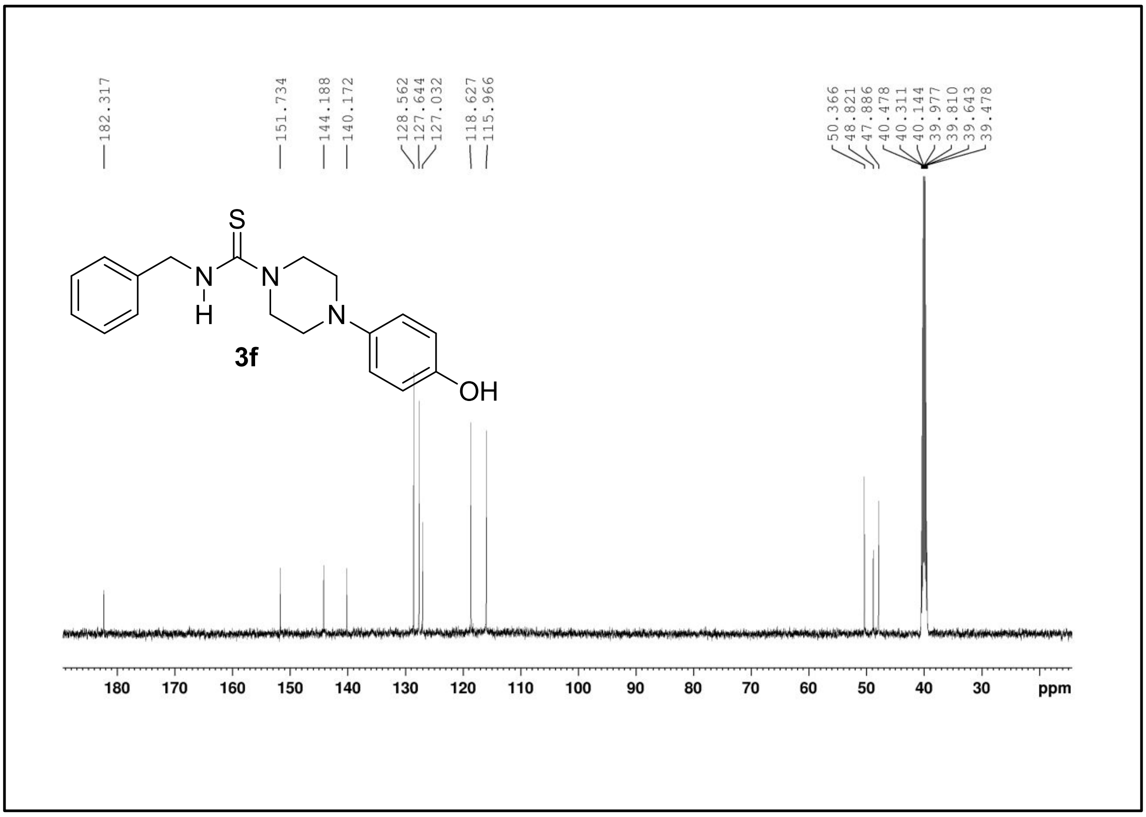

(B)

Figure S23. ^{1}H-NMR spectrum (A) and ^{13}C-NMR spectrum (B) of thiourea **3f**.

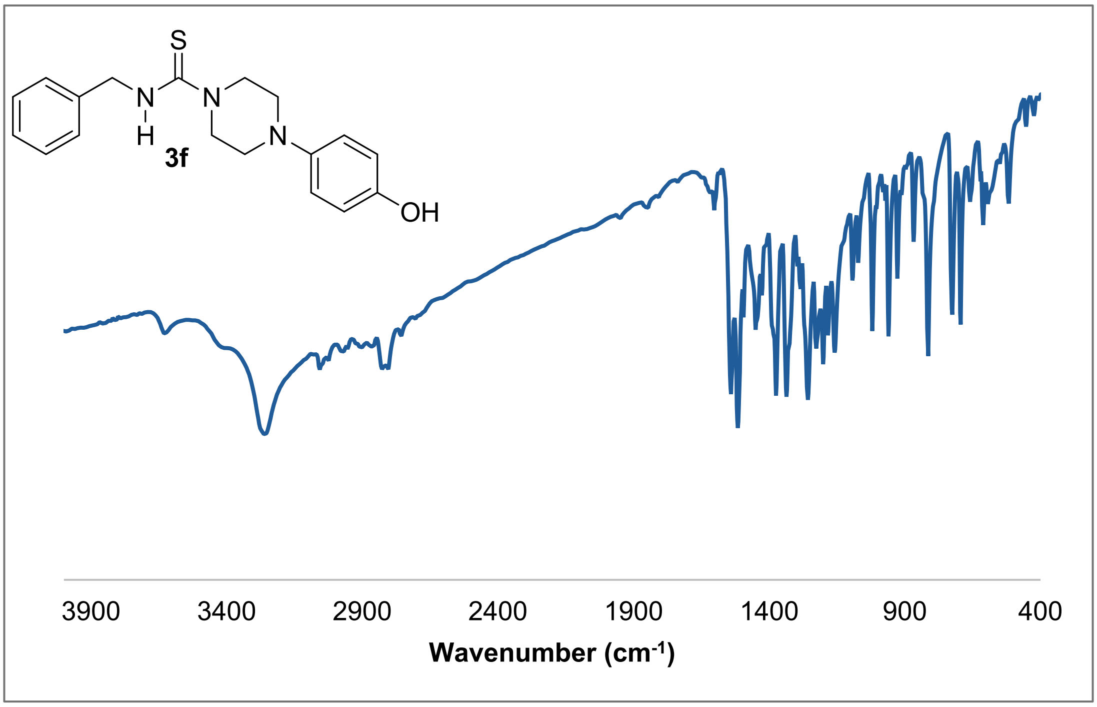

Figure S24. FT-IR spectrum of thiourea **3f**.

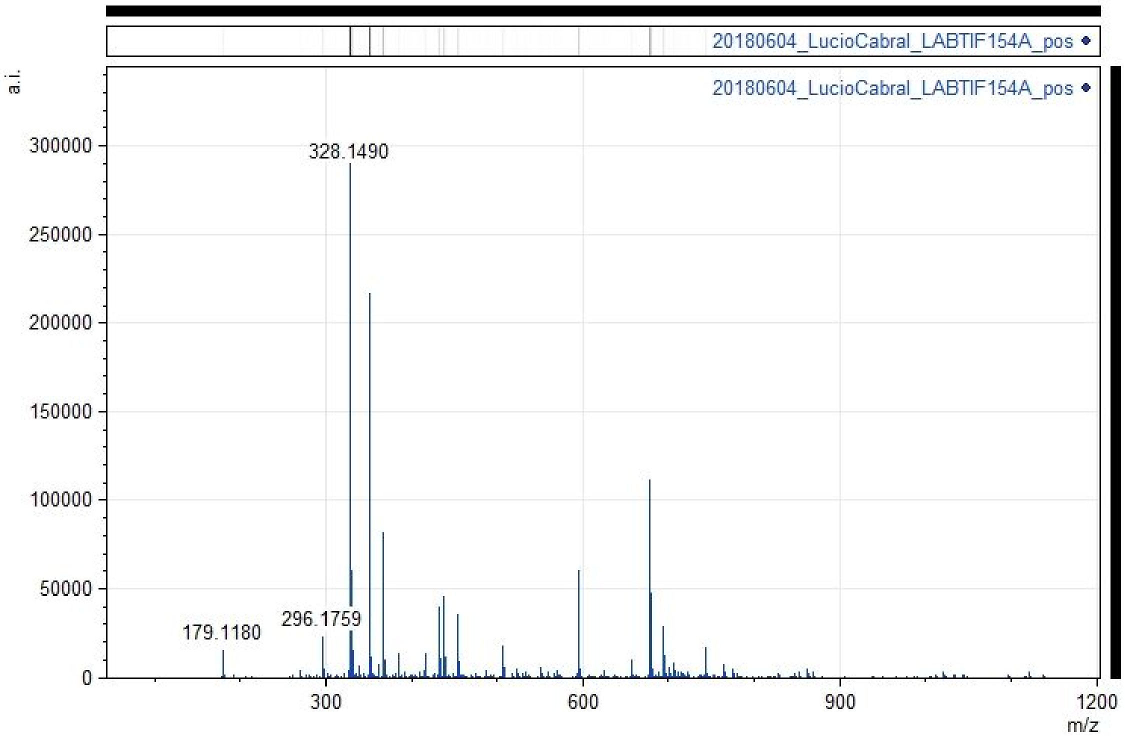

Figure S25. HR-MS spectrum of thiourea **3f**.

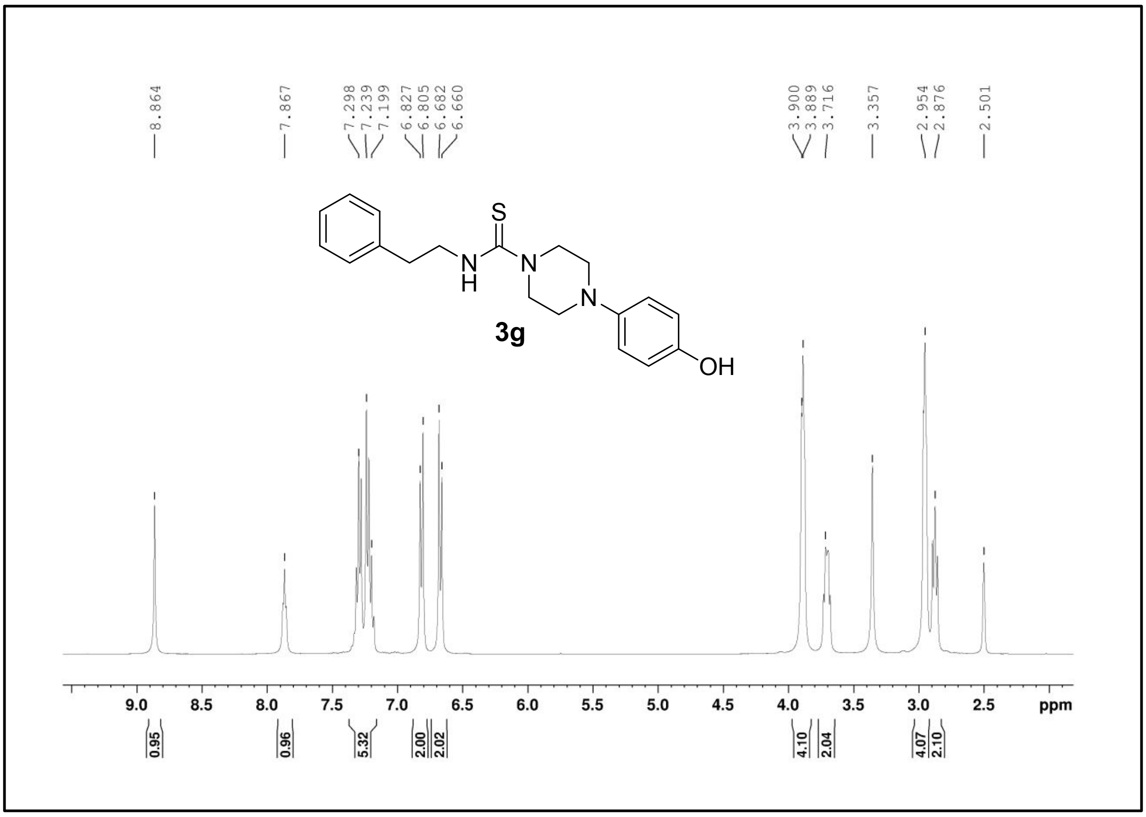

(A)

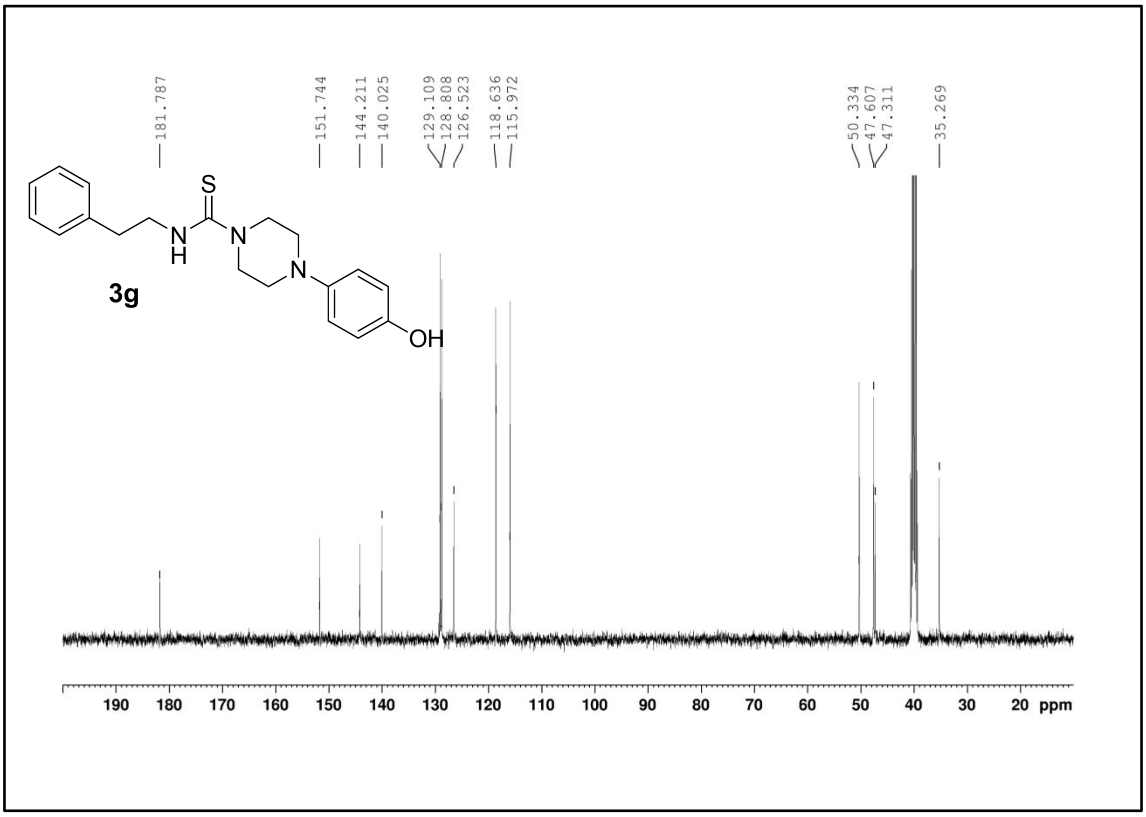

(B)

Figure S26. ^{1}H-NMR spectrum (A) and ^{13}C-NMR spectrum (B) of thiourea **3g**.

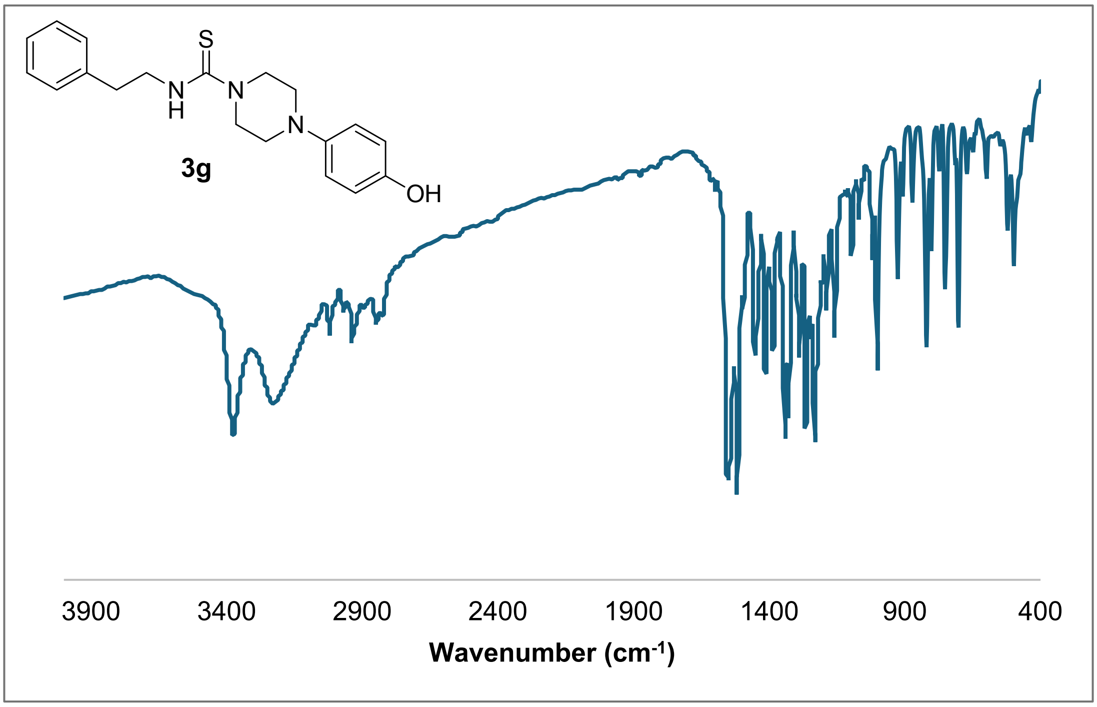

Figure S27. FT-IR spectrum of thiourea **3g**.

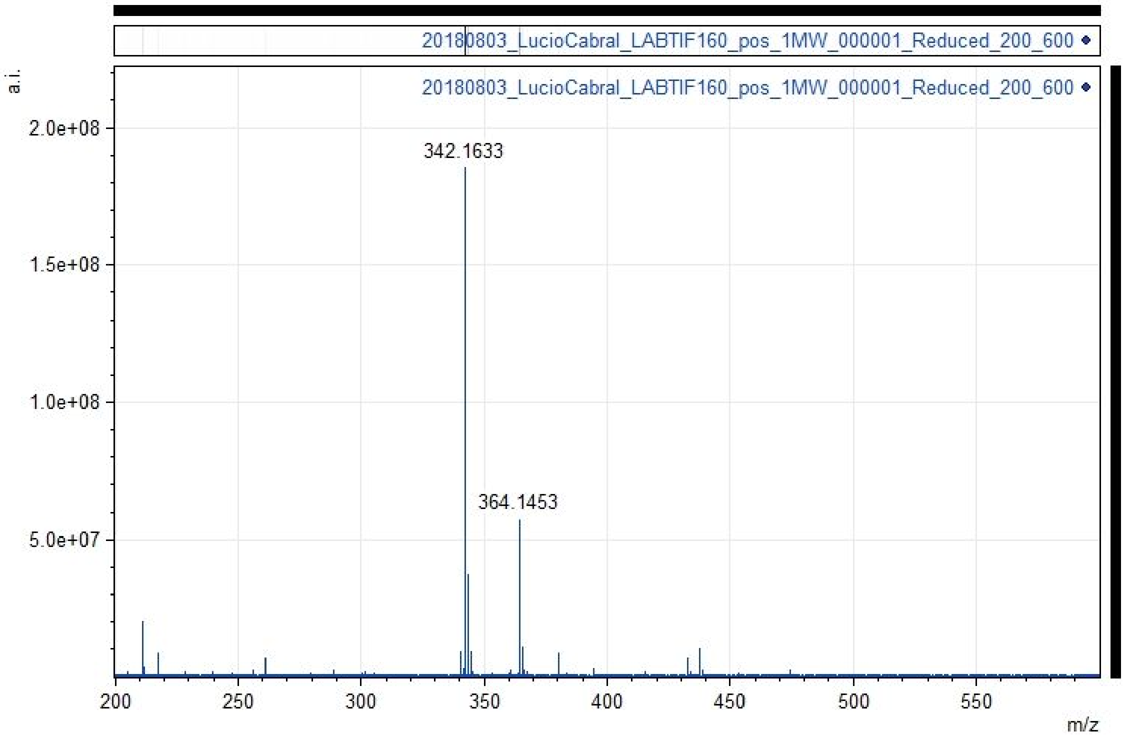

Figure S28. HR-MS spectrum of thiourea **3g**.

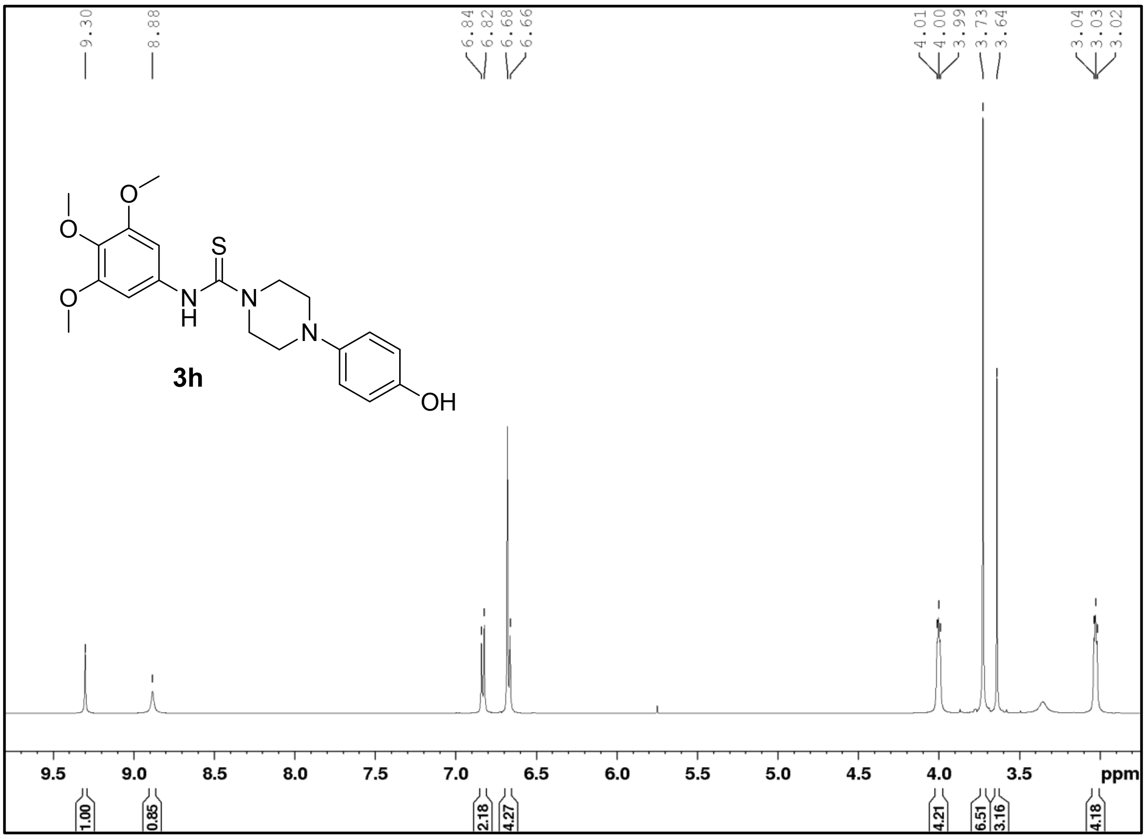

(A)

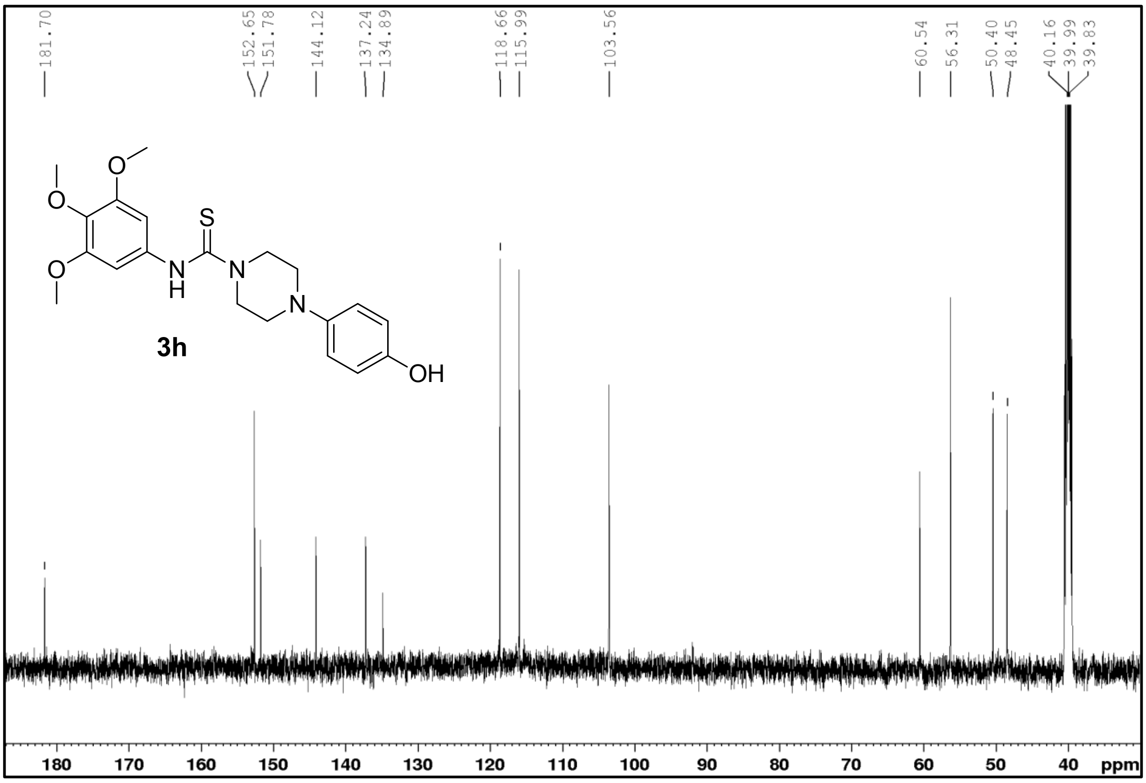

(B)

Figure S29. ^{1}H-NMR spectrum (A) and ^{13}C-NMR spectrum (B) of thiourea **3h**.

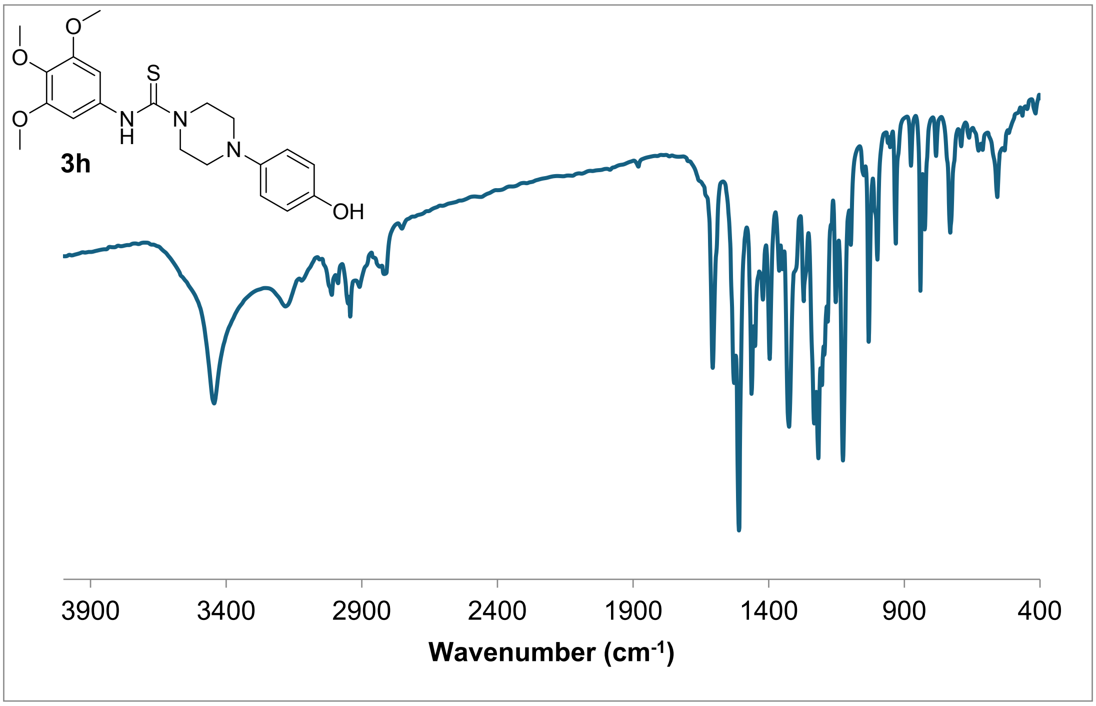

Figure S30. FT-IR spectrum of thiourea **3h**.

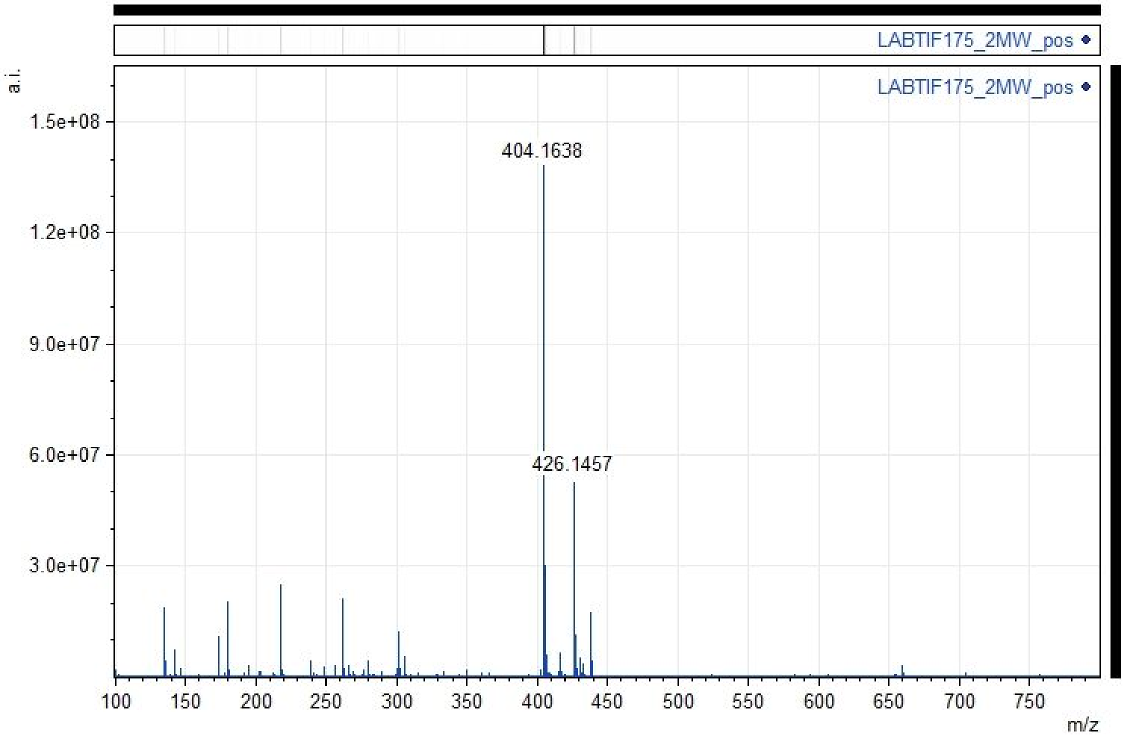

Figure S31. HR-MS spectrum of thiourea **3h**.

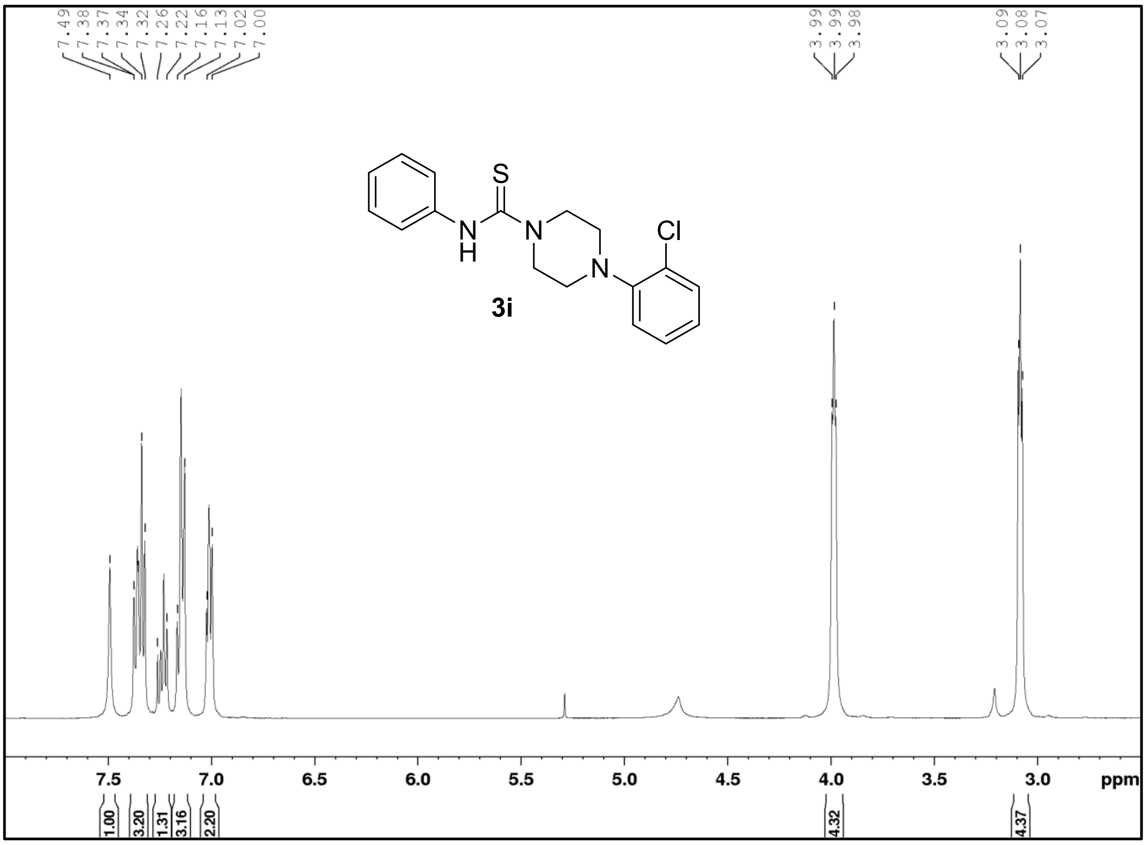

(A)

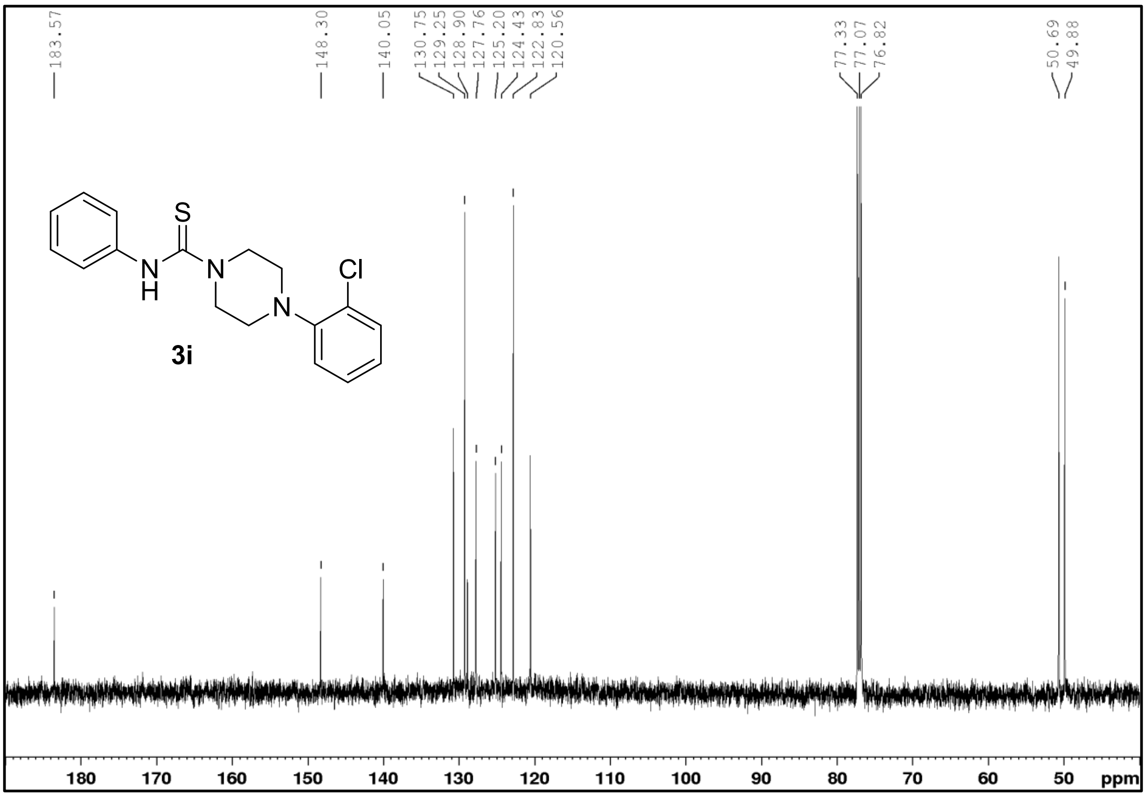

(B)

Figure S32. ^{1}H-NMR spectrum (A) and ^{13}C-NMR spectrum (B) of thiourea **3i**.

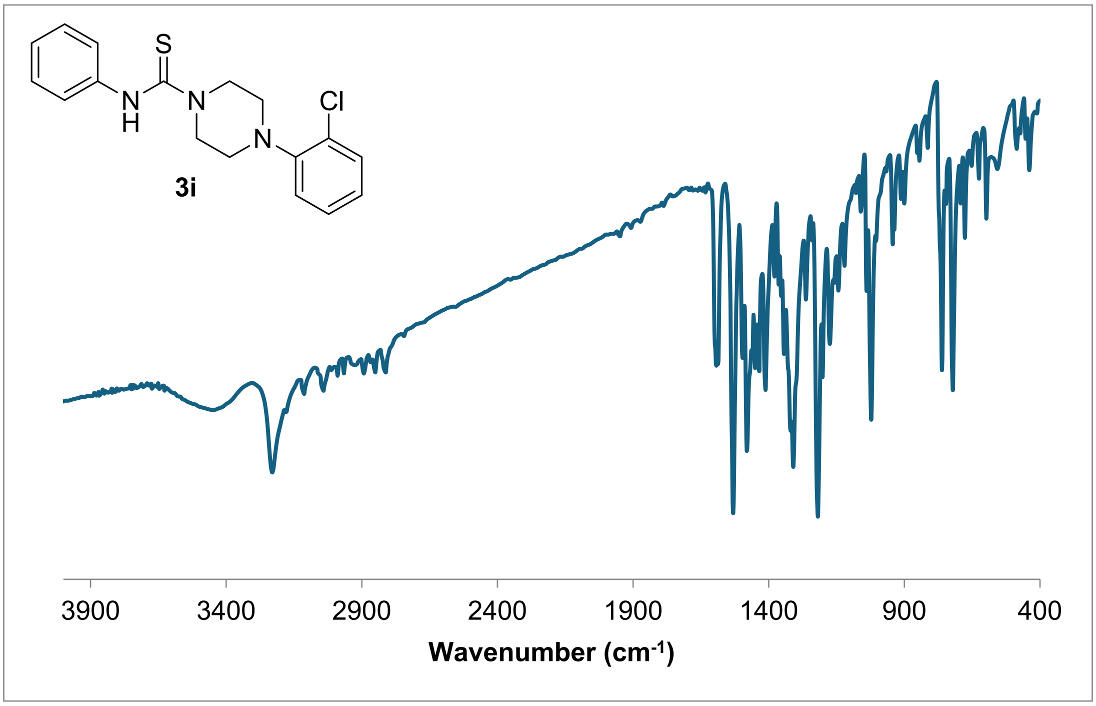

Figure S33. FT-IR spectrum of thiourea **3i**.

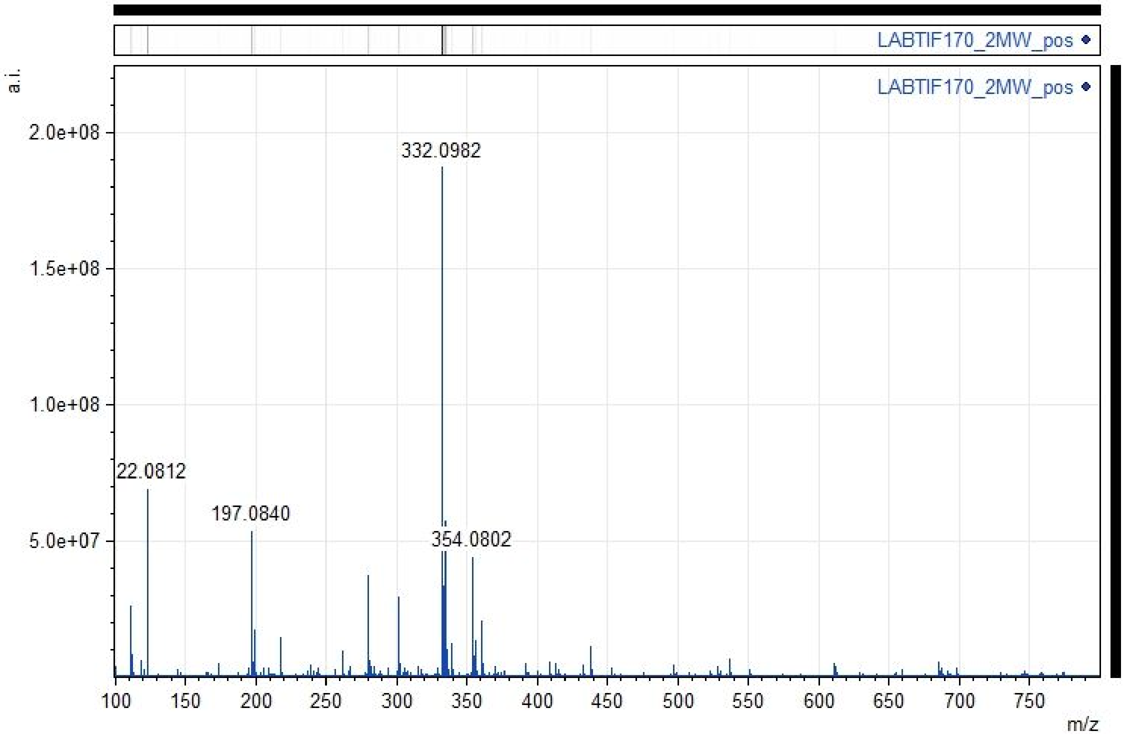

Figure S34. HR-MS spectrum of thiourea **3i**.

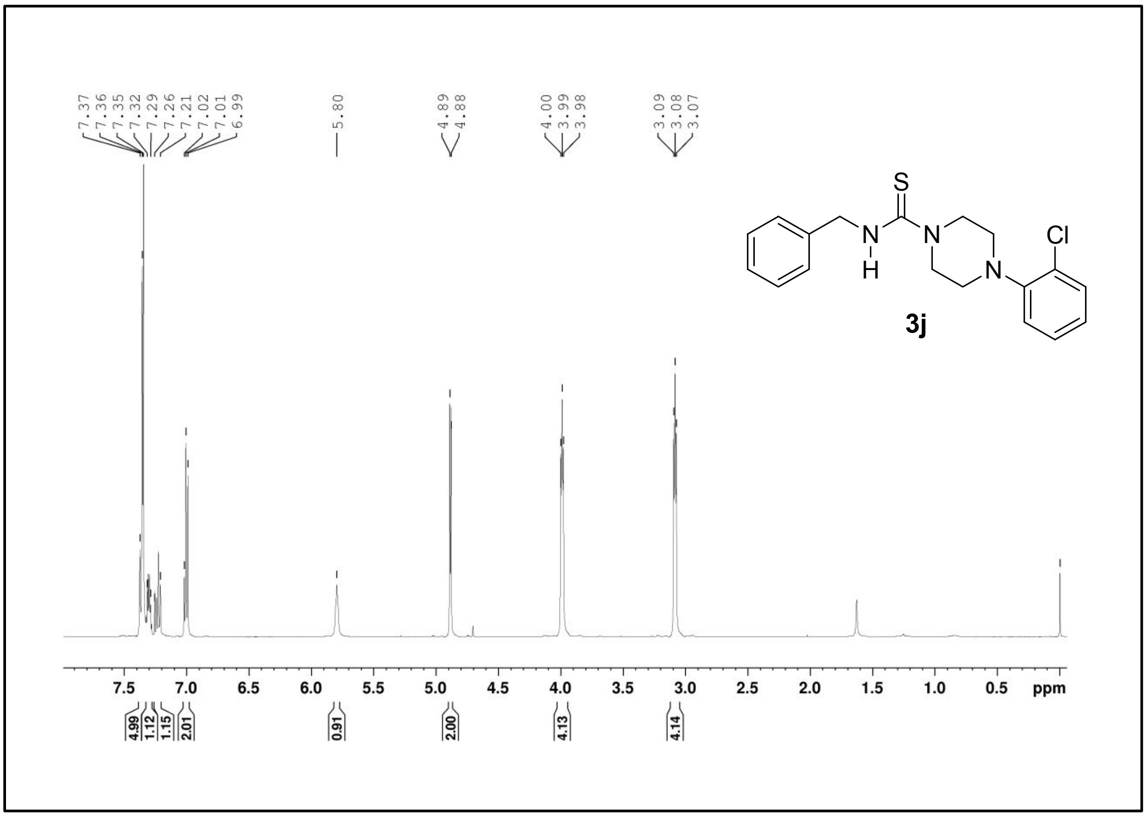

(A)

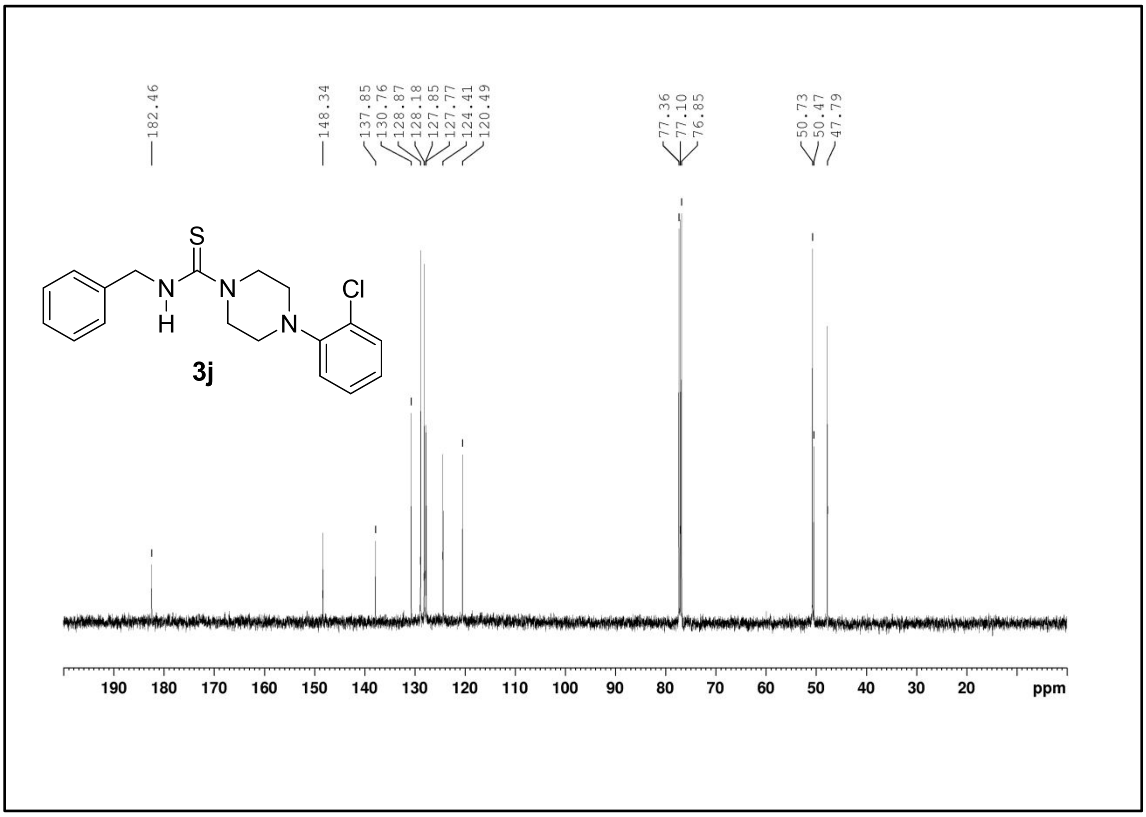

(B)

Figure S35. ^{1}H-NMR spectrum (A) and ^{13}C-NMR spectrum (B) of thiourea **3j**.

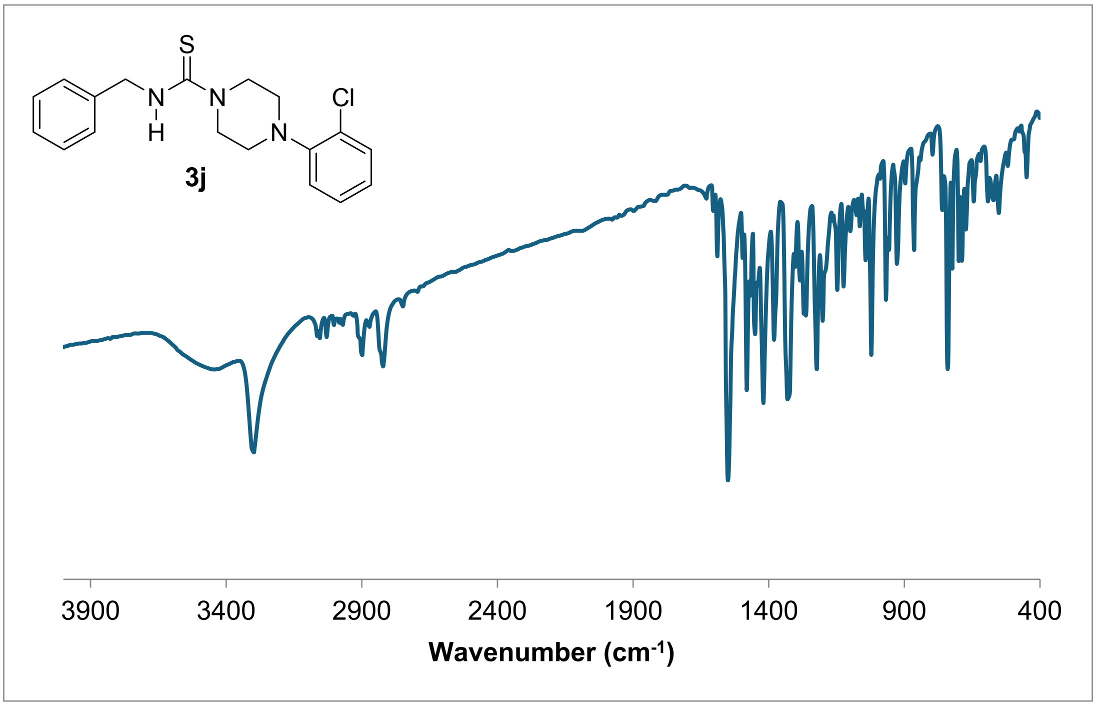

Figure S36. FT-IR spectrum of thiourea **3j**.

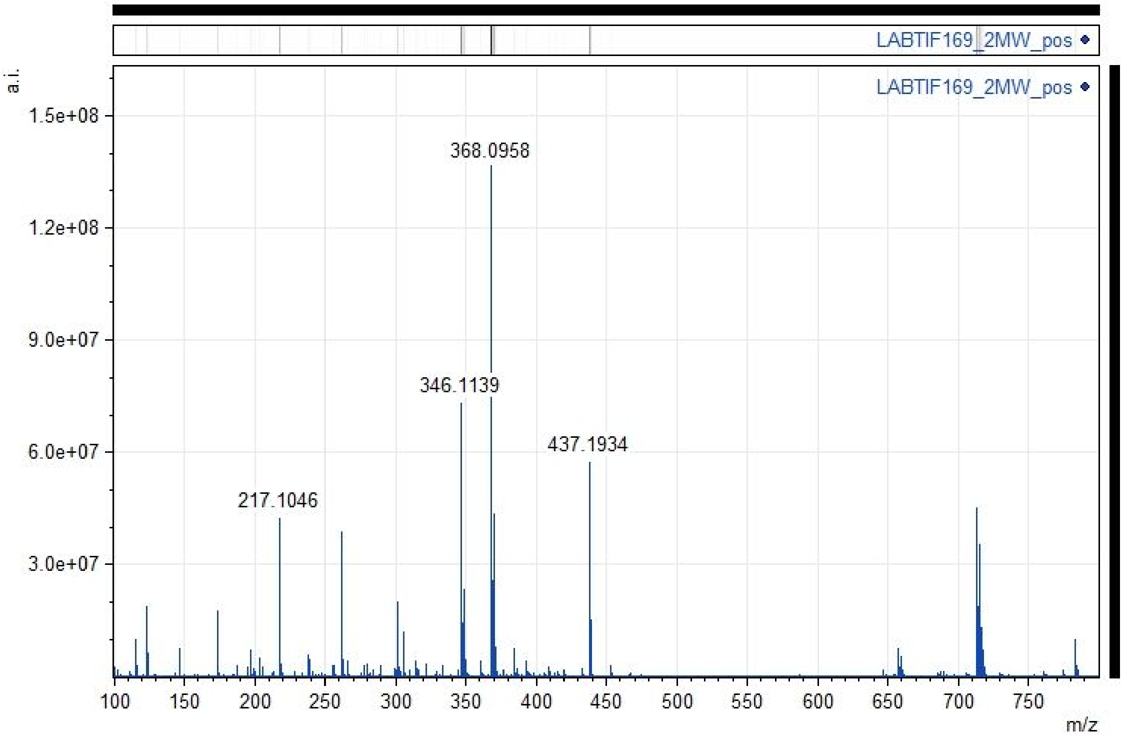

Figure S37. HR-MS spectrum of thiourea **3j**.

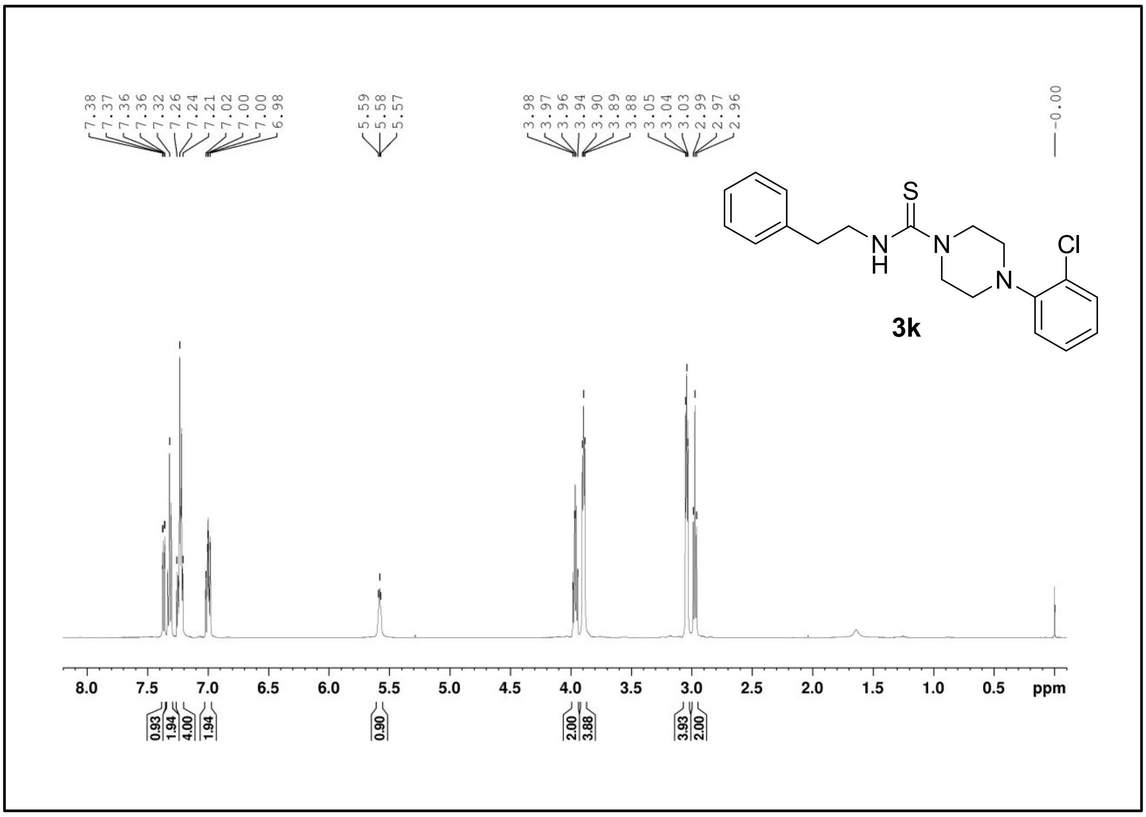

(A)

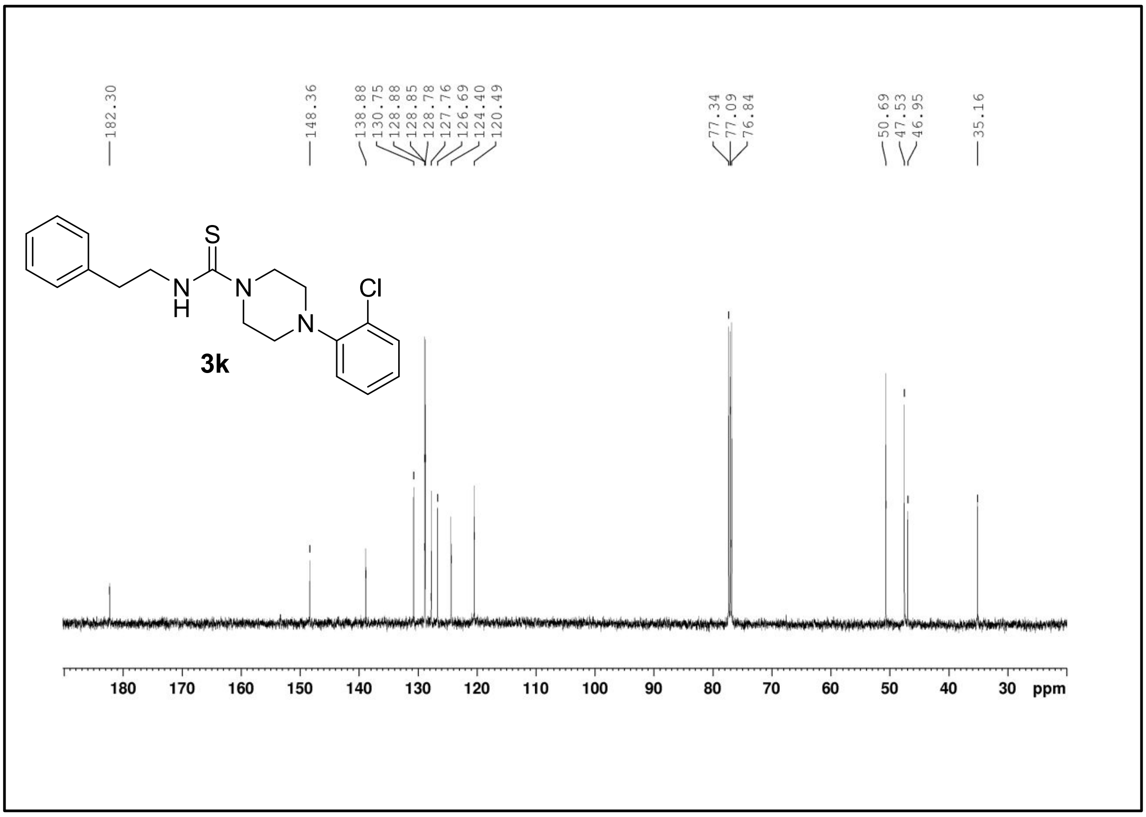

(B)

Figure S38. ^{1}H-NMR spectrum (A) and ^{13}C-NMR spectrum (B) of thiourea **3k**.

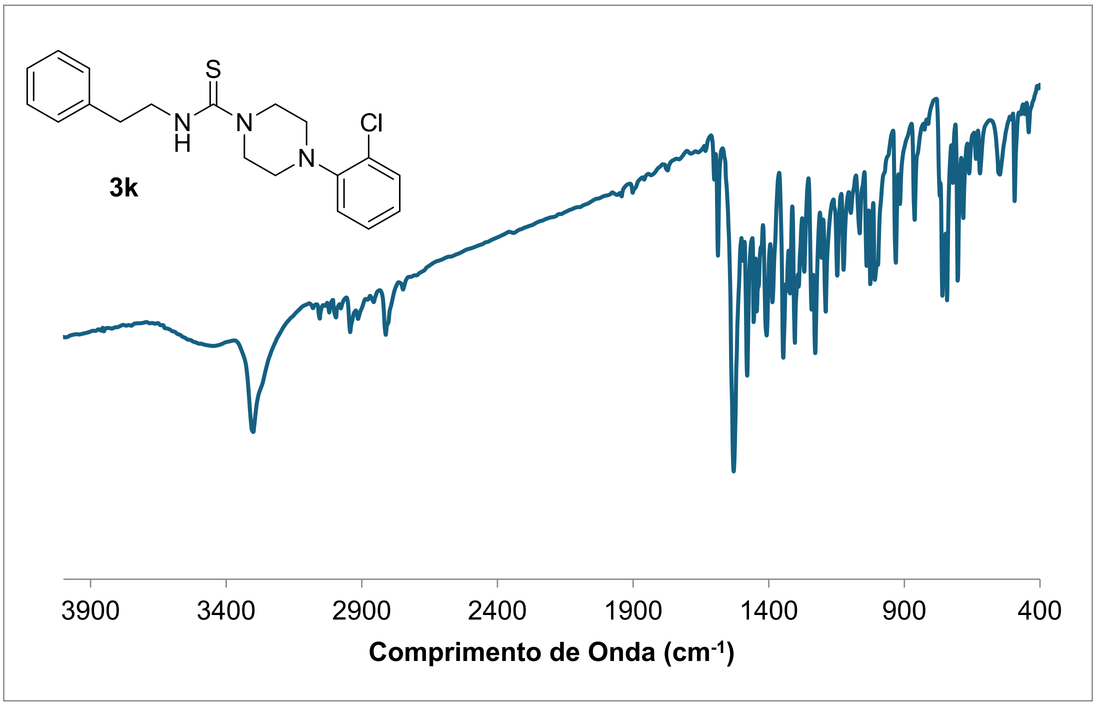

Figure S39. FT-IR spectrum of thiourea **3k**.

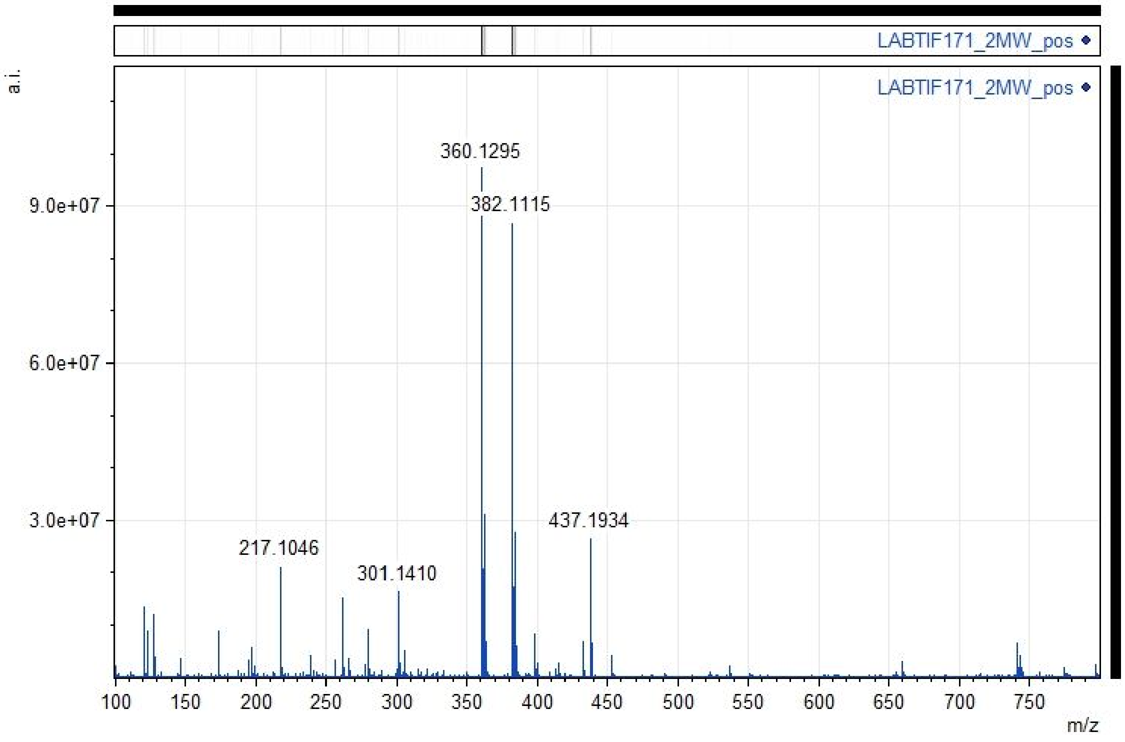

Figure S40. HR-MS spectrum of thiourea **3k**.

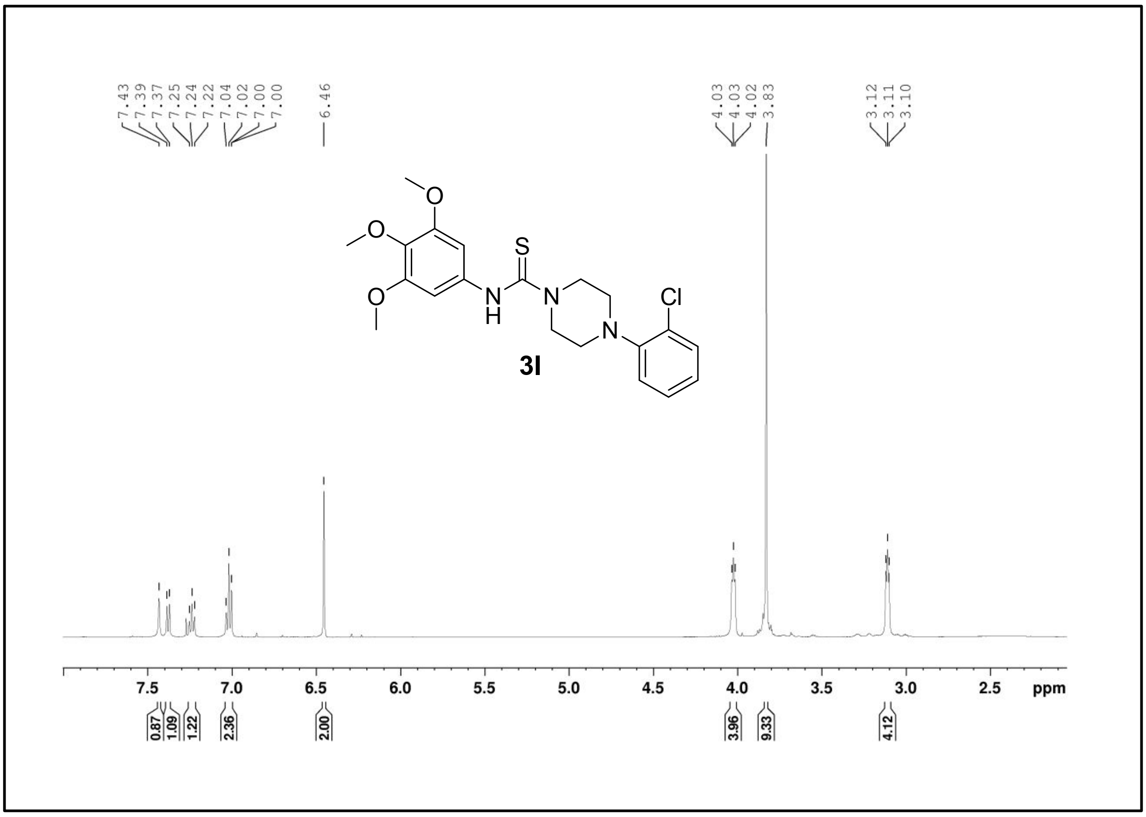

(A)

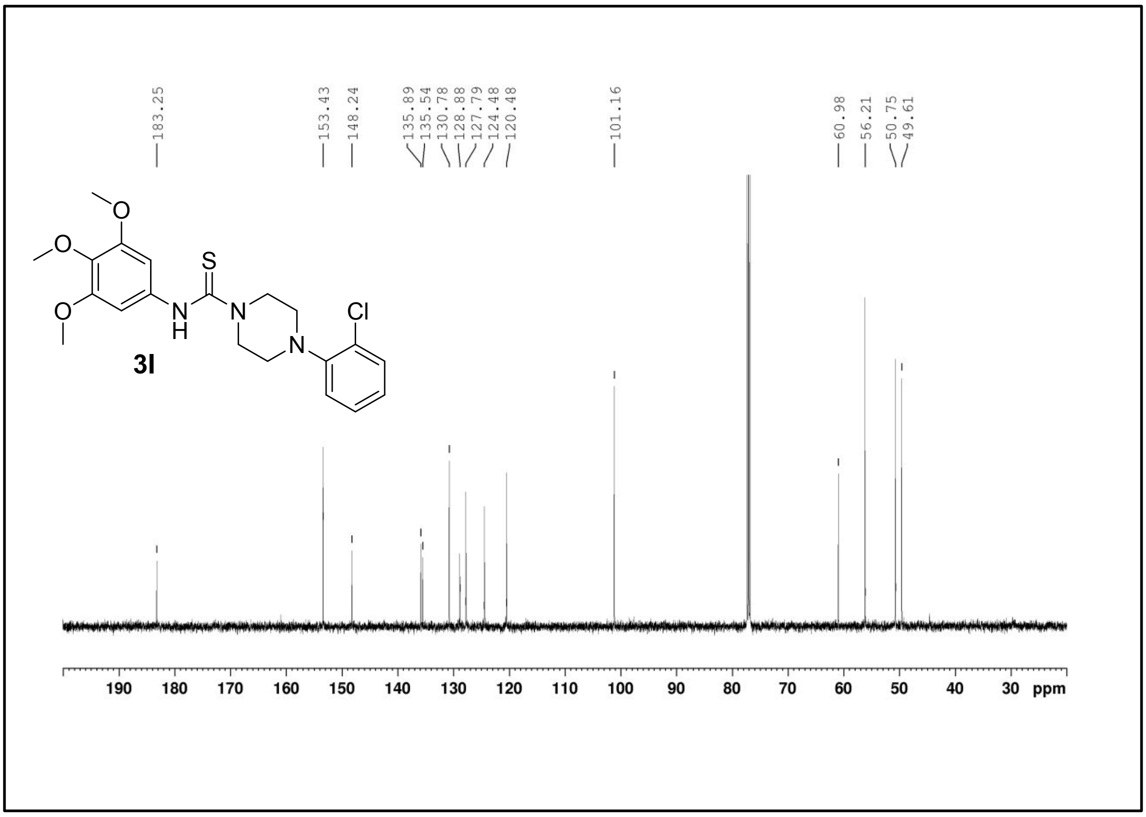

(B)

Figure S41. ^{1}H-NMR spectrum (A) and ^{13}C-NMR spectrum (B) of thiourea **3l**.

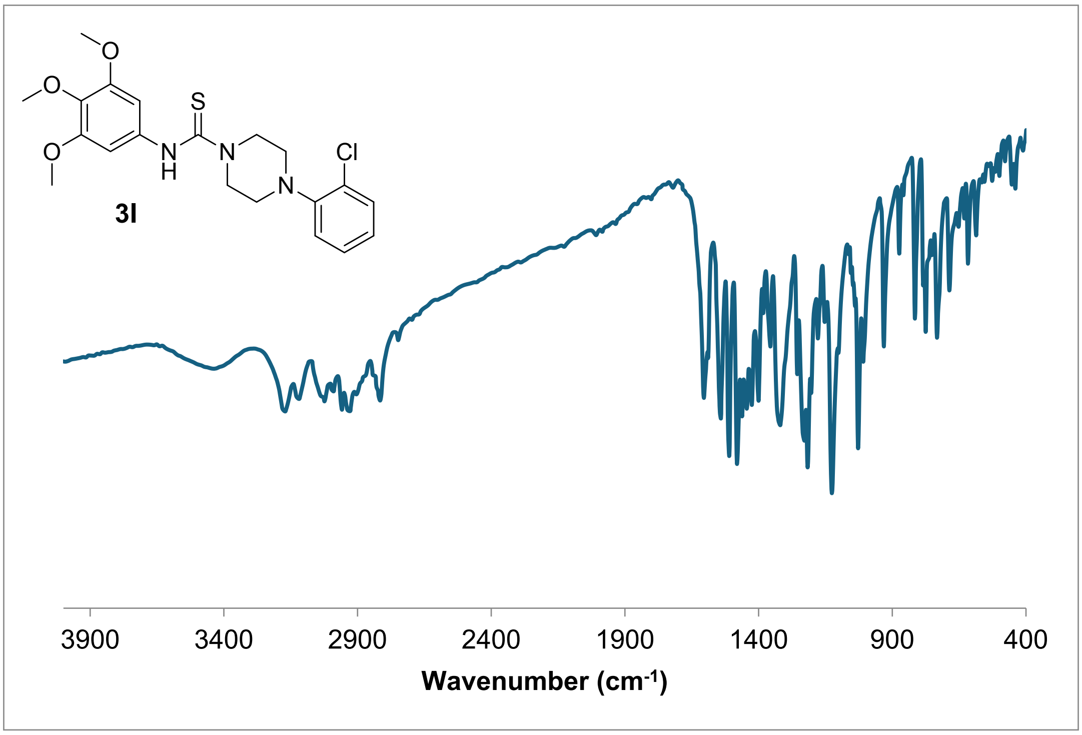

Figure S42. FT-IR spectrum of thiourea **3l**.

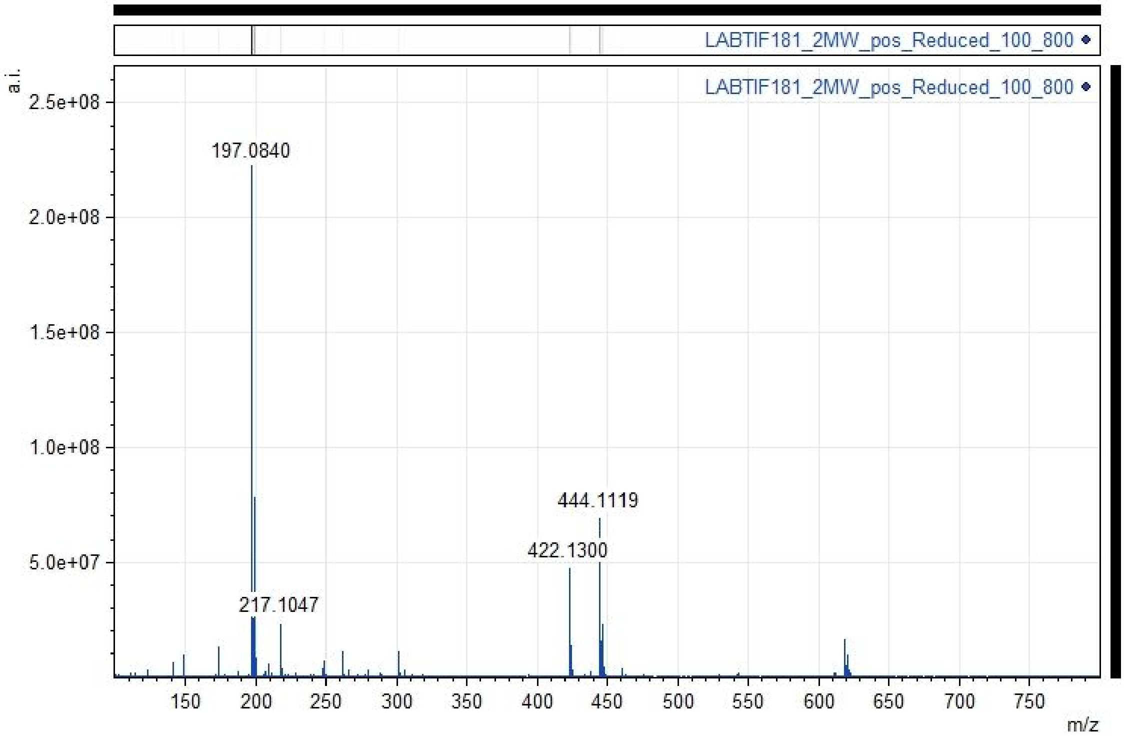

Figure S43. HR-MS spectrum of thiourea **3l**.

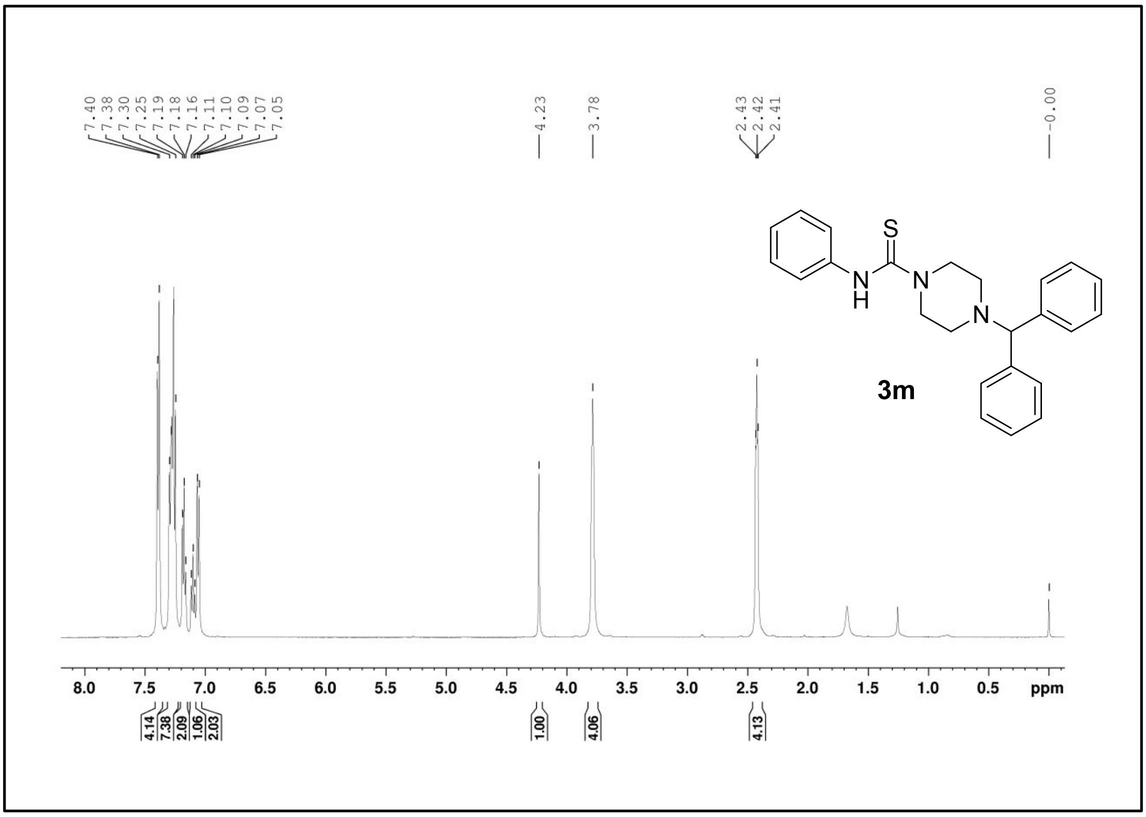

(A)

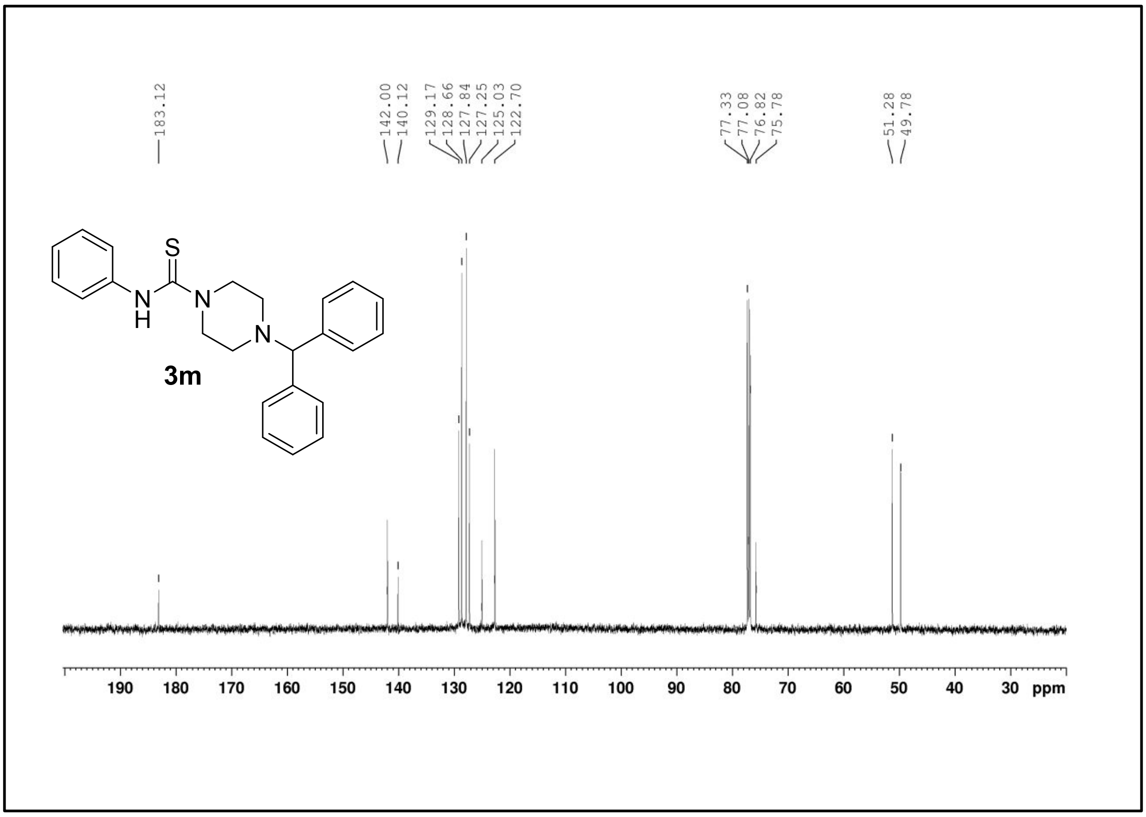

(B)

Figure S44. ^{1}H-NMR spectrum (A) and ^{13}C-NMR spectrum (B) of thiourea **3m**.

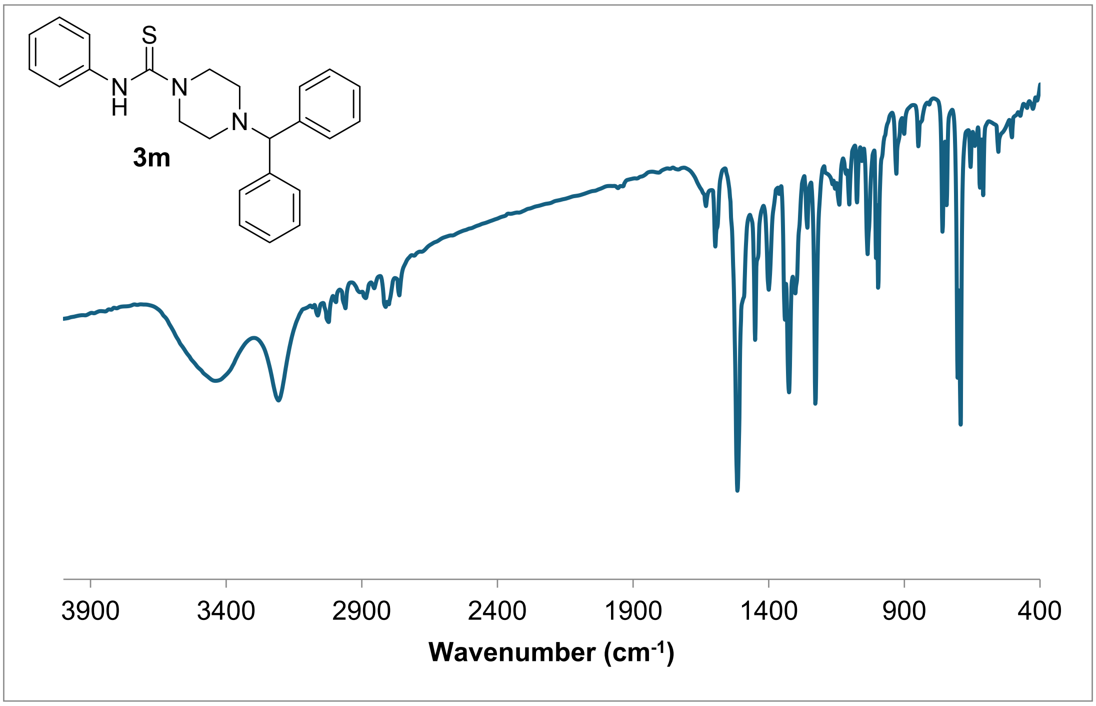

Figure S45. FT-IR spectrum of thiourea **3m**.

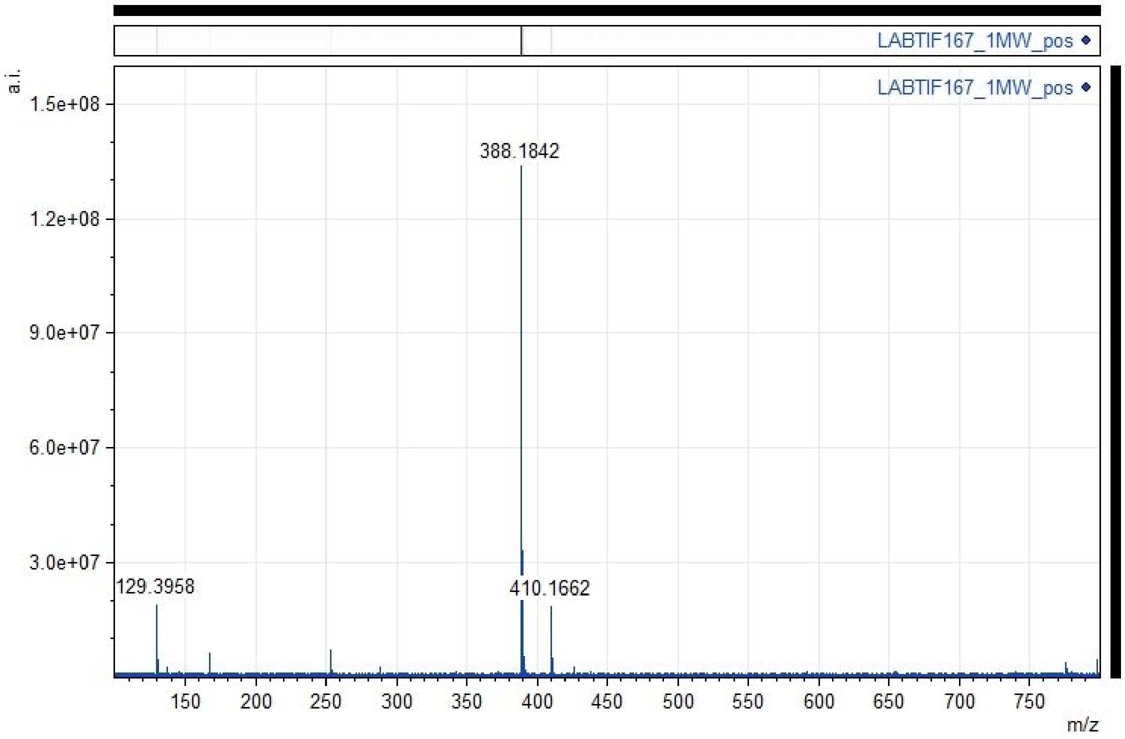

Figure S46. HR-MS spectrum of thiourea **3m**.

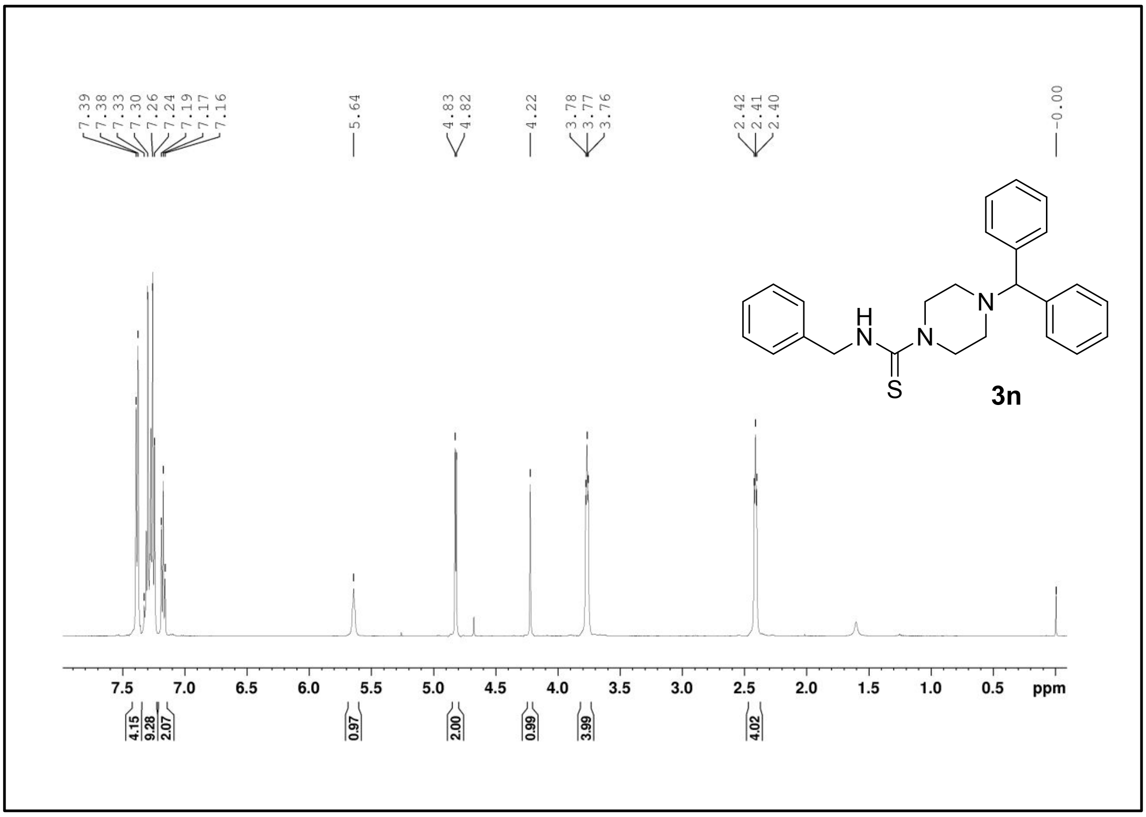

(A)

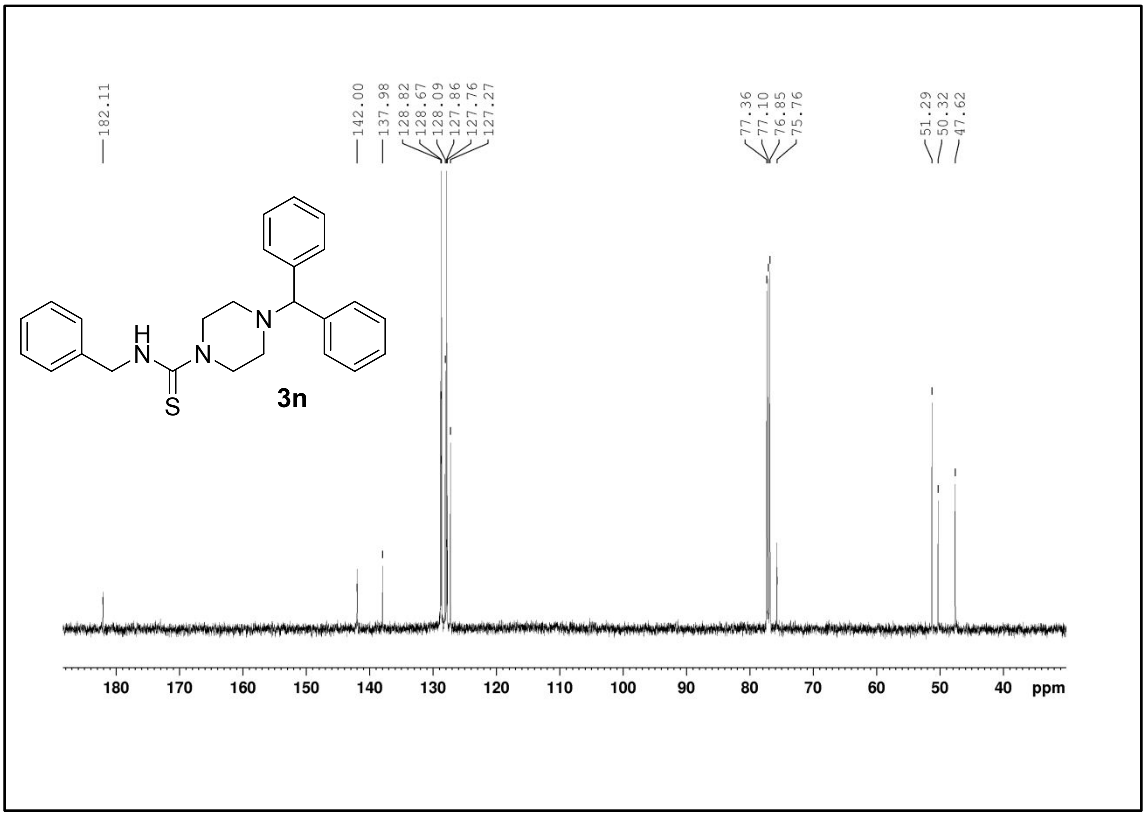

(B)

Figure S47. ^{1}H-NMR spectrum (A) and ^{13}C-NMR spectrum (B) of thiourea **3n**.

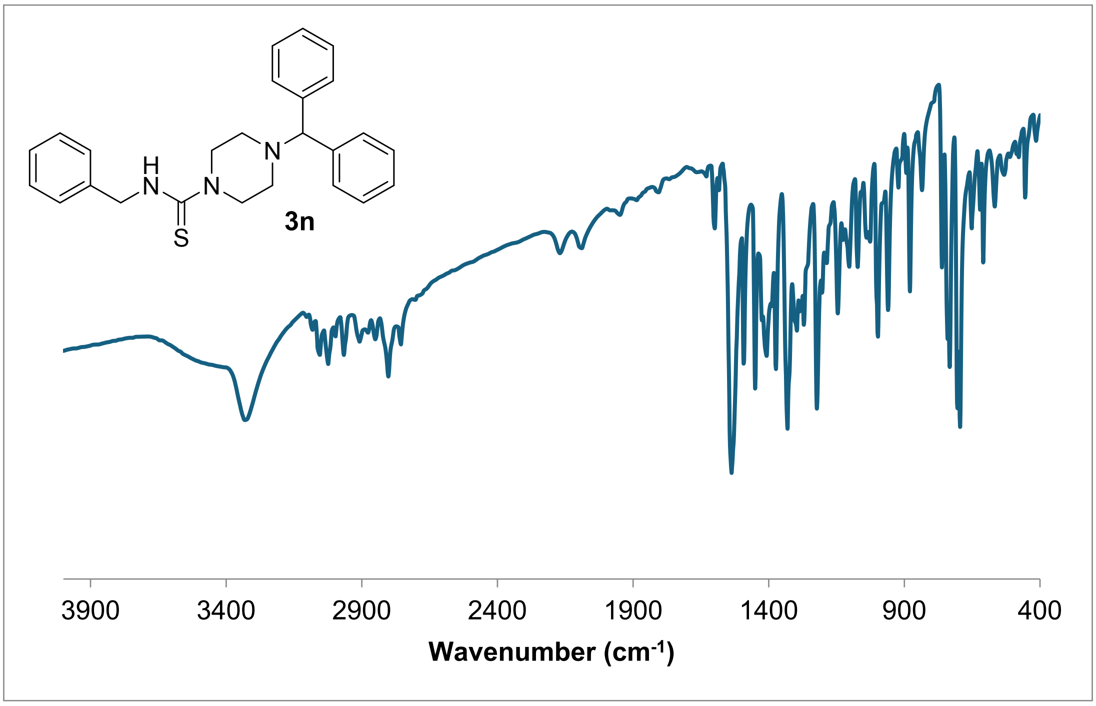

Figure S48. FT-IR spectrum of thiourea **3n**.

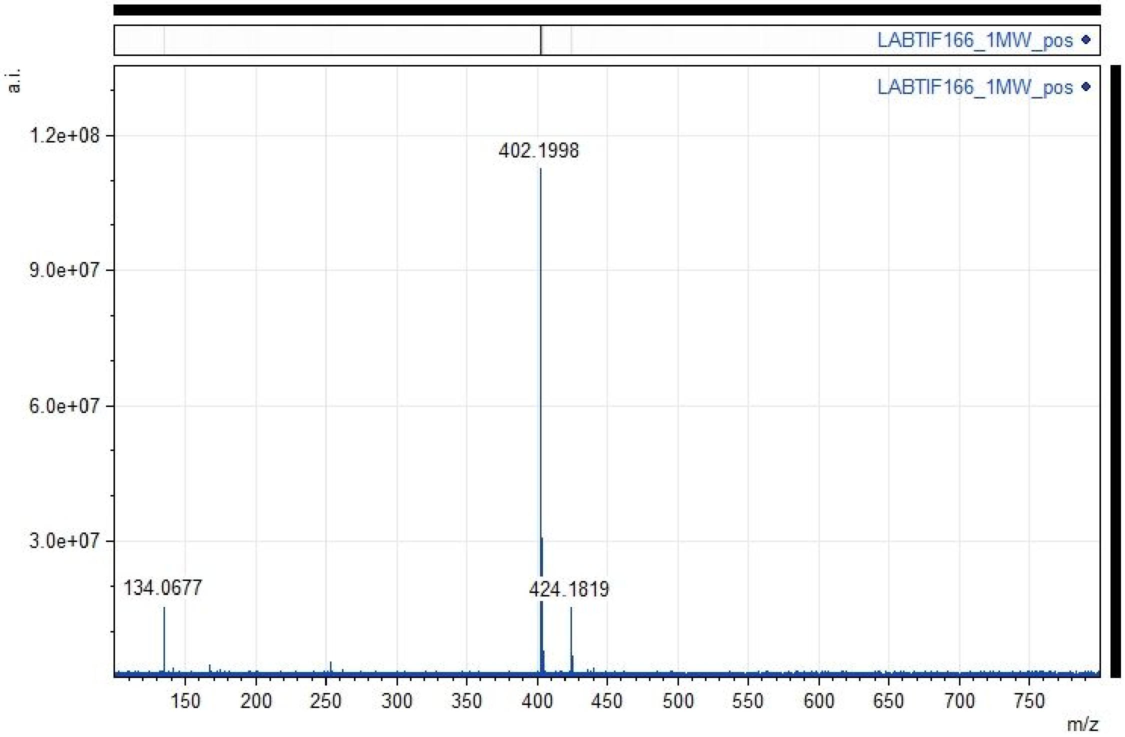

Figure S49. HR-MS spectrum of thiourea **3n**.

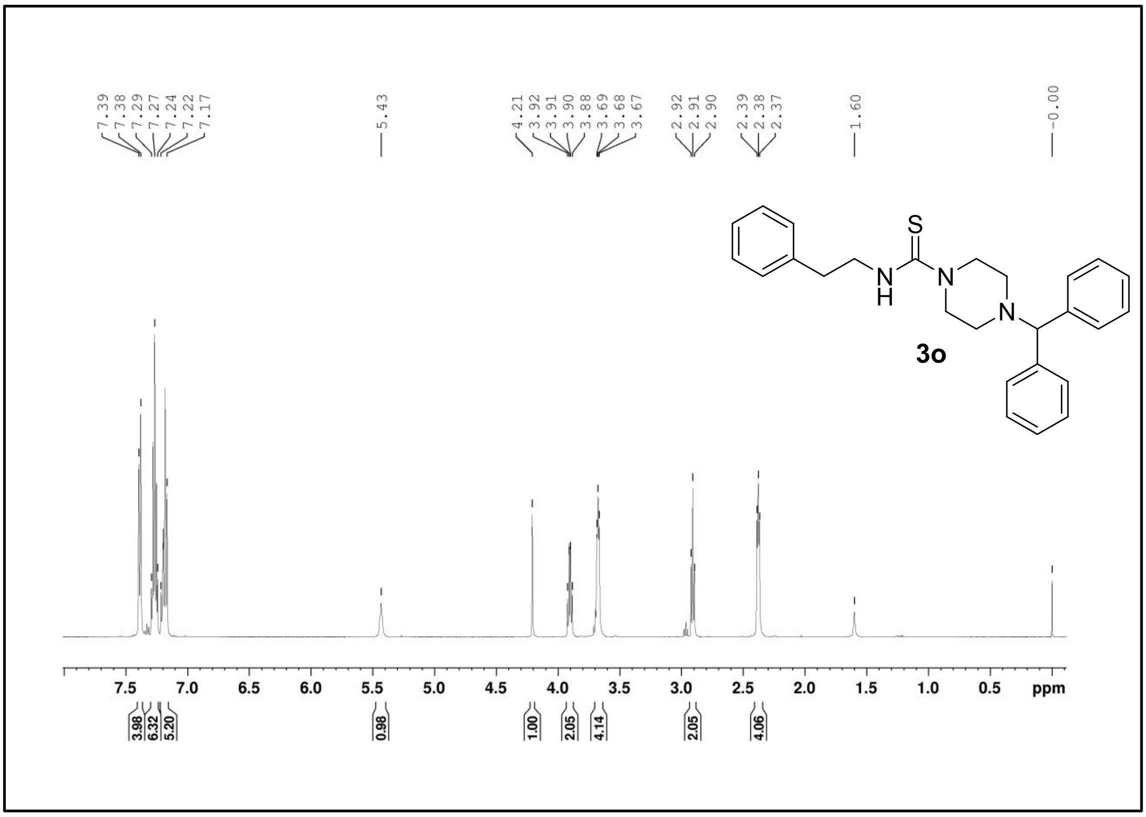

(A)

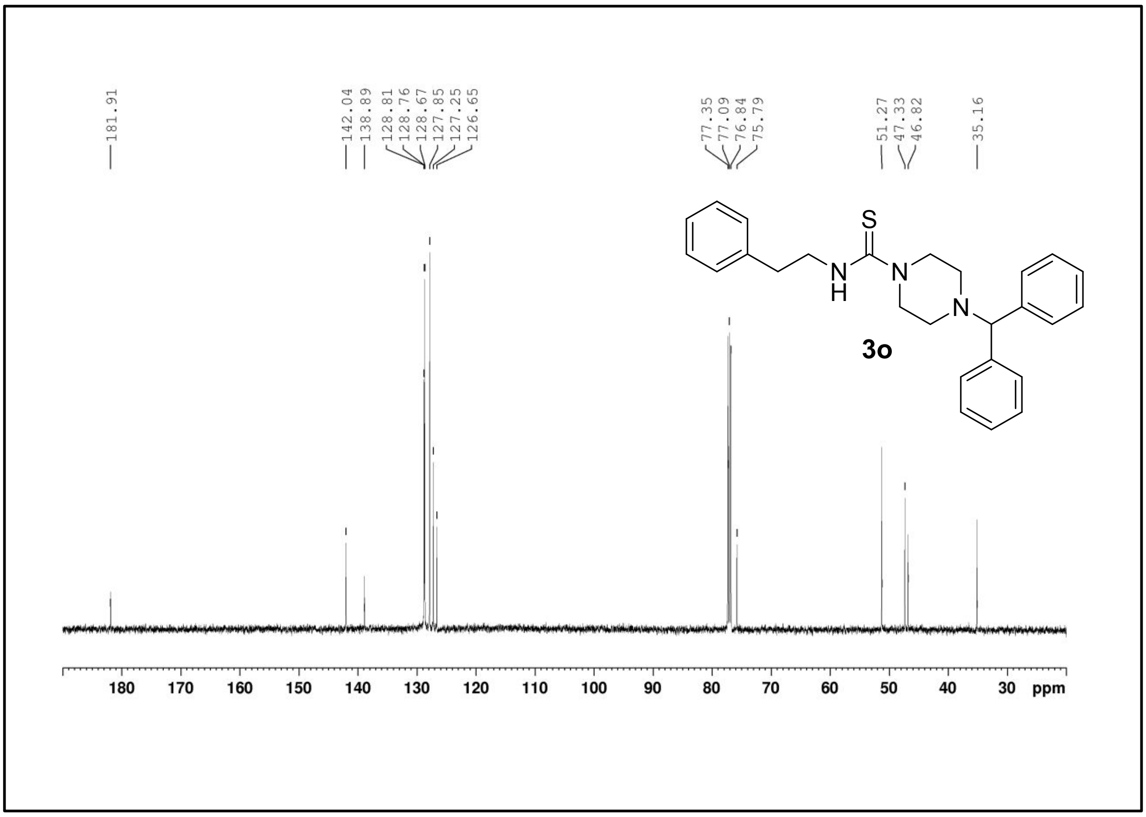

(B)

Figure S50. ^{1}H-NMR spectrum (A) and ^{13}C-NMR spectrum (B) of thiourea **3o**.

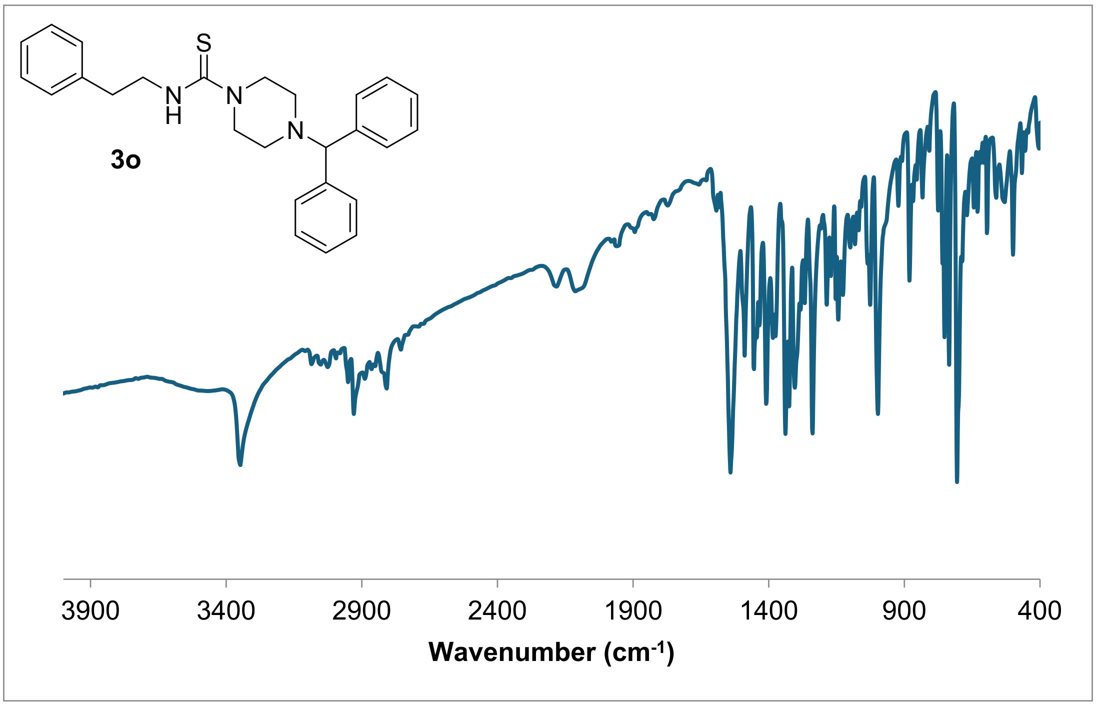

Figure S51. FT-IR spectrum of thiourea **3o**.

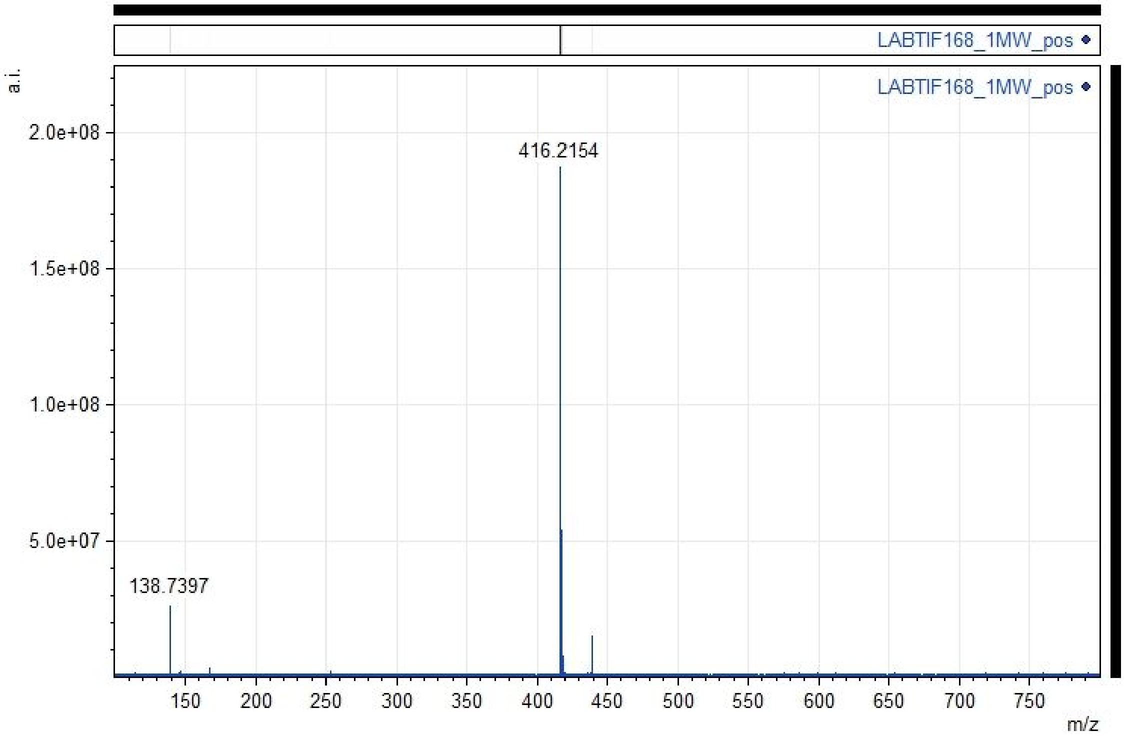

Figure S52. HR-MS spectrum of thiourea **3o**.

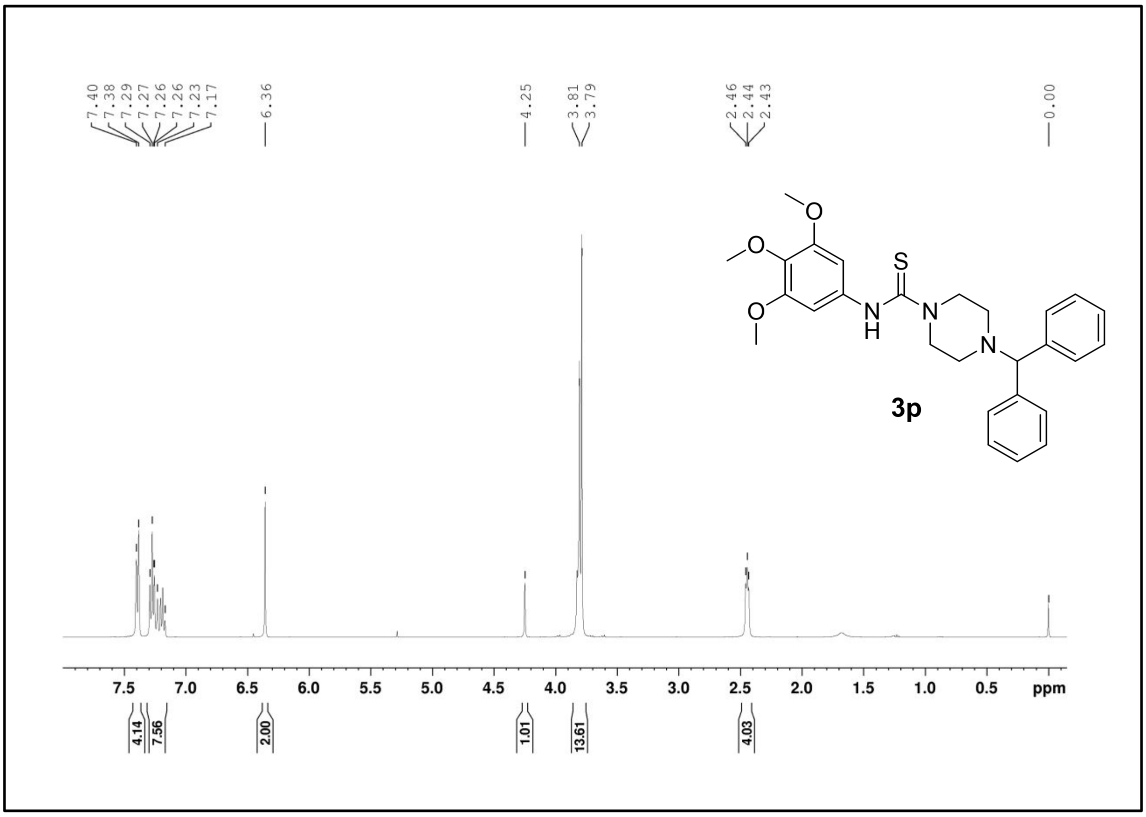

(A)

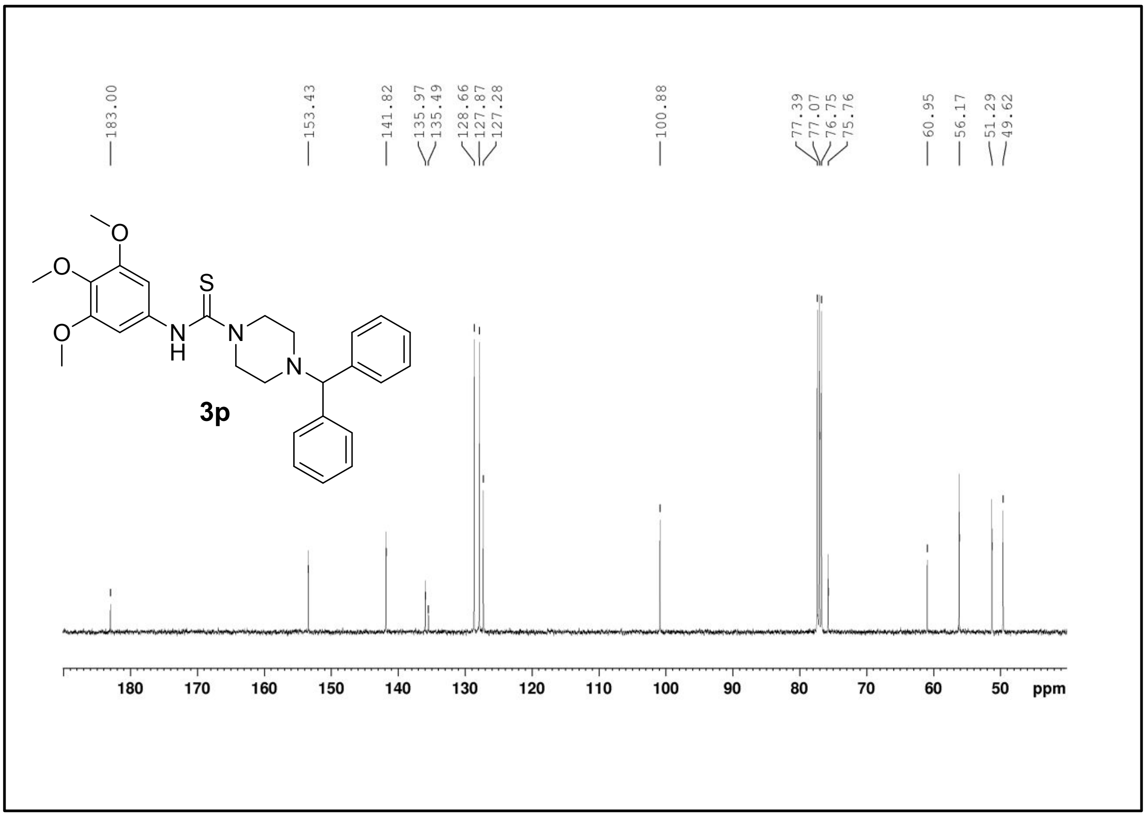

(B)

Figure S53. ^{1}H-NMR spectrum (A) and ^{13}C-NMR spectrum (B) of thiourea **3p**.

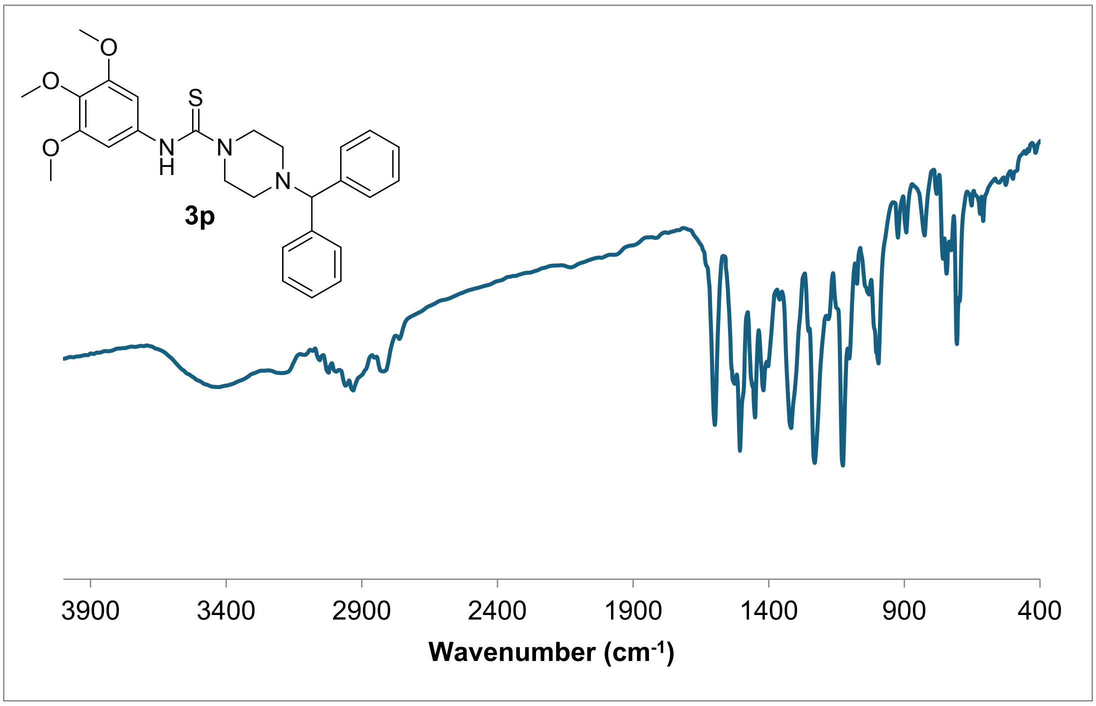

Figure S54. FT-IR spectrum of thiourea **3p**.

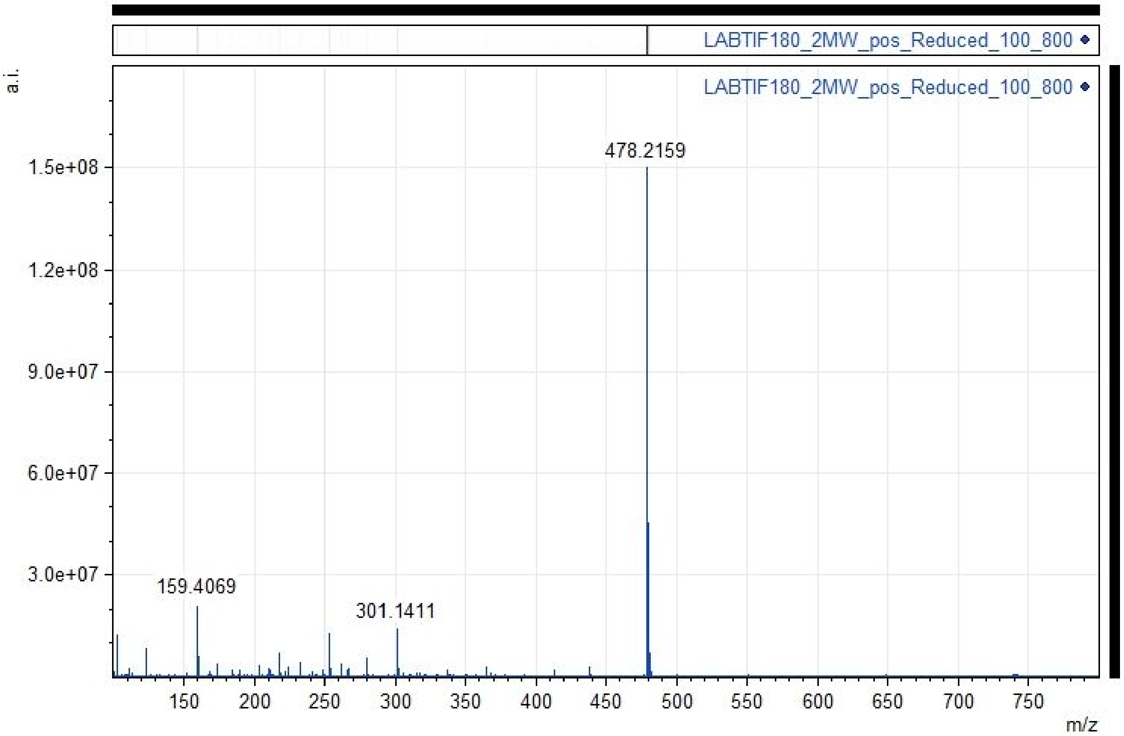

Figure S55. HR-MS spectrum of thiourea **3p**.

In Vitro **Biological Assays and Antiplatelet Activity Profiles**

Table S1. Antiplatelet profile of piperazine thiourea derivatives (100 µM) on platelet aggregation induced by arachidonic acid (AA) (500 µM). Acetylsalicylic acid (ASA) was used as a positive control and DMSO (1%) as a negative control. Results are expressed as mean ± standard deviation (SD) (n=3). *$p \leq 0.05$ (one-way ANOVA, Tukey's test).*

Compound	Aggregation (%)	Inhibition (%)
**DMSO 1%**	100.0 ± 0.0	0.0 ± 0.0
**ASA**	10.2 ± 0.7	89.8 ± 0.7
**3a**	8.9 ± 6.7	91.9 ± 6.7
**3b**	90.6 ± 9.4	9. 6 ± 9.4
**3c**	100.0 ± 0.0	0.0 ± 0.0
**3d**	100.0 ± 0.0	0.0 ± 0.0
**3e**	100.0 ± 0.0	0.0 ± 0.0
**3f**	91.4 ± 4.4	8.6 ± 4.4
**3g**	6.9 ± 10.1	93.1 ± 10.1
**3h**	93.3 ± 5.1	6.7 ± 5.1
**3i**	91.4 ± 5.8	8.6 ± 5.8
**3j**	8.3 ± 0.5	91.7 ± 0.5
**3k**	90.1 ± 11.2	9.9 ± 11.2
**3l**	100.0 ± 0.0	0.0 ± 0.0
**3m**	100.0 ± 0.0	0.0 ± 0.0
**3n**	100.0 ± 0.0	0.0 ± 0.0
**3o**	92.4 ± 12.1	7.6 ± 12.1
**3p**	5.5 ± 1.8	94.5 ± 1.8

Table S2. Concentration required to inhibit 50% of arachidonic acid (AA)-induced platelet aggregation (IC_{50}) for piperazine thiourea derivatives compared to acetylsalicylic acid (ASA). Data are expressed as the mean ± standard deviation (SD) (n = 3).

Compound	IC_{50} values [µM]			Mean
**ASA**	38.59	38.78	31.48	36.28 ± 4.16
**3a**	93.28	72.88	75.84	80.67 ± 11.02
**3g**	72.91	76.44	100.0	83.11 ± 14.7
**3j**	76.55	93.69	93.69	87.97 ± 9.9
**3p**	77.10	68.99	77.52	74.54 ± 4.8

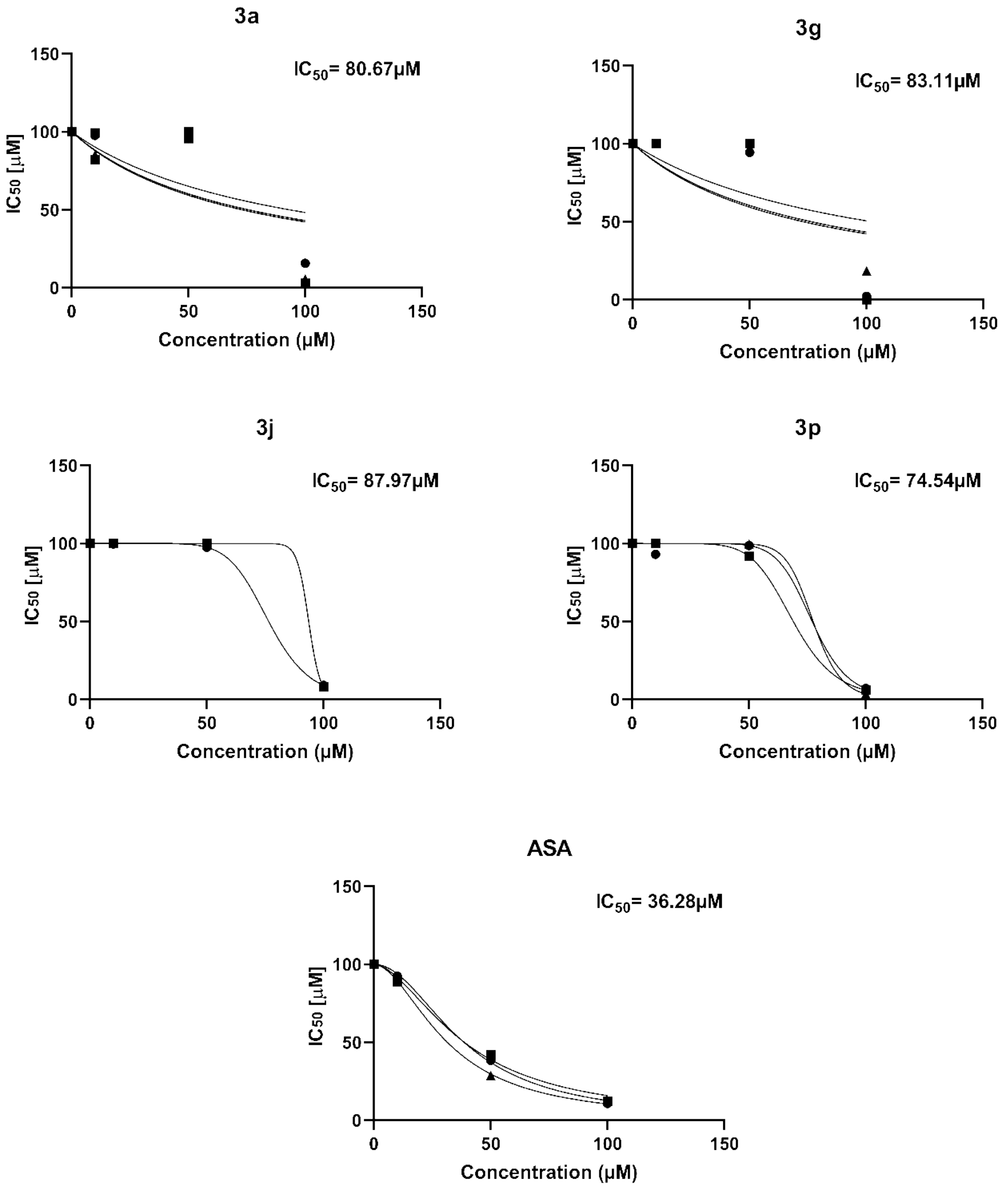

Figure S56. Dose–response curves used to determine the IC_{50} values for piperazine–thiourea derivatives **3a**, **3g**, **3j**, and **3p**, compared with acetylsalicylic acid (ASA). Data are expressed as mean ± standard deviation (SD) (n = 3).
